# The influence of the food environment on diet quality: Insights from an extensive household survey in Ethiopia, focusing on women of reproductive age

**DOI:** 10.1186/s40795-025-01097-z

**Published:** 2025-06-02

**Authors:** Andinet Abera Hailu, Stephen Thornhill, Masresha Tessema, Alebel Bayrau Weldesilassie, Edward Lahiff

**Affiliations:** 1 Department of Nutrition, Environment and Noncommunicable Diseases Research at, Ethiopian Public Health Institute, Addis Ababa, Ethiopia; 2 University College Cork, Cork, Republic of Ireland; 3 Policy Studies Institute, Addis Ababa, Ethiopia

**Keywords:** Food environment, Food access, Diet quality, Household, Women of reproductive age, Food systems, Ethiopia

## Abstract

**Background:**

Undernutrition is a significant challenge in Ethiopia, where limited dietary diversity and widespread micronutrient deficiencies affect millions, especially women of reproductive age. Although much research has explored dietary gaps and practices, the impact of the food environment (FE) on diet quality remains understudied. This study examines how FE factors influence dietary quality, using a food systems approach to inform sustainable nutrition policies.

**Methods:**

A cross-sectional survey of 1,828 households was utilized to assess women’s dietary quality using Poisson regression, with food environment (FE) components and socioeconomic variables predicting four indicators: women’s diet diversity score (WDDS), fruit and vegetable score (FVS), global dietary quality score (GDQS), and household diet diversity score (HDDS).

**Results:**

Dietary diversity was low, with fewer than 20% of women meeting the minimum recommendation; however, improvement was observed with higher quality food environments (FE). Starchy staples dominated consumption across all FE, while pulses and dark green leafy vegetables increased in households with medium and high FE. Low household dietary diversity (average 6.23) and a GDQS of 20.7 (far below the maximum of 49) indicate widespread deficiency. Poisson regression (adjusted for socioeconomic covariates) showed that a high FE score significantly predicted better diet quality compared to a low FE score: 27% higher WDDS (exp(β) = 1.27, 95% CI: 1.22–1.32, *p* < 0.001), 43% higher FVS (exp(β) = 1.43, 95% CI: 1.33–1.54, *p* < 0.001), 5% higher GDQS (exp(β) = 1.05, 95% CI: 1.02–1.08, *p* < 0.001), and 48% higher HDDS (exp(β) = 1.48, 95% CI: 1.43–1.54, *p* < 0.001). The results demonstrate consistent and significant associations between higher food environment (FE) scores and improved diet quality across all four dietary metrics.

**Conclusions:**

This study demonstrates the critical influence of food environments—encompassing market food diversity, physical access (e.g., food availability), economic access (e.g., affordability), and supportive infrastructure (e.g., roads, transportation, financial services)—on improving dietary quality among Ethiopian women and household food security. High food prices, socioeconomic disparities, and regional variations limit access to nutrient-rich foods (e.g., fruits, vegetables, animal-sourced products), particularly for low-income households. To enhance dietary quality and health outcomes, policymakers should prioritize interventions that expand diverse food markets, strengthen rural infrastructure (roads, transportation), and improve affordability through targeted economic support and price stabilization, ensuring equitable access to nutritious foods.

**Trial registration:**

Not applicable.

**Supplementary Information:**

The online version contains supplementary material available at 10.1186/s40795-025-01097-z.

## Background

The burden of malnutrition, including stunting, wasting, and micronutrient deficiencies, remains a persistent public health issue in Ethiopia, worsened by limited dietary diversity and insufficient intake of essential micronutrients [1–3]. Several studies have shown that an inadequate diet is often associated with limited access to diverse foods due to a range of factors, such as poverty, lack of education, climate and weather variability, conflict, and other sociocultural barriers [4, 5]. These dietary inadequacies often involve complex, interacting factors, including limited access to diverse and affordable foods, suboptimal food environments, and socioeconomic disparities [6–10].

The food environment (FE), a crucial component of the broader food system, is defined by the intricate interplay of physical, economic, and sociocultural factors that ultimately shape dietary patterns at the household level [11, 12]. These encompass not only the physical availability and accessibility of diverse food but also the affordability of various food options, the prevailing cultural norms and traditions surrounding food consumption, and the market functioning and access that influence people’s diet and nutrition [13, 14]. Consequently, there is growing research interest in understanding how these multifaceted aspects of the food environment impact dietary quality. For instance, several studies emphasize that FE mediates dietary outcomes through both external factors (e.g., market access) and individual factors (e.g., purchasing power), providing a framework for analyzing these dynamics in low-income settings [13, 15, 16].

A growing body of evidence highlights the critical role of the FE in shaping dietary quality, linked in a complex way to socioeconomic factors at both individual and household levels [17–19]. Individuals with higher socioeconomic status (SES) often reside in areas with better access to diverse food options, supported by higher disposable incomes that enable them to purchase a variety of foods from markets or, in rural settings, to cultivate diverse crops [20, 21]. Evidence from low- and middle-income countries (LMICs) shows that higher SES is associated with improved market access, better infrastructure, and the capacity to invest in agricultural inputs, all contributing to enhanced dietary diversity [22, 23]. In contrast, those with lower SES may be constrained to producing or purchasing less diverse and less nutritious foods, often relying on staple crops with limited nutritional value. This disparity in access and affordability results in significant differences in dietary quality, exacerbating the double burden of malnutrition prevalent in many LMICs, including Ethiopia [24].

The precise relationship between the food environment (FE) and dietary quality is not fully understood, particularly in resource-constrained settings [16]. Research on individual dimensions of the FE dimension, such as the availability of healthy foods, correlates with increased consumption of fruits and vegetables and improved overall diets [25]. However, dietary behaviors, cultural preferences, and perceptions also substantially shape dietary outcomes [26]. Similarly, Herforth and Ahmed [8] suggest that interventions targeting the FE could improve dietary intake by enhancing access to nutrient-rich foods, especially among vulnerable groups such as women of reproductive age. However, challenges such as limited market access, market food diversity, supporting infrastructure availability, food prices, dependence on subsistence agriculture, and other key aspects of the FE elements intensify socioeconomic disparities, particularly among low-income households in Ethiopia [22]. Despite these insights, a comprehensive study of the food environment remains a notable research gap, particularly in the Ethiopian setting.

Inadequate dietary diversity [27–29] and insufficient nutrient intake [30, 31] are prevalent in Ethiopia, particularly among women, with the prevalence varying across regions and socioeconomic groups. Diet-related risks, such as low fruit and vegetable consumption, are among the country's leading contributors to disease and mortality, particularly for vulnerable populations [32]. While extensive research has explored dietary practices and nutrient deficiencies [25–29], no study, to our knowledge, has attempted to comprehensively examine the food environment’s role in shaping dietary patterns among Ethiopian women and households. Evidence remains limited on how specific or combined dimensions of the food environment, such as physical and economic access to diverse foods, affect dietary quality in this context. The High Level Panel of Experts (HLPE) highlights that food environments in low-income countries like Ethiopia are often constrained by poor infrastructure and market inefficiencies, underscoring the need for targeted research [33].

This study addressed critical gaps in the literature by examining the multifaceted dimensions of the food environment and their impact on dietary quality among women of reproductive age in Ethiopia, a group often underrepresented in nutritional research. To explore these dynamics, we investigated key aspects of the food environment, including availability, physical accessibility, price, affordability, and the infrastructure supporting these elements, as well as their relationship to dietary quality indicators in Ethiopia, focusing on women of reproductive age who are disproportionately affected by nutritional disparities. Our approach began by assessing household food consumption patterns and women’s dietary quality. We then applied a comprehensive food environment framework to evaluate the landscape across the studied settings, providing insights into factors that shape food access. Finally, we analyzed associations between food environment characteristics and dietary quality measures to uncover pathways influencing women’s nutrition. This study generated actionable evidence to guide targeted interventions and policies that enhance dietary quality and improve nutritional outcomes in Ethiopia.

## Methods

### Study setting

**Fig. 1:**
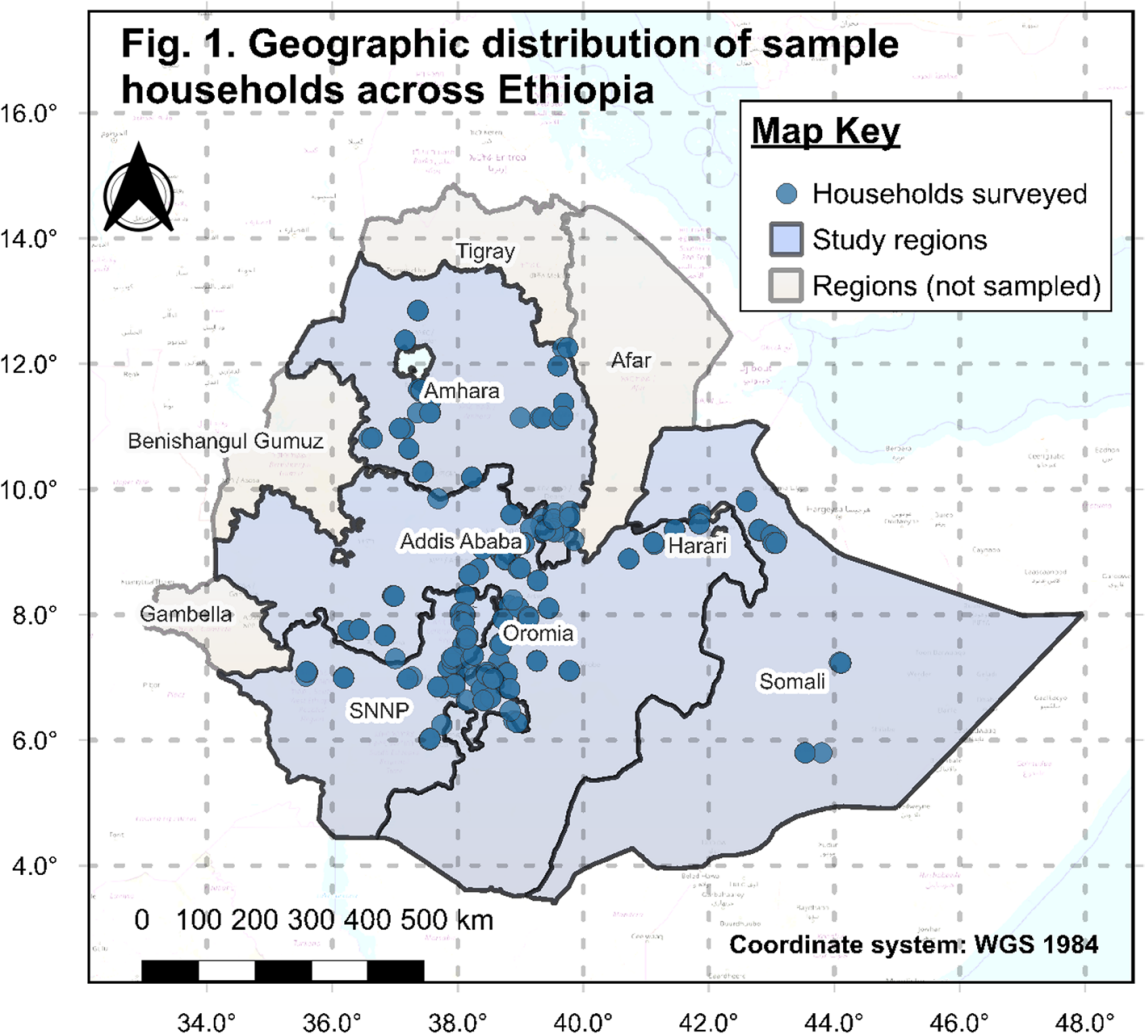
Geographic distribution of sample households across Ethiopia*.* Circle symbols represent interviewed households. Sampled and non-sampled regions are indicated

A cross-sectional household survey was conducted from August 25 to September 30, 2022, using a structured questionnaire with various modules across five regions and one city administration in Ethiopia: Amhara, Oromia, Somalie, Southern Nations, Nationalities, and Peoples (SNNPR), Sidama, and Addis Ababa. Sampling locations were selected to represent a range of food environment (FE) characteristics, ensuring diversity in dietary habits and urban and rural settings. This approach ensures that the study's findings broadly reflect the Ethiopian population. Figure 1 illustrates the distribution of sample households and regions.

### Sample size determination and sampling procedure

The sample size for this survey, targeting women of reproductive age (WRA), was determined using a single population proportion formula. The calculation assumed a 95% confidence level (Z = 1.96), a relative standard error of 0.05, and a design effect of 1.5 to account for clustering [34]. Additional adjustments were made based on regional household sizes, the proportion of the target population in each region, and an anticipated 95% household response rate. To ensure regional representation, a minimum of 1,812 WRAs was required. However, to accommodate an expected 5% non-response rate, the sample size was increased to 1,908 households, calculated using the average number of target cases per household [34]. The sample size was derived using the following formula (Eq. 1):

1 $$n=\frac{\left[{Z^2}_\frac\alpha2\ast P\ast\left(1-P\right)\right]}{d^2}\ast Deff\ast\left(\frac{100}{HHRR}\right)\ast\left(\frac{100}{IRR}\right)\ast\left(\frac1{HH}size\right)\ast(\frac1{\%Target})$$

where:

- n = Required sample size = 1812
- Zα/2 = 1.96 (standard error for a 95% confidence level)
- P = Minimum dietary diversity for women (MDD-W) prevalence
- d = Allowable error (0.05)
- Deff = Design effect (1.5)
- Ave. HH Size = Average household size per region
- % Target PP = Proportion of the target population per region
- HHRR = Household response rate (%)
- IRR = Individual response rate (%)

Households were selected using a multistage sampling approach based on the Ethiopian 2020/2021 Household Income and Expenditure Survey Framework [35]. The sampling process began with a purposive selection of administrative zones and woredas to distribute the sample across the country [36], reflecting Ethiopia’s diverse agroecological zones and farming systems. This purposive selection ensured representation of various socio-economic backgrounds and agricultural contexts. Subsequently, kebeles and villages within these units were randomly selected. The data collection team enumerated all households in the chosen villages and systematically sampled 20 households per village [37]. To align with the national urbanization rate, 30% of the sampled kebeles were drawn from urban areas and 70% from rural regions, proportionate to Ethiopia’s population distribution rather than geographic equality or population size alone [36]. This approach balanced urban and rural representation while capturing the socio-economic diversity inherent in these settings. In eligible households, interviews were conducted with household heads and one woman aged 15 to 49 years per household.

### Data source and management

Household survey data were collected from 1,828 households in Ethiopia, targeting women of reproductive age (15–49 years). A comprehensive household questionnaire captured dietary intake, socioeconomic factors, and food environment variables (Additional file 4). Data collection took place between August and September 2022, employing computer-assisted personal interviewing (CAPI) with CSPro software installed on smartphones. Trained enumerators conducted the interviews, ensuring real-time data entry and validation, which minimized errors during fieldwork [38].

To complement household-level data, food price data were sourced from the Ethiopian Central Statistical Service’s monthly Consumer Price Index (CPI), covering five months from July to November 2022 [39]. This dataset included prices for 119 food commodities—spanning staples (e.g., grains, roots), nutrient-rich foods (e.g., fruits, vegetables, dairy, eggs), and other groups (e.g., pulses, meat)—which enabled the calculation of average food group costs across study zones. These temporal price data were matched to the survey period to assess the impact of food prices and affordability on dietary outcomes. Additionally, household food expenditure data at the regional level were extracted from the Ethiopian Socioeconomic Survey (ESS), a nationally representative panel survey [40], and integrated with survey data. The ESS provided aggregated food expenditure estimates, offering a broader economic context for interpreting a household’s affordability of a nutritious diet [41].

Data were managed and analyzed in STATA version 16 [42] after thorough cleaning and verification for quality. Incomplete observations were excluded rather than imputed to maintain model robustness, as imputation risked introducing bias given the dataset’s complex interrelationships [43].

### Diet quality assessment

We assessed women’s diets using the Diet Quality Questionnaire (DQQ), adapted for Ethiopia [44]. The DQQ tool comprises 29 food items or specific categories consumed over the previous 24 h. In this research, we utilized four diet quality indicators: Women’s Dietary Diversity Score (WDDS), a simple Fruit and Vegetable Score (FVS) derived from DQQ, a Global Diet Quality Score (GDQS), and Household Dietary Diversity Score (HDDS). These indicators were selected based on suitability for the cross-sectional survey and established correlation with nutrient adequacy [45].

The Women’s Dietary Diversity Score (WDDS), following the FAO’s MDD-W protocol [45], measures 24-h dietary diversity across 10 food groups, with scores ranging from 0 to 10. A WDDS of five or more food groups suggests better micronutrient adequacy [46–48]. The FVS, with scores from 0 to 6, assesses fruit and vegetable food groups and has also been shown to correlate with micronutrient adequacy [49]. The GDQS, a 25-food-group metric (scored 0–49), evaluates nutrient adequacy and NCD risk using a food frequency approach [50]. Supplementary Table S2 presents GDQS scoring and mean intake by food group among women of reproductive age (Additional file 1). The HDDS, based on 16 FAO-defined food groups, measures household food variety and access [51].

These indicators were all assessed based on a 24-h recall period. Despite the potential for recall bias, particularly with infrequent items [52], these indicators were selected for their feasibility in the cross-sectional study and adherence to DQQ standards [53]. While repeated measures could more effectively capture habitual intake [54], employing multiple indicators could help reduce bias and improve the evaluation of how food environment predictors influence dietary quality [55, 56].

### Assessment of the food environment

**Fig. 2:**
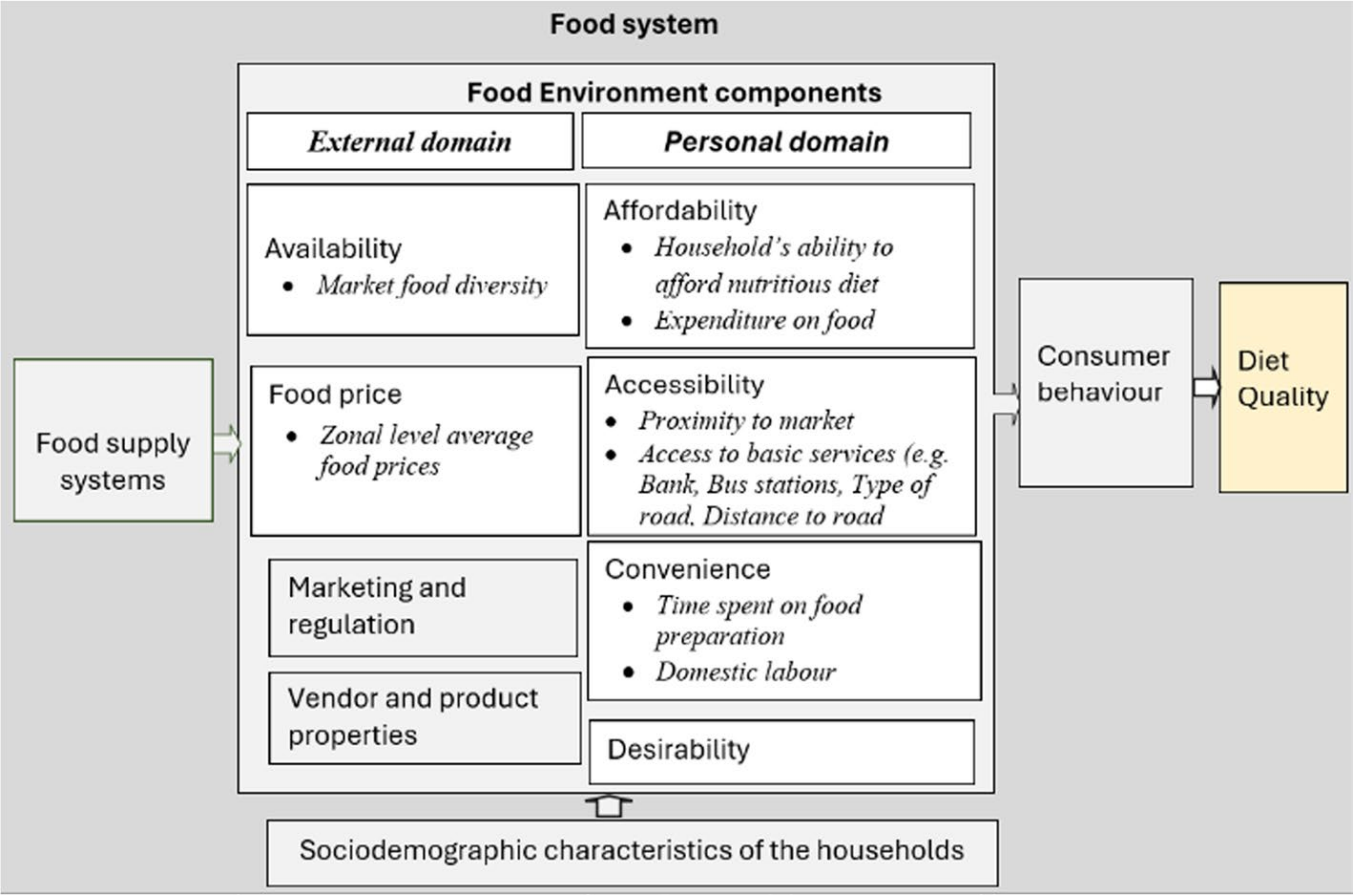
A conceptual framework illustrating the food environment within the food system. (Adapted from Turner et al. [16])

We adapted and applied a conceptual framework for low- and middle-income settings [16] and the broader food systems framework [33] to evaluate the food environment. This framework classifies the food environment into two main domains: external and personal, making it suitable for low- and middle-income countries [16]. The external domain encompasses food availability, prices, vendor and product characteristics, marketing, and regulation. The personal domain includes individual-level factors such as food accessibility, affordability, convenience, and desirability. This distinction highlights how broader market dynamics and personal circumstances influence diets. Figure 2 illustrates the conceptual framework guiding the study in investigating the relationships among different components of the food environment (FE) and dietary outcomes (Fig. 2). The framework facilitated the creation of a composite score for food availability, affordability, accessibility, price, convenience, and desirability by integrating individual scores. Due to data limitations, certain aspects of the theoretical framework (e.g., vendor/product characteristics, marketing/regulation) remain unaddressed (Fig. 2).

#### Food availability

Participants reported the presence of food items from a list of 58 within a 60-min walk from their residences [25]. This list was then categorized into ten groups according to the FAO’s WDDS guidelines [45]. A score reflecting the number of available food groups was classified as low (≤ 4 groups), medium (5–7 groups), and high (8–10 groups) availability.

#### Price, expenditure, and affordability

Food prices were analyzed over a five-month period centered on the interviews. Prices for each food group were averaged by study zone and categorized into tertiles (low, medium, high) based on the overall distribution. A final food price score (1 to 3) was derived for each zone by averaging the scores across food groups [57]. Affordability was defined as a household’s capacity to obtain a nutrient-adequate diet using locally available foods [41]. Non-affordability was evaluated by comparing the household's average monthly food expenditure to the cost of a nutritious diet for that zone, classified as low, medium, and high [58].

#### Physical accessibility

Accessibility refers to the ease with which individuals can acquire food [16]. In this analysis, the variables related to “physical access” consisted of the reported walking distance to the nearest market, distance to roads, transportation facilities, banking services, and types of roads. These variables were used to determine whether households had a reasonable opportunity to access the resources needed to obtain the food necessary for a healthy diet.

#### Convenience

This study defines convenience as the ease and speed with which individuals can acquire and prepare food, acknowledging its potential to enhance or impair food choices and diet quality [59]. To operationalize convenience, we evaluated two key factors within the household context: the time allocated to food preparation (measured in minutes per meal, as reported by respondents) and the availability of domestic labour dedicated to food preparation-related tasks.

#### Desirability

Participants ranked their top three food preferences (1 = most preferred, 2 = second, 3 = third) under the assumption of no financial or health constraints. They then reported the frequency of consuming these foods over the previous 24 h. A desirability score was calculated by weighting preferences (3, 2, 1 point for first, second, and third choice) and multiplying this by the frequency of consumption, with higher scores indicating a greater alignment between diet and preferences [60, 61].

#### Food environment composite score

A composite score for the food environment for each household was developed by integrating scores for food availability, affordability, price, access, convenience, and desirability. This composite score was then categorized into three levels of food environments [57]: low, medium, and high, corresponding to scores of 1, 2, and 3, respectively.

### Econometric approach

The wealth index was constructed using principal component analysis (PCA) of household asset ownership (including livestock), following methods explained elsewhere [62, 63].

To investigate the relationship between dietary quality indicators—Women’s Dietary Diversity Score (WDDS), Fruit and Vegetable Score (FVS), Global Diet Quality Score (GDQS), and Household Diet Diversity Score (HDDS)—and the food environment, we employed a series of Poisson regression models, a method widely used for count outcomes in nutritional studies [64]. We first estimated a baseline model (Model 1) using sociodemographic characteristics such as residence (urban/rural), gender, age, education level, region, livelihood type, and wealth index, to establish baseline associations, following standard practice in dietary research [51]. Model 2 examined food environment dimensions alone, capturing external factors (e.g., the number of food groups available in nearby markets, average prices) and personal factors (e.g., household expenditure, non-affordability, access to infrastructure like markets and roads), consistent with frameworks for food environment analysis [16]. Model 3 combined all predictors to assess their joint influence on dietary outcomes, while Model 4 used a composite food environment score alongside sociodemographic covariates as an alternative approach, a method applied in similar studies to simplify complex food environment effects [8].

For each diet quality indicator Yk (where k represents WDDS, FVS, GDQS, and HDDS), we modeled the log of the expected count using Poisson regression. The full model (Model 3) is specified as:

2 $$\begin{array}{c}\text{log}\left(\text{E}\left[Y_k\right]\right)=\alpha k+\beta_{k1}X_1+\beta_{k2}X_2\dots+\beta_{km}X_m+\gamma_{k1}\;Z_1\;+\;\gamma_{k2}Z_2\;+\;\cdots+\gamma_{kn}\;Z_n\end{array}$$

where: X_1_, X_2_,…, X_m_ are sociodemographic variables, Z_1_, Z_2_,…, Z_n_ are food environment variables (external and personal), and α_k_, β_kj_, γ_kj_ are the intercept and coefficient specific to outcome k [65]. Equivalently, the expected count can be specified as:

3 $$E\lbrack\begin{array}{c}Y_k\rbrack=exp\lbrack\alpha k+\beta_{k1}X_1+\beta_{k2}X_2\dots+\beta_{km}X_m+\gamma_{k1}Z_1+\gamma_{k2}Z_2+\dots+\gamma_{kn}Z_n)\end{array}$$

Model 4 simplifies the food environment dimensions into a composite score, specified as:

4 $$\text{log}\left(\text{E}\left[Y_k\right]\right)=\alpha k+\beta_{k1}X_1+\beta_{k2}X_2+\dots+\beta_{km}X_m+\delta_kW$$

W is the composite food environment score, and δ_k_ is its coefficient. Coefficients (exp (β_kj_), exp (γ_kj_), exp(δ_k_)) represent the multiplicative effect on the expected count of indicator k per unit increase in the respective predictor [62]. Estimated coefficients were deemed significant at *p* < 0.05, with additional thresholds at *p* < 0.01 and *p* < 0.001 for robustness.

This model was selected for its suitability for count response variables and its ability to manage both continuous and categorical predictors within the food environment framework [61]. Model assumptions were evaluated: goodness-of-fit was confirmed through the deviance statistics, and overdispersion was assessed using the ratio of residual deviance to degrees of freedom. No significant overdispersion was identified (*p* > 0.05), supporting the Poisson model over a negative binomial alternative [61]. Multicollinearity was evaluated through variance inflation factors (VIFs), all below 10, indicating no significant multicollinearity [66].

## Results

### Description of household characteristics

Table 1 outlines descriptive statistics of the demographic and dietary characteristics of the households and women included in the study. Overall, 63.6% of respondents resided in rural areas and 38.4% in urban areas. The average age of the household heads was 42 years, and 18% were female. On average, households consisted of five members. In urban areas, about fifty percent of household heads have completed high school or attained a higher level, while only twenty percent of household heads in rural areas have achieved this level of education.

**Table 1:** Sociodemographic and dietary characteristics of study participants by residence type, Ethiopia (*N* = 1,828)

Characteristic	Total (*N* = 1,828)	Rural (*n* = 1,126)	Urban (*n* = 702)
Regions, n (%)			
Amhara	370 (20.3)	285 (25.4)	85 (12.1)
Oromia	504 (27.6)	365 (32.5)	139 (19.8)
Somalie	134 (7.2)	97 (8.5)	37 (5.3)
SNNP	382 (20.9)	300 (26.7)	82 (11.7)
Sidama	133 (7.3)	79 (7.0)	54 (7.7)
Addis Ababa	305 (16.7)	0 (0.0)	305 (43.4)
Gender, n (%)			
Female	335 (18.3)	147 (13.1)	188 (26.8)
Male	1,493 (81.7)	979 (86.9)	514 (73.2)
Age, mean (SD)	41.6 (12.2)	40.5 (11.4)	43.4 (13.2)
Family size, mean (SD)	4.7 (1.8)	4.9 (1.8)	4.6 (1.8)
Education attained, n (%)			
Primary (1–8 years)	1,221 (66.8)	869 (77.1)	352 (50.1)
Completed high School	331 (18.1)	134 (11.9)	197 (28.1)
High school + certificate	85 (4.7)	36 (3.2)	49 (7.0)
Bachelor and above	141 (7.7)	54 (4.8)	87 (12.4)
Other informal	50 (2.7)	33 (2.9)	17 (2.4)
Wealth tertiles, n (%)			
Poorest	610 (33.4)	441 (39.1)	169 (24.1)
Middle	615 (33.7)	276 (24.6)	339 (48.3)
Richest	603 (32.9)	409 (36.2)	194 (27.6)
Dietary scores, mean (SD)			
WDDS	3.4 (1.5)	3.3 (1.4)	3.4 (1.6)
HDDS	6.23 (0.05)	5.8 (0.1)	6.7 (0.1)
FVS	1.3 (1.3)	1.3 (1.2)	1.3 (1.4)
GDQS	20.7 (0.12)	20.5 (0.17)	21.0 (0.18)
MDD-W, n (%)	362 (19.8)	215 (19.1)	147 (20.9)
Food environment (FE), n (%)			
Low FE	183 (37.6)	165 (48.5)	18 (20.1)
Medium FE	1,236 (40.0)	783 (35.7)	453 (47.0)
High FE	409 (22.4)	178 (15.8)	231 (32.9)

Data are presented as n (%) for categorical variables and mean (SD) for continuous variables

*Abbreviations* *SD* standard deviation, *HH* household, *NA* not applicable

### Women’s diet quality and the food environment (FE)

**Fig. 3:**
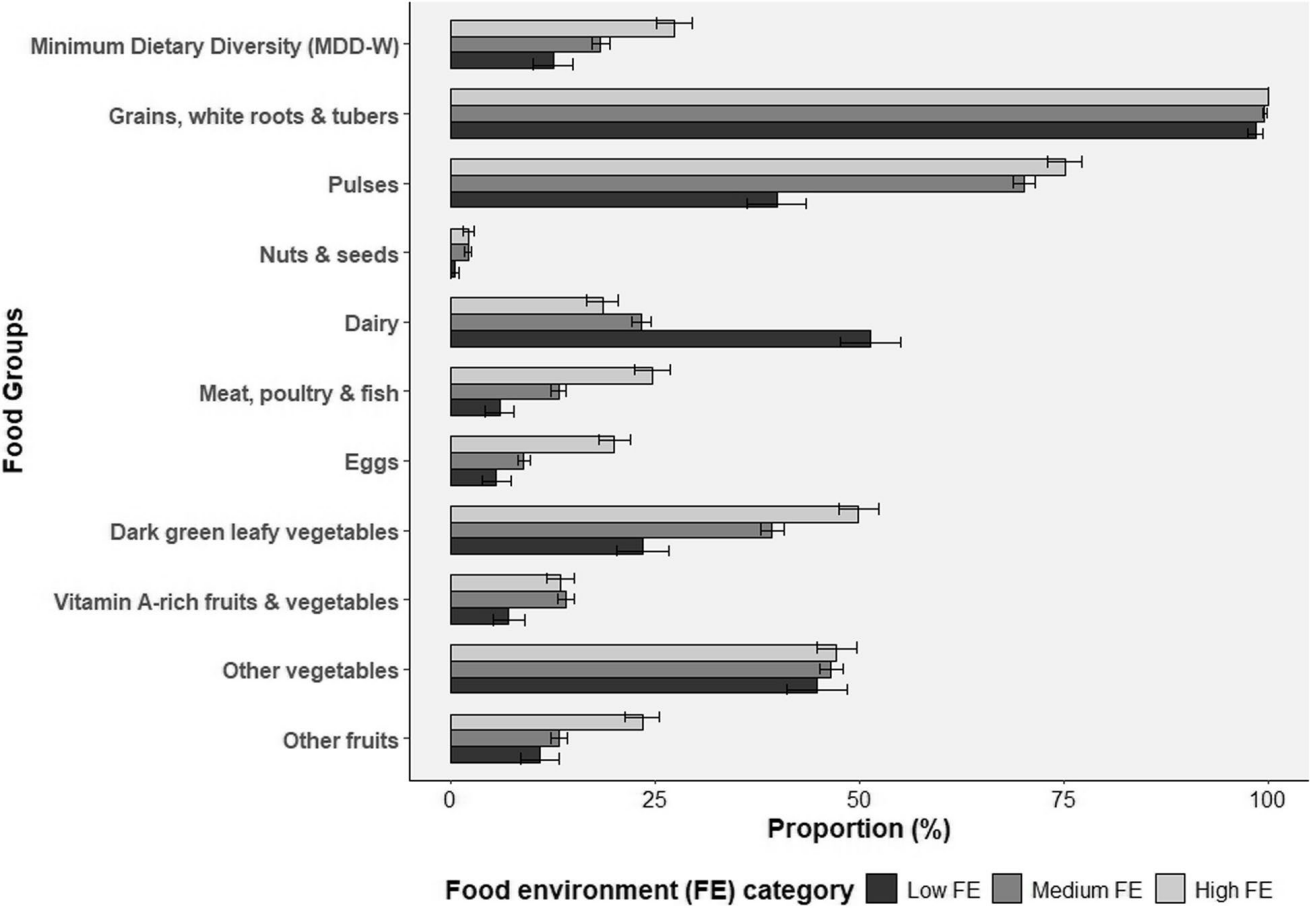
Women's consumption of food groups by Food Environment (FE) category. Bars represent the proportion of women consuming each food group, categorized by Food Environment (FE) levels: Low, Medium, and High. Error bars indicate standard errors of the proportions

Figure 3 illustrates women's consumption of food groups across food environment (FE) categories. Grains, white roots, and tubers were consumed by nearly all women (approximately 99%) across all FE categories, followed by pulses (40% in low FE, 70% in medium FE, and 75% in high FE). Other vegetables and dark green leafy vegetables were consumed by 45–47.2% and 23.5–50% of women, respectively, with slight increases from low to high FE. Dairy consumption was higher in low FE (51%) compared to medium (23.3%) and high FE (19%), while meat, poultry, fish, and eggs showed low consumption but increased slightly with higher FE. Vitamin A-rich fruits and vegetables, other fruits, nuts, and seeds had the lowest consumption (1% to 24%) with modest increases across FE categories. The graph also shows an improvement in the percentage of women achieving minimum dietary diversity from low to high FE.

**Fig. 4:**
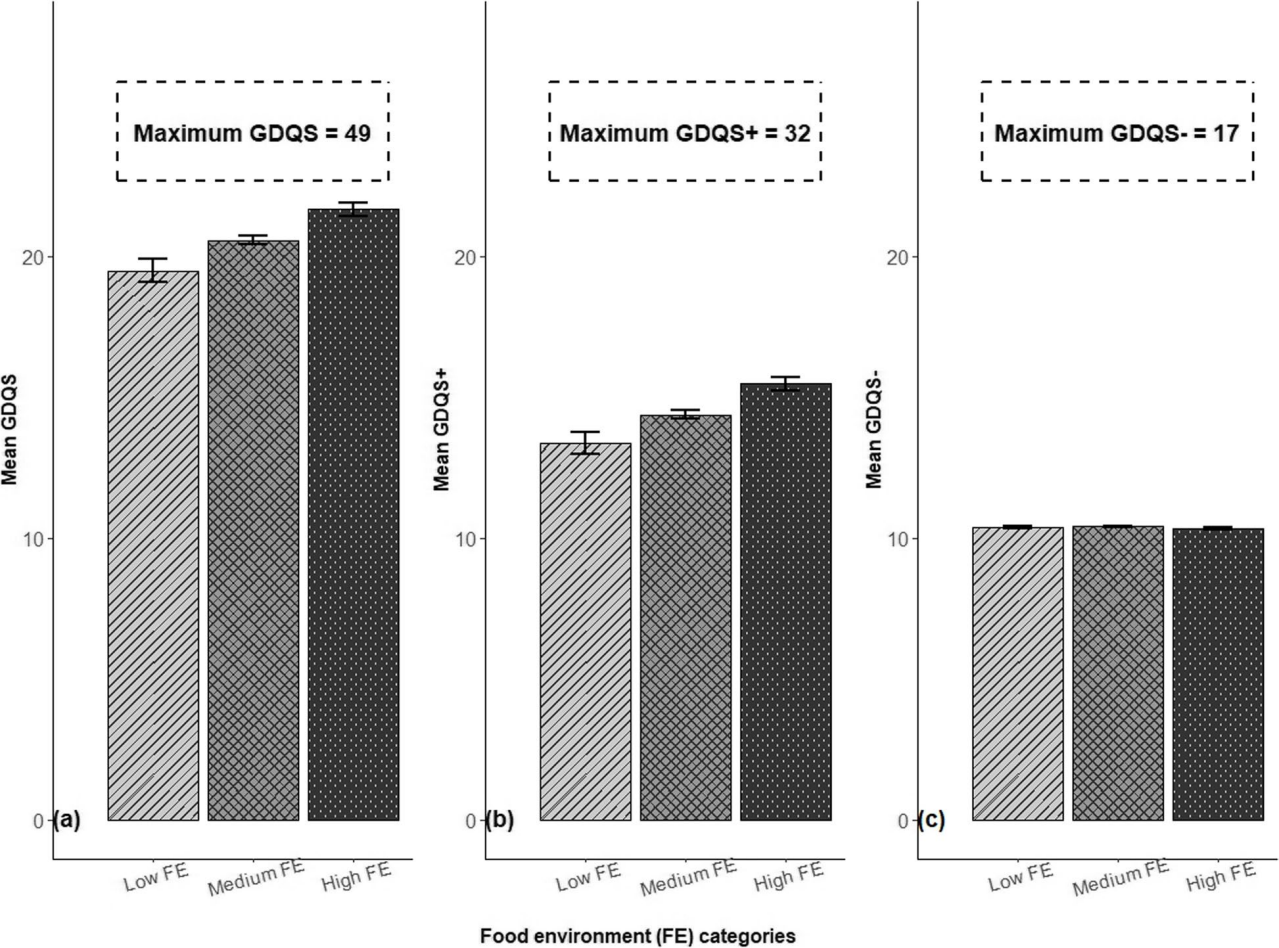
Women’s mean GDQS and subcomponents (GDQS + and GDQS-) by food environment (FE). Panels: **a** total GDQS (max score = 49); **b** GDQS + (healthy food groups, max score = 32); **c** GDQS- (unhealthy food groups, max score = 17)

Figure 4 presents the mean global diet quality score (GDQS) across food environment (FE) categories for 1,828 participants. The total GDQS increased from low to high FE (Fig. 4a); however, the observed means were notably lower than the maximum possible score of 49, suggesting significant dietary inadequacies. This trend of increasing scores with higher FE was consistent for both the GDQS + (healthy food groups, maximum score 32, Fig. 4b) and the GDQS- (unhealthy food groups, maximum score 17, Fig. 4c). For further analysis, GDQS intake was categorized into low, middle, and high based on established daily intake cutoff values (in grams), and the distribution of women within these categories is detailed in Supplementary Table S3 (Additional file 1).

**Fig. 5:**
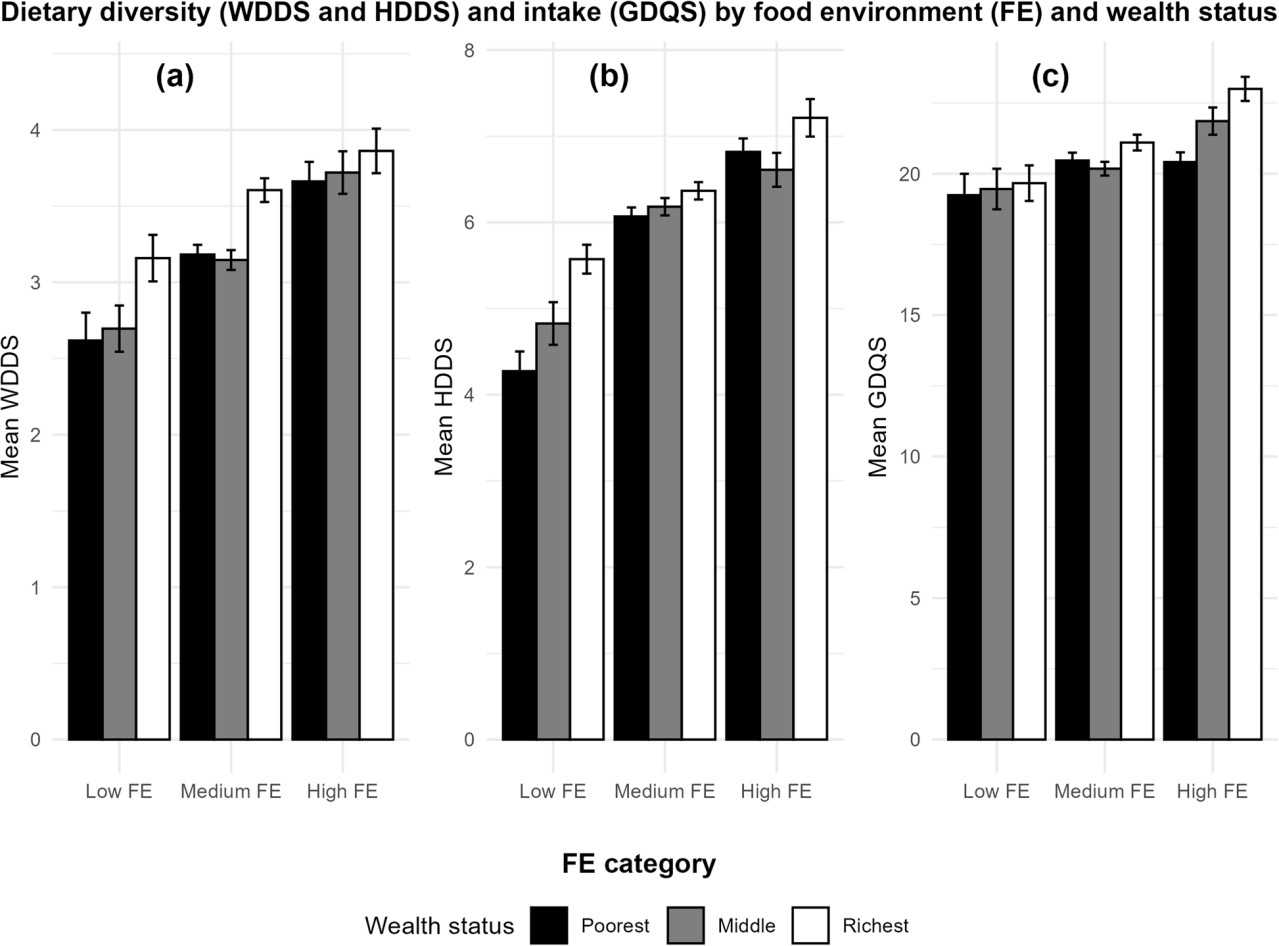
Dietary diversity (WDDS and HDDS) and intake (GDQS) based on the food environment (FE) and wealth status. Panels show (**a**) Mean women’s dietary diversity score (WDDS), **b** mean household dietary diversity score (HDDS), and (**c**) mean global diet quality score (GDQS). Bars represent scores for the poorest, middle, and richest wealth categories within each FE group

Dietary diversity and intake scores were analyzed based on Food Environment (FE) and wealth status (Fig. 5). The average Women’s Dietary Diversity Score (WDDS) increased across FE categories, with the wealthiest group demonstrating the highest scores (Fig. 5a). Similar trends were noted for the mean Household Dietary Diversity Score (HDDS) (Fig. 5b) and the average Global Diet Quality Score (GDQS) (Fig. 5c). The findings indicate that the wealthiest group exhibited the highest average scores for all indicators across each FE category.

### Sociodemographic and food environment predictors of diet quality

Table 2 presents the results of Poisson regression analyses that examine the associations between dietary quality indicators—Women’s Dietary Diversity Score (WDDS), Fruit and Vegetable Score (FVS), Global Diet Quality Score (GDQS), and Household Dietary Diversity Score (HDDS)—and a range of socioeconomic and food environment factors from a sample of 1,828 households and women. This section summarizes key findings regarding the socioeconomic and food environment predictors of these multiple diet quality indicators based on the fully adjusted Poisson regression model results. Supplementary Table S4 (Additional file 2) provides the full Poisson regression results. The model fit for WDDS, FVS, GDQS, and HDDS improved across specifications, with Model 3 (combined factors) demonstrating the strongest explanatory power. Model fit statistics can be found in Tables S5 and S6 (Additional file 2).

**Table 2:** Results from the Poisson regression analysis show associations between dietary quality, socioeconomic factors, and components of the food environment in Ethiopia (*N* = 1828)

Variables			(1) WDDS	(2) FVS	(3) GDQS	(4) HDDS
			exp(β)[ 95% CI]	exp(β)[ 95% CI]	exp(β)[ 95% CI]	exp(β)[ 95% CI]
Residence [Rural = Ref]			1.03 [0.97–1.11]	1.12 [0.97–1.28]	1.01 [0.97–1.04]	0.98 [0.93–1.02]
Gender [Female = Ref]			0.99 [0.94–1.04]	1.02 [0.90–1.14]	1.01 [0.98–1.04]	0.99 [0.96–1.03]
Education years attended			1.00 [1.00–1.01]	1.01 [1.00–1.01]	1.01 [1.00–1.01] ^*^	1.01 [1.00–1.01] ^*^
Region						
Amhara			0.92 [0.85–0.99] ^*^	0.62 [0.50–0.76] ^***^	1.00 [0.96–1.05]	0.83 [0.78–0.88] ^***^
Oromia			1	1	1	1
Somalie			0.70 [0.54–0.90] ^**^	0.63 [0.38–1.04]	0.95 [0.82–1.10]	0.88 [0.71–1.08]
SNNP			0.95 [0.87–1.03]	1.20 [1.00–1.43]	1.05 [1.00–1.11] ^*^	0.79 [0.74–0.84] ^***^
Sidama			0.92 [0.78–1.09]	0.64 [0.44–0.94] ^*^	0.90 [0.82–0.99] ^*^	0.93 [0.82–1.06]
Wealth status		Poorest	0.88 [0.83–0.92] ^***^	0.86 [0.77–0.96] ^**^	0.97 [0.94–1.00] ^*^	0.93 [0.90–0.96] ^***^
		Middle	0.92 [0.88–0.97] ^***^	0.91 [0.82–1.01]	0.98 [0.95–1.01]	0.9 [0.92–0.98]5^**^
		Richest	1	1	1	1
HH income decile (ETB)			1.01 [1.00–1.02] ^**^	1.02 [1.00–1.04] ^*^	1.01 [1.01–1.02] ^***^	1.01 [1.00–1.01]
Food availability		Low (< 4 FGs)	1	1	1	1
		Medium (5–7 FGs)	1.07 [1.00–1.15]	1.22 [1.01–1.46] ^*^	1.00 [0.90–1.01]	1.22 [1.15–1.30] ^***^
		High (8–10 FGs)	1.12 [1.04–1.18] ^**^	1.18 [0.99–1.41]	1.01 [0.88–1.02]	1.42 [1.35–1.50] ^***^
Average food group price		Grain, root, and tubers	1.09 [1.05–1.13] ^***^	1.28 [1.17–1.39] ^***^	1.05 [1.03–1.08] ^***^	0.99 [0.96–1.02]
		Vegetables	0.89 [0.85–0.94] ^***^	0.79 [0.70–0.89] ^***^	0.94 [0.91–0.97] ^***^	1.00 [0.96–1.04]
		Fruits	1.00 [0.97–1.02]	1.02 [0.96–1.09]	0.98 [0.96–0.99] ^**^	1.03 [1.01–1.05] ^**^
		Meats	1.01 [1.01–1.02] ^***^	1.04 [1.03–1.06] ^***^	1.01 [1.01–1.01] ^***^	1.00 [1.00–1.01]
		Milk	1.00 [1.00–1.01] ^*^	1.00 [0.99–1.00]	1.00 [1.00–1.00]	1.00 [1.00–1.00]
		Pulses, nuts, and seeds	0.94[0.89–1.00] ^*^	0.98 [0.85–1.13]	1.02 [0.99–1.05]	0.99 [0.95–1.03]
		Eggs	1.02 [1.00–1.03] ^*^	1.02 [0.99–1.06]	1.00 [0.99–1.01]	1.00 [0.99–1.02]
Expenditure decile (ETB)			1.01 [1.00–1.02] ^**^	1.00 [0.98–1.02]	1.01 [1.00–1.01] ^*^	1.02 [1.01–1.03] ^***^
Non-affordability (NA)		High NA	1	1	1	1
		Medium NA	1.03 [0.97–1.10]	1.17 [1.01–1.35] ^*^	1.01 [0.98–1.05]	0.97 [0.92–1.02]
		Low NA	1.01 [0.92–1.11]	1.33 [1.08–1.65] ^**^	1.04 [0.98–1.09]	1.01 [0.85–1.03]
Distance to market–km			.99 [0.99–1.00] ^*^	0.99 [0.98–1.00] ^*^	.98 [0.97–1.00] ^**^	1.00 [1.00–1.00]
Distance to the road–km			1.00 [1.00–1.00]	0.99 [0.99–1.00] ^**^	1.00 [1.00–1.00] ^***^	1.00 [1.00–1.00] ^***^
Distance to bus station–km			1.00 [0.98–1.00] ^**^	1.00 [0.99–1.00]	1.00 [1.00–1.00]	1.00 [1.00–1.00]
Distance to a bank–km			1.00 [1.00–1.01]	1.01 [1.00–1.02]	1.00 [1.00–1.00]	1.00 [1.00–1.00]
Type of road access	No road within 1 h		1	1	1	1
	Gravel/all-weather		1.12 [1.07–1.18] ^***^	1.20 [1.08–1.34] ^***^	1.01 [0.98–1.04]	1.03 [0.99–1.07]
	Dry weather road		1.04 [0.99–1.10]	1.06 [0.95–1.19]	1.00 1.00 [0.97–1.02]	1.02 [0.98–1.05]
Convenience score			1.06 [1.00–1.11] ^*^	1.18 [1.06–1.32] ^**^	1.02 [0.99–1.06]	1.11 [1.06–1.15] ^***^
Desirability score			1.14 [1.10–1.19] ^***^	1.31 [1.19–1.45] ^***^	1.09 [1.07–1.12] ^***^	1.03 [1.00–1.07]
Model *P*-value			< 0.001	< 0.001	< 0.001	< 0.001
***N***			1818	1816	1818	1818

Model 3 includes sociodemographic covariates, external food environment (FE), and personal FE components. Results are expressed as incidence rate ratios (exp(β)), with 95% confidence intervals in brackets

Significance levels are indicated: *p < 0.05, **p < 0.01, ***p < 0.001. Sample sizes vary due to missing data

*Abbreviations* NA: Non-affordability; ETB: Ethiopian Birr. Full results for Models 1–3 are available in Additional file 2

### Sociodemographic predictors

The regression analysis highlights significant regional variations in dietary diversity across Ethiopia, as detailed in Table 2. Compared to Oromia, which serves as the reference region, the Amhara and Somalie regions exhibit significantly lower dietary diversity scores. In Amhara, the WDDS is reduced by 8% (exp(β) = 0.92, *p* < 0.05), the FVS decreases by 38% (exp(β) = 0.62, *p* < 0.001), and the HDDS is lower by 17% (exp(β) = 0.83, *p* < 0.001). These findings highlight considerable disparities in dietary quality among the regions. Similarly, in Somalie, the WDDS is 30% lower (exp(β) = 0.70, *p* < 0.01), indicating a significant challenge in achieving diverse diets. Other regions, such as SNNP and Sidama, present mixed outcomes: SNNP exhibits a relatively stable WDDS and a higher FVS (exp(β) = 1.20, *p* < 0.05) when compared to a reference. In contrast, Sidama demonstrates a lower FVS (exp(β) = 0.64, *p* < 0.05) along with a slightly reduced GDQS (exp(β) = 0.90, *p* < 0.05).

Beyond regional differences, wealth status and household income also influence dietary quality. Compared to the wealthiest category (reference), the poorest households exhibit reduced dietary diversity, with a 12% lower WDDS (exp(β) = 0.88, *p* < 0.001), a 14% lower FVS (exp(β) = 0.86, *p* < 0.01), and a 7% lower HDDS (exp(β) = 0.93, *p* < 0.001), along with a slight decrease in GDQS (exp(β) = 0.97, *p* < 0.05). Middle-wealth households also experience lower scores, with an 8% reduction in WDDS (exp(β) = 0.92, *p* < 0.001) and a 10% reduction in HDDS (exp(β) = 0.95, *p* < 0.01), while FVS and GDQS show no significant changes. Household income, measured in deciles of monthly income in Ethiopian Birr (ETB), positively correlates with dietary diversity, with a 1% increase in WDDS (exp(β) = 1.01, *p* < 0.01), a 2% increase in FVS (exp(β) = 1.02, *p* < 0.05), a 1% increase in GDQS (exp(β) = 1.01, *p* < 0.001), and a 1% increase in HDDS (exp(β) = 1.01, *p* < 0.05) per decile, highlighting the role of economic resources in improving dietary quality.

Other socioeconomic and demographic predictors, including residential status (rural as reference), show negligible effects, with only a modest 12% increase in FVS (exp(β) = 1.12) that lacks statistical significance. Gender also plays a minor role, with no substantial differences between males and females (reference) across all scores. Education, measured by years attended, demonstrates a minimal positive association, with a slight increase in GDQS (exp(β) = 1.01, *p* < 0.05) and HDDS (exp(β) = 1.01, *p* < 0.05), suggesting that additional education may offer a slight boost to dietary quality, though the effect is not pronounced. These findings underscore the dominant influence of regional context and economic status.

### Food environment predictors

#### External food environment

Dietary outcomes are significantly influenced by factors in the external food environment, primarily food availability and food prices. Compared to households with low food availability (< 4 food groups), high availability (8–10 food groups) is associated with a 12% increase in WDDS (exp(β) = 1.12, *p* < 0.01) and a 42% higher HDDS (exp(β) = 1.42, *p* < 0.001), suggesting that greater local food diversity enhances women’s and household dietary diversity. While high availability shows a trend toward an increased FVS, this association was not statistically significant. Medium availability (5–7 food groups) also demonstrates positive associations, with an increased FVS (exp(β) = 1.22, *p* < 0.05) and HDDS (exp(β) = 1.22, *p* < 0.001). According to the results, food availability did not significantly affect the GDQS.

In addition to food availability, food prices significantly influence dietary outcomes. Higher prices for vegetables are associated with notable decreases in WDDS (11%, *p* < 0.001), FVS (21%, *p* < 0.001), and GDQS (6%, *p* < 0.001). Fruit prices slightly reduce GDQS (2%, *p* < 0.01), while increased prices for pulses, nuts, and seeds are linked to a 6% decrease in WDDS (*p* < 0.05). Conversely, higher prices for grains, roots, and tubers are associated with increases in WDDS (9%, *p* < 0.001), FVS (28%, *p* < 0.001), and GDQS (5%, *p* < 0.001). Similarly, higher meat prices lead to small but significant increases in WDDS (1%, *p* < 0.001), FVS (4%, *p* < 0.001), and GDQS (1%, *p* < 0.001), and fruit prices also increase HDDS (3%, *p* < 0.01). These findings indicate that while greater availability enhances diversity, rising prices for vegetables, fruits, and pulses hinder dietary quality, particularly for women’s diet quality.

#### Personal food environment

Factors reflecting household and individual access and preferences also play a crucial role. Better convenience in food preparation is positively associated with higher scores in WDDS (exp(β) = 1.06, *p* < 0.05), FVS (exp(β) = 1.18, *p* < 0.01), and HDDS (exp(β) = 1.11, *p* < 0.001), indicating that ease of food preparation promotes a more diverse diet. The desirability score, which captures the frequency of consuming preferred foods, exhibits even stronger effects. Higher desirability scores are associated with increased WDDS (exp(β) = 1.14, *p* < 0.001), FVS (exp(β) = 1.31, *p* < 0.001), and GDQS (exp(β) = 1.09, *p* < 0.001), with the effect particularly pronounced for FVS. This suggests that diets more closely aligned with food preferences possess higher quality, especially regarding fruit and vegetable consumption. Household non-affordability (i.e., lower affordability) is linked to a lower FVS (exp(β) = 1.33, *p* < 0.01), indicating that the ability to afford food increases women’s fruit and vegetable intake. However, non-affordability is not significantly associated with HDDS (exp(β) = 1.01, *p* < 0.01).

#### Supporting Infrastructure

Access to supporting infrastructure, such as market proximity and road access, demonstrates modest yet significant associations with dietary outcomes. A greater distance to markets links to lower dietary diversity, as indicated by a 1% decrease in WDDS (exp(β) = 0.99, *p* < 0.05) and FVS (exp(β) = 0.99, *p* < 0.05) for every additional kilometer. Similarly, increased distance to roads correlates with lower FVS and HDDS. The type of road access also matters, as access to dry-weather roads (compared to no road within one hour) positively associates with WDDS (exp(β) = 1.12, *p* < 0.001) and FVS (exp(β) = 1.20, *p* < 0.001), highlighting the advantages of even basic infrastructure. While gravel or all-weather roads show a positive but somewhat weaker correlation with dietary diversity (FVS), the impact is not as conclusive. In contrast, proximity to bus stations and banks has a limited effect, with significant associations identified only for women’s dietary diversity score.

**Fig. 6:**
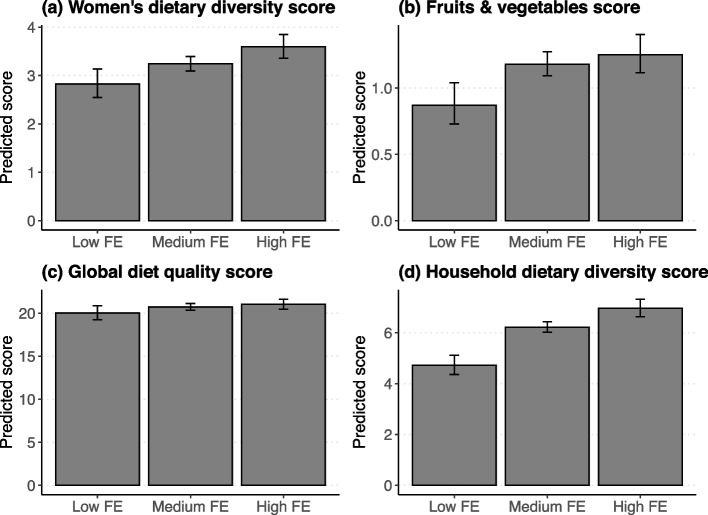
Predicted dietary scores across food environment (FE) categories. **a** Women’s dietary diversity score (WDDS), **b** Fruit and vegetable score (FVS), **c** Global diet quality score (GDQS), and **d** Household dietary diversity score (HDDS). Bars indicate predicted scores for low FE, medium FE, and high FE categories, with error bars representing confidence intervals

The overall food environment (FE) significantly influences dietary outcomes, with predicted dietary scores increasing across higher FE categories after adjusting for socioeconomic covariates (Fig. 6). Specifically, the Women’s Dietary Diversity Score (WDDS) and Fruit and Vegetable Score (FVS) are lowest in the Low FE category, with scores rising progressively in the Medium and High FE categories (Fig. 6a, 6b), indicating that a more favorable food environment enhances women’s dietary diversity and fruit and vegetable intake. Similarly, the Global Diet Quality Score (GDQS) and Household Dietary Diversity Score (HDDS) exhibit a clear upward trend across FE categories, with the highest scores observed in the High FE category (Fig. 6c, 6 d), underscoring the positive impact of improved food environments on overall dietary quality and household diversity. Detailed results for predicted dietary quality outcomes by FE category, adjusted for socioeconomic covariates, are available in Supplementary Figures S2–S3 (Additional file 3).

## Discussions

This study examined the influence of the food environment (FE) on multiple dietary quality outcomes among Ethiopian women and households, highlighting how FE dimensions—availability, accessibility, affordability, and infrastructure—influence dietary patterns and nutritional wellbeing [13, 16].

Our findings reveal distinct dietary consumption patterns among Ethiopian women across food environment (FE) categories, highlighting challenges in achieving diverse diets, with a mean Household Dietary Diversity Score (HDDS) of 6.24 out of 16 food groups indicating moderate diversity. A few women consumed nutrient-dense foods like fruits, vegetables, and animal-source foods, potentially contributing to insufficient micronutrient intake [67–69]. However, grains, white roots, and tubers were almost universally consumed, reflecting their role as dietary staples in Ethiopia [70, 71]. Intake of nutrient-rich food groups, such as pulses, fruits, and vegetables, modestly increased from low to high FE categories. In contrast, dairy consumption was notably higher in low FE households compared to medium/high FE households (Fig. 3), likely due to the cultural significance of dairy in agropastoral regions like Somalie and Oromia [72], where low FE scores reflect limited overall food diversity, market access, and affordability issues [73], yet traditional dairy practices persist [9]. These patterns underscore the influence of the food environment on dietary diversity, with factors such as availability, access, affordability, and cultural preferences likely shaping intake, which we explore further through socioeconomic and food environment predictors in the following sections.

The findings confirm the significant role of socioeconomic factors in predicting diet quality among women and households. Higher household income was associated with improved dietary outcomes, with each income decile increase leading to a small but significant rise in dietary scores, such as a 1% increase in Women Diet Diversity Score (WDDS) (exp(β) = 1.01, *p* < 0.05) and a 2% increase in Fruits and Vegetables Score (FVS) (exp(β) = 1.02, *p* < 0.1). This suggests that even modest income improvements can enhance access to more diverse and nutritious diets [74, 75]. Wealth disparities were particularly pronounced, with women in the poorest tertile experiencing significantly lower dietary diversity and quality across all indicators. For example, WDDS was 12% lower (exp(β) = 0.88, *p* < 0.01), and FVS was 14% lower (exp(β) = 0.86, *p* < 0.05) compared to the richest category. Similarly, Global Diet Quality Score (GDQS) and HDDS scores significantly declined, reflecting the impact of economic constraints on dietary patterns. These findings align with prior evidence that financial limitations restrict diverse and healthy food choices [75], limiting the ability to achieve an adequate diet in resource-poor settings [76]. The middle tertile showed intermediate effects, with significantly lower WDDS and HDDS compared to their richest counterparts, though some dietary indicators did not reach significance (Table 2). In contrast, women in the richest tertile consistently achieved higher dietary scores, reinforcing the advantages of economic stability in securing a diet quality [77]. These socioeconomic gradients emphasize the need to address financial barriers to improve dietary diversity in Ethiopia, particularly for the most disadvantaged households. Policies that enhance income opportunities and strengthen social safety nets could help mitigate the observed dietary inequalities.

The household head’s education had a statistically significant but practically small effect on diet quality, with each additional year of education associated with a 0–1% increase in the expected GDQS and HDDS. This modest effect aligns with prior research findings that correlated education with nutritional awareness and improved food choices [26, 78], but several factors may constrain its impact on our sample. First, the average education level attained by household heads in our sample was very low, with a mean of 6.1 years (SD = 0.12). This limited education may not translate into practical knowledge for improving dietary practices or unlocking other benefits tied to education, such as higher income, which could ultimately enhance diets [79]. Second, the food environment in Ethiopia, characterized by limited market access (e.g., mean distance to weekly markets = 6.7 km) and high non-affordability of diverse foods (74% of households reported non-affordability), may override the influence of education on diet quality [16]. Third, cultural influences, such as reliance on staple grains like teff and limited consumption of fruits and vegetables, may further diminish the role of education in diversifying diets [70]. Enhancing overall educational attainment may be advantageous, as evidence indicates that education can moderate the relationship between the food environment and dietary outcomes by improving the ability to overcome food access barriers [80].

Regional variations reveal how geography and socioeconomic conditions affect diet quality in Ethiopia. Compared to Oromia (the reference region), Amhara households exhibited notably lower average dietary scores: WDDS lower by 8% (exp(β) = 0.92, *p* < 0.05) and FVS lower by 38% (exp(β) = 0.62, *p* < 0.001). Somalie households also showed a substantially smaller WDDS (exp(β) = 0.70, *p* < 0.01). However, urban versus rural residence showed no meaningful difference, suggesting that regional factors outweigh urban–rural distinctions in this study. These results diverge from prior studies linking urban Ethiopia to greater dietary diversity [81], yet they resonate with evidence that region-specific food availability and income disparities drive nutritional outcomes [31, 82, 83]. The lower dietary scores in Amhara and Somalie may reflect limited food diversity, economic constraints, or cultural factors, underscoring the need to identify and address region-specific challenges to improve diet quality.

Food availability, assessed through market food diversity, significantly influences diet quality for Ethiopian women and households. Compared to low availability (< 4 groups), medium availability (5–7 groups) boosts women’s dietary diversity by 7% and household diversity by 22%, while high availability (8–10 groups) further enhances WDDS by 14% and HDDS by 42% (Table 2). This highlights how market availability of diverse foods can improve access to and consumption of nutrient-rich options, particularly when economic barriers are reduced, a crucial factor in contexts where dependence on staple foods limits micronutrient intake [70, 82, 83]. Previous studies have shown that markets help bridge nutritional gaps, enhancing dietary diversity within Ethiopia’s uneven food production landscape [83, 84]. However, market food availability did not significantly correlate with GDQS, suggesting that while diet diversity improved, it did not necessarily translate into adequate nutrient intake. This could be due to the low frequency of consumption [85], portion size limitations [86], or methodological constraints in dietary assessment [87], as discussed in the study’s limitations. Similarly, FVS exhibited a positive but non-significant trend with high market food availability, indicating that while market diversity may promote fruit and vegetable consumption, affordability and preference barriers may temper its impact [76].

The findings of this study reveal that food prices are crucial in determining diet quality, with impacts varying across food groups. Higher vegetable prices significantly reduced dietary quality, lowering the expected WDDS count by a factor of 0.89 (95% CI: 0.85–0.94, *p* < 0.001) and FVS by 0.79 (95% CI: 0.70–0.89, *p* < 0.001), reflecting greater price sensitivity for nutrient-rich foods compared to staples. This aligns with evidence that economic barriers disproportionately limit access to fruits and vegetables in low-income settings like Ethiopia [76], a pattern echoed by Ali [88], who found fruits and vegetables in Southwest Ethiopia to be price-elastic luxuries, with demand dropping sharply as prices rise. In contrast, rising prices of grains showed a slight increase in WDDS and FVS, possibly due to a substitution effect where expensive staples prompt shifts to cheaper, diverse alternatives, thereby enhancing dietary variety. Hirvonen [89] similarly notes that households adjust consumption toward available substitutes in response to price changes. These divergent impacts underscore the complex interplay between price and dietary choices, suggesting that stabilizing prices of nutritious foods such as fruits and vegetables could safeguard access to an adequate diversity of foods [82].

The study confirms that affordability is an important determinant of diet quality. Lower non-affordability (vs. high) increased FVS by a factor of 1.33 (95% CI: 1.08–1.65, *p* < 0.01), suggesting reduced financial strain improves fruit and vegetable intake, yet its non-significant effect on other indicators like WDDS or GDQS implies affordability alone may not suffice without addressing factors such as availability [22, 90]. While easing financial constraints enhances access to specific nutrient-rich foods, it does not consistently improve overall diet quality in resource-poor settings, where consumption patterns reflect competing priorities like cost and accessibility [13, 88]. These findings underscore the need for targeted interventions beyond price stabilization, such as subsidies for fruits and vegetables or improved market access, to ensure affordability translates into balanced, diverse diets for Ethiopian women and households [90, 91].

The personal dimensions of the food environment, particularly convenience and desirability, are significantly associated with diet quality. Higher convenience scores—reflecting time allocated for food preparation and domestic labor (often involving paid household workers for food-related tasks)—enhance dietary diversity, increasing FVS and HDDS. This indicates that reduced preparation burdens could encourage the inclusion of a variety of foods in diets [92]. Similarly, desirability, which is linked to preferred food consumption, notably improves WDDS and FVS, underscoring the impact of personal dynamics on nutrition. In this study, desirability reflects the more frequent consumption of foods households or individuals wish to eat. It significantly enhances women’s dietary diversity and fruit and vegetable intake, suggesting that nutritional gains occur when preferred foods are consumed more often [93]. This aligns with the idea that desirability serves as a motivator, encouraging healthier choices when access allows, despite economic and availability barriers [94].

Access to essential services influences diet quality in distinct ways. Greater distance from bus stations slightly reduces women’s dietary diversity, emphasizing the critical role of transportation, while the increased distance to markets modestly lowers fruit and vegetable consumption, reflecting market access challenges [82, 83]. Similarly, a greater distance to roads subtly hampers household dietary variety, underscoring the influence of infrastructure. Access to all-weather roads stands out, significantly boosting WDDS and FVS (Table 2). Prior studies confirm that enhanced road and market infrastructure improve access to nutritious foods. In contrast, no road access within an hour’s walk limits dietary outcomes, reinforcing that functional infrastructure reduces costs and enhances access to diverse, nutrient-rich foods, mitigating food insecurity in Ethiopia. [85, 92].

**Fig. 7:**
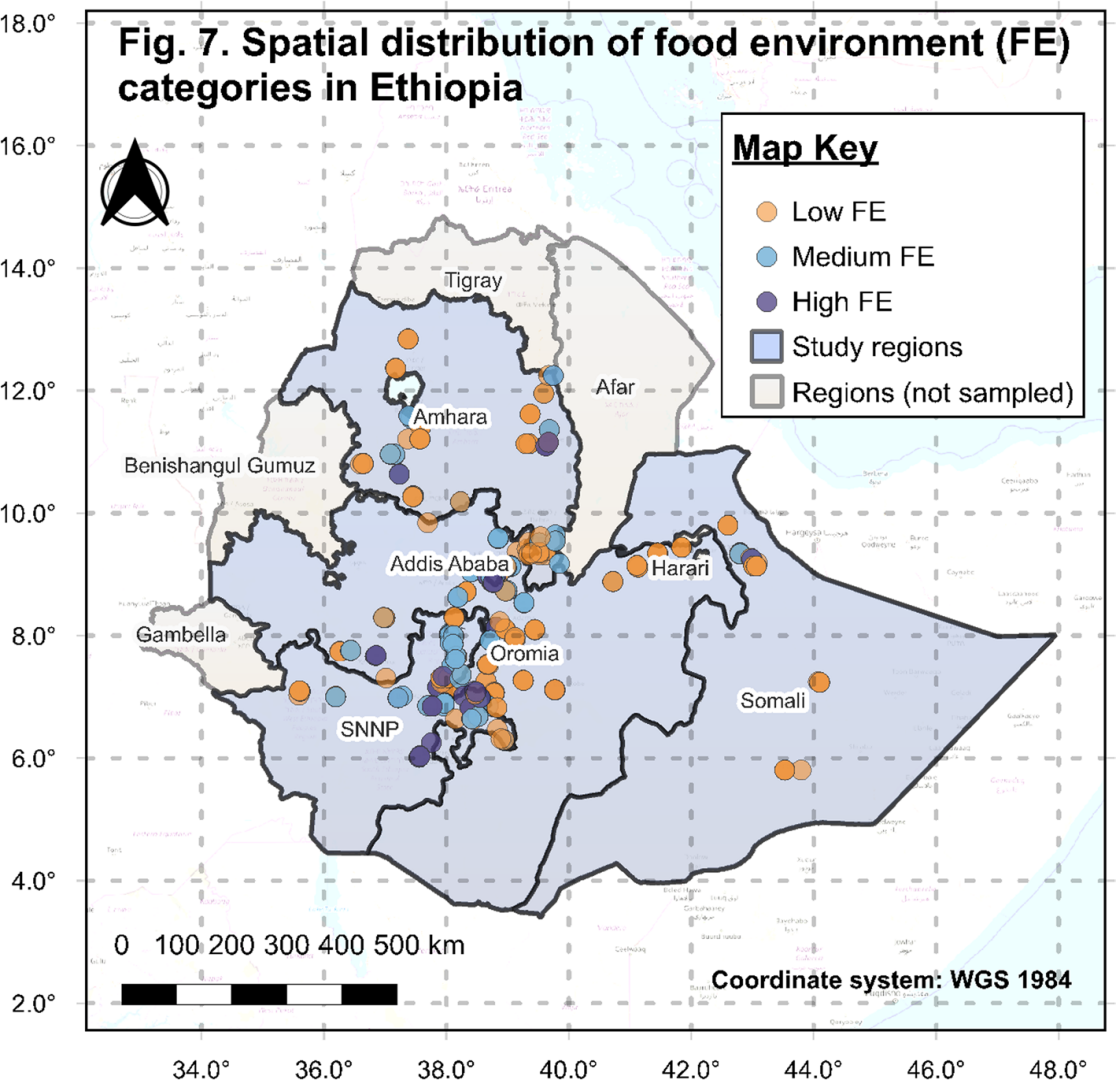
Spatial distribution of food environment categories across Ethiopia’s regions. Circle shapes represent households, with colors indicating food environment categories: Low, Medium, and High based on their FE score

Our analysis shows the impact of individual dimensions (external and personal) of the food environment in the previous sections. An alternative approach, examining their combined influence through the food environment score, demonstrates an even more substantial effect on all indicators. For instance, households with a high FE score see women’s dietary diversity increase by 27% compared to those with low scores (Fig. 6), underscoring that a comprehensive food environment amplifies nutritional outcomes beyond isolated factors [57]. These findings highlight the urgent need for integrated policies addressing Ethiopia’s uneven food environment landscapes to enhance diet quality equitably. The composite food environment score analysis reveals significant disparities in food environment performance across Ethiopia’s regions (Fig. 7). Although most households face a poor overall food environment, the central highlands exhibit relatively better FE, reinforcing socioeconomic, geographic, and infrastructural disparities [58]. In contrast, remote or lowland areas like Somalie face substantial barriers, as observed on the spatial map.

## Limitations

This study utilized a large survey dataset to examine household and individual diets within the food system framework, providing valuable insights while presenting notable limitations. The cross-sectional design restricts causal inference, and single-day dietary recall may not fully capture typical dietary patterns, necessitating context-specific validation of adapted food environment frameworks. These constraints require cautious interpretation of the findings. Future research should employ longitudinal designs, multi-day dietary assessments, and mixed methods to validate food environment indicators and explore systemic factors, thereby informing effective interventions to improve nutrition outcomes, particularly for vulnerable groups such as women of reproductive age.

## Conclusion

This study confirms the critical role of a comprehensive food environment (FE) in improving dietary quality among Ethiopian women and households. The FE encompasses physical access (e.g., diverse food availability), economic access (e.g., affordability and income), and supporting infrastructure (e.g., proximity to markets, transportation, roads, and financial services), all of which facilitate healthier dietary choices. However, persistent challenges, such as limited dietary diversity and inadequate consumption of nutrient-dense foods, are exacerbated by high food prices, which particularly restrict access to nutrient-rich foods. Women in low-income households exhibit markedly lower dietary scores, with socioeconomic disparities and regional variations (e.g., lower diversity in Amhara and Somali compared to Oromia) compounding these issues. Barriers, including poor market access and non-affordability, further limit access. To enhance nutrition and food security, policymakers should pursue a comprehensive approach. This includes strengthening market infrastructure and measures to increase the availability of diverse foods in markets, improving affordability through economic support and price stabilization initiatives, and implementing region-specific interventions to ensure equitable access to a variety of nutritious foods.

## Supplementary Information

Additional file 1: Supplementary tables: GDQS scoring methods and women’s 24-hour food group consumption  (Ethiopia, *N*=1828).

Additional file 2. Model fit and full Poisson regression results for dietary and food environment analysis.

Additional file 3. Supplementary figures on predicted diet quality scores by food environment among study samples.

Additional file 4. Questionnaire utilized to conduct the household survey.

Additional file 5: Table S1: Overview of additional files supporting the diet quality and food environment analysis.

## Data Availability

Data supporting the findings of this study are provided within the manuscript and supplementary information files. Additional datasets are available from the corresponding author upon reasonable request.

## Abbreviations

CAPI: Computer-Assisted Personal Interviewing
CPI: Consumer Price Index
DQQ: Diet Quality Questionnaire
ESS: Ethiopian Socioeconomic Survey
FFQ: Food Frequency Questionnaire
FAO: Food And Agriculture Organization
FE: Food Environment
Fig.: Figure
FVS: Fruits and Vegetables Score
GDQS: Global Dietary Quality Score
HDDS: Household Diet Diversity Score
HICES: Household Income and Expenditure Survey
LMICs: Low and Middle-Income Countries
PCA: Principal Component Analysis
MDD-W: Minimum Dietary Diversity for Women
NCD: Noncommunicable Diseases
SD: Standard Deviation
SES: Socioeconomic Status
SNNPR: Southern Nations, Nationalities, And Peoples
WDDS: Women’s Diet Diversity Score
