# Supplementary material for: The influence of the food environment on diet quality: Insights from an extensive household survey in Ethiopia, focusing on women of reproductive age
Source: BMC Nutr. 2025 Jun 2;11:107. doi: 10.1186/s40795-025-01097-z (PMC12128275; doi:10.1186/s40795-025-01097-z)
Supplement: Supplementary file 1 — Additional file 1: Supplementary tables: GDQS scoring methods and women’s 24-hour food group consumption (Ethiopia, N=1828). [file 40795_2025_1097_MOESM1_ESM.docx]

**Additional File 1:** Supplementary Tables: GDQS Scoring Methods and Women's 24-Hour Food Group Consumption (Ethiopia, N=1828)

Description: Supplementary tables on Global diet quality score (GDQS) scoring, intake, and distribution by food group in Ethiopia. Contains Table S2: GDQS scoring and mean intake; Table S3: Distribution of women across GDQS intake categories by food group.

- Table S2: GDQS scoring and mean intake by food group among women in Ethiopia.
- Table S3: Distribution of women across GDQS intake categories by food group.

Table S2: GDQS scoring and mean intake by food group among women in Ethiopia (N=1828)

| **Food Group** | **GDQS Scoring Ranges (g/day)** | **Sub-scores** | **Mean Intake (g/day)** | **SD** |
| --- | --- | --- | --- | --- |
| **Healthy Components** |  |  |  |  |
| Citrus fruits | <24/ 24–69/ >69 | 0 / 1 / 2 | 9.94 | 20.19 |
| Deep orange fruits | <25/ 25–123 />123 | 0 / 1 / 2 | 39.94 | 72.83 |
| Other fruits | <27/ 27–107/ >107 | 0 / 1 / 2 | 34.75 | 50.22 |
| Dark green leafy vegetables | <13/ 13–37/ >37 | 0 / 2 / 4 | 17.59 | 29.79 |
| Cruciferous vegetables | <13/ 13–36/ >36 | 0 / .25 / .5 | 0 | 0 |
| Deep orange vegetables | <9/ 9–45/ >45 | 0 / .25 / .5 | 32.54 | 51.65 |
| Other vegetables | <23/ 23–114/ >114 | 0 / 0.25 / 0.5 | 39.16 | 42.1 |
| Legumes | <9/9−42/>42 | 0 / 2 / 4 | 111.46 | 13.88 |
| Nuts and seeds | <7/7−13/>13 | 0 / 2 / 4 | 0.1 | .01 |
| Deep orange tubers | <12/ 12–63/ >63 | 0 / 0.25 / 0.5 | 32.54 | 51.65 |
| Whole grains | <8/ 8–13/ >13 | 0 / 1 / 2 | 454.66 | 213.97 |
| Liquid oils | <2/ 2–7.5/ >7.5 | 0 / 1 / 2 | 4.35 | 6.96 |
| Fish and shellfish | <14/ 14–71/ >71 | 0 / 1 / 2 | 2.57 | 8.14 |
| Poultry | <16/ 16–44 >44 | 0 / 1 / 2 | 8.22 | 19.16 |
| Low-fat dairy | <33/ 33–132/ >132 | 0 / 1 / 2 | 63.53 | 85.23 |
| Eggs | <6/ 6–32/ >32 | 0 / 1 / 2 | 10.89 | 15.52 |
| **Unhealthy in excessive amount** | | | |  |
| High-fat dairy | <35/ 35–142/>142-734/ >734 | 0 / 1 / 2/0 | 23.1 | 39.73 |
| Red meat | <9/ 9–46/ >46 | 0 / 1 / 0 | 14.74 | 25.1 |
| Processed meat | <9/9-30/>30 | 2/1/0 | NA | NA |
| Refined grains and baked goods | <7/7-33/>33 | 2/1/0 | 6.5 | 0.6 |
| Sweets and ice cream | <13/13-37/>37 | 2/1/0 | NA | NA |
| Sugar-sweetened beverages | <57/57−180/>180 | 2 / 1 / 0 | .156 | 0.05 |
| Juice | <36/36-144/>144 | 2/1/0 | 2.1 | 0.9 |
| White roots and tubers | <27/27−107/>107 | 2 / 1 / 0 | 26.49 | 59.27 |
| Purchased deep-fried foods | <9/9-45/>45 | 2 / 1 / 0 | 2.4 | 0.6 |
| **Notes:** GDQS scoring ranges separate low, medium, and high intake categories (e.g., <24 / 24–69 / >69). Sub-scores indicate points assigned to each category. NA: means no consumption was recorded or very small amount | | | | |

Table S3: Distribution of women across GDQS intake categories by food group (N=1828)

| **Food Group** | **% Women by GDQS Categories** |
| --- | --- |
| **Healthy Components** |  |
| Citrus fruits | 84.0/8.4/7.6 |
| Deep orange fruits | 50.6/41.1/8.4 |
| Other fruits | 64.1/24.7/11.2 |
| Dark green leafy vegetables | 35.9/43.4/20.7 |
| Cruciferous vegetables | 100/-/- |
| Deep orange vegetables | 45.2/33.9/20.1 |
| Other vegetables | 46.4/43.8/9.9 |
| Legumes | 10.7/27.6/61.7 |
| Nuts and seeds | 100/-/- |
| Deep orange tubers | 45.2/33.9/20.9 |
| Whole grains | 0.2/0.1/99.7 |
| Liquid oils | 68.8/18.3/12.9 |
| Fish and shellfish | 91.2/7.0/1.8 |
| Poultry | 88.8/5.5/5.7 |
| Low-fat dairy | 58.4/ 19.9/ 21.7 |
| Eggs | 66.0/ 25.0/9.0 |
| **Unhealthy in excessive amount** |  |
| High-fat dairy | 80.1/16.1/2.5/1.3 |
| Red meat | 30.9 / 64.0 / 5.1 |
| Processed meat | 100 / - / - |
| Refined grains and baked goods | 100 / - / - |
| Sweets and ice cream | 100 / - / - |
| Sugar-sweetened beverages | 100 / - / - |
| Juice | 100 / - / - |
| White roots and tubers | 82.8 / 8.8 / 8.4 |
| Purchased deep-fried foods | 100 / - / - |
| A dash (-) indicates no women in that category. | |
