# Supplementary material for: The influence of the food environment on diet quality: Insights from an extensive household survey in Ethiopia, focusing on women of reproductive age
Source: BMC Nutr. 2025 Jun 2;11:107. doi: 10.1186/s40795-025-01097-z (PMC12128275; doi:10.1186/s40795-025-01097-z)
Supplement: Supplementary file 2 — Additional file 2. Model fit and full Poisson regression results for dietary and food environment analysis. [file 40795_2025_1097_MOESM2_ESM.docx]

Additional File 2: Model fit and full Poisson regression results for diet quality and food environment analysis, Ethiopia

Description

This file contains model fit and regression results for the dietary and food environment analysis in Ethiopia. It includes: -

- Table S4: Poisson regression results by dietary quality metrics.
- Table S5: Overdispersion test for the Poisson full model (Model 3).
- Table S6: Detailed model fit statistics, including log pseudolikelihood and Wald chi-square.

Note: The maximum sample size is N=1828, but sample sizes vary across models and outcomes due to missing data (see Table S4 for details).

Table S4: Poisson Regression Analysis Results of Associations Between Dietary Quality, Socioeconomic Factors, and Food Environment Components.

|  | (1) | (2) | (3) | (1) | (2) | (3) | (1) | (2) | (3) | (1) | (2) | (3) |
| --- | --- | --- | --- | --- | --- | --- | --- | --- | --- | --- | --- | --- |
| **Variables** | WDDS (exp(β)– 95% CI) | WDDS (exp(β)– 95% CI) | WDDS (exp(β)– 95% CI) | FVS (exp(β)– 95% CI) | FVS (exp(β)– 95% CI) | FVS (exp(β)– 95% CI) | GDQS (exp(β)– 95% CI) | GDQS (exp(β)– 95% CI) | GDQS (exp(β)– 95% CI) | HDDS (exp(β)– 95% CI) | HDDS (exp(β)– 95% CI) | HDDS (exp(β)– 95% CI) |
| **Residence** |  |  |  |  |  |  |  |  |  |  |  |  |
| Rural | ref |  | ref | ref |  | ref | ref |  | ref | ref |  | ref |
| Urban | 1.10^**^ |  | 1.03 | 1.11 |  | 1.12 | 1.01 |  | 1.01 | 1.13^***^ |  | 0.98 |
|  | [1.04–1.17] |  | [0.97–1.11] | [0.99–1.24] |  | [0.97–1.28] | [0.98–1.04] |  | [0.97–1.04] | [1.08–1.18] |  | [0.93–1.02] |
| **Gender of HH head** |  |  |  |  |  |  |  |  |  |  |  |  |
| Female | ref |  | ref | ref |  | ref | ref |  | ref | ref |  | ref |
| Male | 0.98 |  | 0.99 | 0.97 |  | 1.02 | 1.00 |  | 1.01 | 0.99 |  | 0.99 |
|  | [0.93–1.03] |  | [0.94–1.04] | [0.86–1.10] |  | [0.90–1.14] | [0.97–1.03] |  | [0.98–1.04] | [0.95–1.03] |  | [0.96–1.03] |
| **Age of HH head** | 1.00^**^ |  | 1.00^**^ | 1.00^*^ |  | 1.00^*^ | 1.00 |  | 1.00 | 1.00 |  | 1.00 |
|  | [1.00–1.00] |  | [1.00–1.00] | [0.99–1.00] |  | [0.99–1.00] | [1.00–1.00] |  | [1.00–1.00] | [1.00–1.00] |  | [1.00–1.00] |
| **Head education (years)** | 1.01^***^ |  | 1.00 | 1.01^**^ |  | 1.00 | 1.01^***^ |  | 1.00^*^ | 1.01^***^ |  | 1.00^*^ |
|  | [1.00–1.01] |  | [1.00–1.01] | [1.00–1.02] |  | [1.00–1.01] | [1.00–1.01] |  | [1.00–1.01] | [1.01–1.01] |  | [1.00–1.01] |
| **Regions** |  |  |  |  |  |  |  |  |  |  |  |  |
| Oromia | ref |  | ref | ref |  | ref | ref |  | ref | ref |  | ref |
| Amhara | 0.80^***^ |  | 0.92^*^ | 0.44^***^ |  | 0.62^***^ | 0.89^***^ |  | 1.00 | 0.83^***^ |  | 0.83^***^ |
|  | [0.76–0.84] |  | [0.85–0.99] | [0.38–0.52] |  | [0.50–0.76] | [0.86–0.92] |  | [0.96–1.05] | [0.80–0.87] |  | [0.78–0.88] |
| Somalie | 0.73^***^ |  | 0.70^**^ | 0.81 |  | 0.63 | 0.77^***^ |  | 0.95 | 0.99 |  | 0.88 |
|  | [0.63–0.85] |  | [0.54–0.90] | [0.61–1.06] |  | [0.38–1.04] | [0.71–0.85] |  | [0.82–1.10] | [0.90–1.09] |  | [0.71–1.08] |
| SNNP | 0.90^**^ |  | 0.95 | 1.05 |  | 1.20 | 0.99 |  | 1.05^*^ | 0.85^***^ |  | 0.79^***^ |
|  | [0.84–0.96] |  | [0.87–1.03] | [0.94–1.18] |  | [1.00–1.43] | [0.96–1.03] |  | [1.00–1.11] | [0.80–0.90] |  | [0.74–0.84] |
| Sidama | 0.83^***^ |  | 0.92 | 0.85 |  | 0.64^*^ | 0.91^***^ |  | 0.90^*^ | 0.89^**^ |  | 0.93 |
|  | [0.76–0.90] |  | [0.78–1.09] | [0.71–1.01] |  | [0.44–0.94] | [0.86–0.95] |  | [0.82–0.99] | [0.83–0.96] |  | [0.82–1.06] |
| **Wealth status** |  |  |  |  |  |  |  |  |  |  |  |  |
| Poorest | 0.86^***^ |  | 0.88^***^ | 0.80^***^ |  | 0.86^**^ | 0.95^***^ |  | 0.97^*^ | 0.96^*^ |  | 0.93^***^ |
|  | [0.82–0.90] |  | [0.83–0.92] | [0.72–0.88] |  | [0.77–0.96] | [0.92–0.97] |  | [0.94–1.00] | [0.92–0.99] |  | [0.90–0.96] |
| Middle | 0.91^***^ |  | 0.92^***^ | 0.88^*^ |  | 0.91 | 0.97^*^ |  | 0.98 | 0.94^**^ |  | 0.95^**^ |
|  | [0.87–0.95] |  | [0.88–0.97] | [0.80–0.98] |  | [0.82–1.01] | [0.94–0.99] |  | [0.95–1.01] | [0.91–0.98] |  | [0.92–0.98] |
| Richest | ref |  | ref | ref |  | ref | ref |  | ref | ref |  | ref |
| **Monthly income decile (ETB)** | 1.03^***^ |  | 1.01^**^ | 1.07^***^ |  | 1.02^*^ | 1.03^***^ |  | 1.01^***^ | 1.02^***^ |  | 1.01 |
|  | [1.03–1.04] |  | [1.00–1.02] | [1.05–1.08] |  | [1.00–1.04] | [1.02–1.03] |  | [1.01–1.02] | [1.01–1.02] |  | [1.00–1.01] |
| **External Domain** |  |  |  |  |  |  |  |  |  |  |  |  |
| **Food availability** |  |  |  |  |  |  |  |  |  |  |  |  |
| Low (<4 FGs) |  | ref | ref |  | ref | ref |  | ref | ref |  | ref | ref |
| Medium (5-7 FGs) |  | 1.05 | 1.07 |  | 1.23^*^ | 1.22^*^ |  | 0.95^*^ | 0.95^*^ |  | 1.19^***^ | 1.22^***^ |
|  |  | [0.98–1.13] | [1.00–1.15] |  | [1.03–1.46] | [1.01–1.46] |  | [0.90–1.00] | [0.90–1.00] |  | [1.12–1.26] | [1.15–1.30] |
| High (8-10 FGs) |  | 1.10^**^ | 1.12^**^ |  | 1.27^**^ | 1.18 |  | 1.0 | 1.01** |  | 1.37^***^ | 1.42^***^ |
|  |  | [1.04–1.17] | [1.04–1.18] |  | [1.09–1.50] | [0.99–1.41] |  | [0.90–1.01] | [0.88–1.01] |  | [1.30–1.44] | [1.35–1.50] |
| **Price** |  |  |  |  |  |  |  |  |  |  |  |  |
| Grain, root and tubers |  | 1.10^***^ | 1.09^***^ |  | 1.32^***^ | 1.28^***^ |  | 1.06^***^ | 1.05^***^ |  | 1.02^*^ | 0.99 |
|  |  | [1.07–1.13] | [1.05–1.13] |  | [1.24–1.41] | [1.17–1.39] |  | [1.04–1.08] | [1.03–1.08] |  | [1.00–1.04] | [0.96–1.02] |
| Vegetables |  | 0.88^***^ | 0.89^***^ |  | 0.73^***^ | 0.79^***^ |  | 0.94^***^ | 0.94^***^ |  | 0.96^**^ | 1.00 |
|  |  | [0.84–0.91] | [0.85–0.94] |  | [0.66–0.80] | [0.70–0.89] |  | [0.92–0.97] | [0.91–0.97] |  | [0.93–0.99] | [0.96–1.04] |
| Fruits |  | 0.97^**^ | 1.00 |  | 0.92^***^ | 1.02 |  | 0.97^***^ | 0.98^**^ |  | 1.02^*^ | 1.03^**^ |
|  |  | [0.95–0.99] | [0.97–1.02] |  | [0.88–0.96] | [0.96–1.09] |  | [0.96–0.98] | [0.96–0.99] |  | [1.00–1.03] | [1.01–1.05] |
| Meats, fish and poultry |  | 1.02^***^ | 1.01^***^ |  | 1.05^***^ | 1.04^***^ |  | 1.01^***^ | 1.01^***^ |  | 1.01^***^ | 1.00 |
|  |  | [1.02–1.02] | [1.01–1.02] |  | [1.04–1.06] | [1.03–1.06] |  | [1.01–1.01] | [1.01–1.01] |  | [1.01–1.02] | [1.00–1.01] |
| Milk |  | 1.01^***^ | 1.00^*^ |  | 1.00^*^ | 1.00 |  | 1.00^**^ | 1.00 |  | 1.00^**^ | 1.00 |
|  |  | [1.00–1.01] | [1.00–1.01] |  | [1.00–1.01] | [0.99–1.00] |  | [1.00–1.00] | [1.00–1.00] |  | [1.00–1.00] | [1.00–1.00] |
| Pulses– nuts– and seeds |  | 0.92^***^ | 0.94^*^ |  | 0.84^***^ | 0.98 |  | 1.00 | 1.02 |  | 1.00 | 0.99 |
|  |  | [0.88–0.95] | [0.89–1.00] |  | [0.77–0.92] | [0.85–1.13] |  | [0.98–1.02] | [0.99–1.05] |  | [0.97–1.02] | [0.95–1.03] |
| Eggs |  | 1.00 | 1.02^*^ |  | 1.03^*^ | 1.02 |  | 1.00 | 1.00 |  | 1.00 | 1.00 |
|  |  | [0.99–1.01] | [1.00–1.03] |  | [1.00–1.05] | [0.99–1.06] |  | [1.00–1.01] | [0.99–1.01] |  | [0.99–1.01] | [0.99–1.02] |
| **Personal Domain** |  |  |  |  |  |  |  |  |  |  |  |  |
| **Expenditure decile [ETB]** |  | 1.02^***^ | 1.01^**^ |  | 1.02^*^ | 1.00 |  | 1.01^***^ | 1.01^*^ |  | 1.02^***^ | 1.02^***^ |
|  |  | [1.01–1.03] | [1.00–1.02] |  | [1.00–1.04] | [0.98–1.02] |  | [1.01–1.02] | [1.00–1.01] |  | [1.02–1.03] | [1.01–1.03] |
| **HH’s non-affordability [NA]** |  |  |  |  |  |  |  |  |  |  |  |  |
| High NA |  | ref | ref |  | ref | ref |  | ref | ref |  | ref | ref |
| Medium NA |  | 1.07^*^ | 1.03 |  | 1.38^***^ | 1.17^*^ |  | 1.03^*^ | 1.01 |  | 0.98 | 0.97 |
|  |  | [1.01–1.13] | [0.97–1.10] |  | [1.21–1.59] | [1.01–1.35] |  | [1.00–1.07] | [0.98–1.05] |  | [0.93–1.02] | [0.92–1.02] |
| Low NA |  | 1.09^**^ | 1.01 |  | 1.64^***^ | 1.33^**^ |  | 1.05^**^ | 1.04 |  | 0.93^**^ | 0.88^**^ |
|  |  | [1.03–1.15] | [0.92–1.11] |  | [1.43–1.88] | [1.08–1.65] |  | [1.01–1.09] | [0.98–1.09] |  | [0.89–0.97] | [0.81–0.95] |
| **Distance to market–km** |  | 1.00^*^ | 1.00^*^ |  | 0.98^**^ | 0.99^*^ |  | 1.00^***^ | 1.00^**^ |  | 1.00 | 1.00 |
|  |  | [0.99–1.00] | [0.99–1.00] |  | [0.97–0.99] | [0.98–1.00] |  | [0.99–1.00] | [0.99–1.00] |  | [1.00–1.00] | [1.00–1.00] |
| **Distance to road– km** |  | 1.00^*^ | 1.00 |  | 1.00^*^ | 0.99^**^ |  | 1.00^***^ | 1.00^***^ |  | 1.00^***^ | 1.00^***^ |
|  |  | [1.00–1.00] | [1.00–1.00] |  | [0.99–1.00] | [0.99–1.00] |  | [1.00–1.00] | [1.00–1.00] |  | [1.00–1.00] | [1.00–1.00] |
| **Distance to bus station–km** |  | 1.00^***^ | 1.00^**^ |  | 1.00 | 1.00 |  | 1.00 | 1.00 |  | 1.00^*^ | 1.00 |
|  |  | [0.99–1.00] | [0.99–1.00] |  | [0.99–1.00] | [0.99–1.00] |  | [1.00–1.00] | [1.00–1.00] |  | [1.00–1.00] | [1.00–1.00] |
| **Distance to bank–km** |  | 1.00^*^ | 1.00 |  | 1.01 | 1.01^**^ |  | 1.00 | 1.00 |  | 1.00 | 1.00 |
|  |  | [1.00–1.01] | [1.00–1.01] |  | [1.00–1.01] | [1.00–1.02] |  | [1.00–1.00] | [1.00–1.00] |  | [1.00–1.00] | [1.00–1.00] |
| **Type of road access** |  |  |  |  |  |  |  |  |  |  |  |  |
| No road with in 1 hour |  | ref | ref |  | ref | ref |  | ref | ref |  | ref | ref |
| Dry weather road |  | 1.02 | 1.04 |  | 0.97 | 1.06 |  | 1.00 | 1.00 |  | 0.99 | 1.02 |
|  |  | [0.97–1.07] | [0.99–1.10] |  | [0.87–1.09] | [0.95–1.19] |  | [0.98–1.03] | [0.97–1.02] |  | [0.95–1.02] | [0.98–1.05] |
| Gravel/all-weather |  | 1.09^***^ | 1.12^***^ |  | 1.17^**^ | 1.20^***^ |  | 1.03 | 1.01 |  | 1.00 | 1.03 |
|  |  | [1.04–1.15] | [1.07–1.18] |  | [1.05–1.31] | [1.08–1.34] |  | [0.99–1.06] | [0.98–1.04] |  | [0.97–1.04] | [0.99–1.07] |
| **Convenience score** |  | 1.09^**^ | 1.06^*^ |  | 1.31^***^ | 1.18^**^ |  | 1.04^*^ | 1.02 |  | 1.14^***^ | 1.11^***^ |
|  |  | [1.03–1.14] | [1.00–1.11] |  | [1.18–1.46] | [1.06–1.32] |  | [1.01–1.08] | [0.99–1.06] |  | [1.09–1.18] | [1.06–1.15] |
| **Desirability score** |  | 1.20^***^ | 1.14^***^ |  | 1.39^***^ | 1.31^***^ |  | 1.14^***^ | 1.09^***^ |  | 1.05^**^ | 1.03 |
|  |  | [1.15–1.25] | [1.10–1.19] |  | [1.27–1.52] | [1.19–1.45] |  | [1.11–1.17] | [1.07–1.12] |  | [1.02–1.08] | [1.00–1.07] |
| **Prob > chi2** | <0.001 | <0.001 | <0.001 | <0.001 | <0.001 | <0.001 | <0.001 | <0.001 | <0.001 | <0.001 | <0.001 | <0.001 |
| **Pseudo R2** | 0.0340 | 0.0396 | 0.0468 | 0.0961 | 0.0956 | 0.1256 | 0.0423 | 0.0485 | 0.0598 | 0.0314 | 0.0481 | 0.0562 |
| ***N*** | 1828 | 1818 | 1818 | 1826 | 1816 | 1816 | 1828 | 1818 | 1818 | 1828 | 1818 | 1818 |
| **Notes**: Results are presented as incidence rate ratios (exp(β)) with 95% confidence intervals. Model 1 includes sociodemographic covariates only (residence, gender of household head, age of household head, head education, regions, wealth status, monthly income decile). Model 2 adds external food environment (FE) components (food availability, price). Model 3 adds personal FE components (expenditure decile, household non-affordability, distance to market/road/bus station/bank, type of road access, convenience score, desirability score). Significance levels: *p<0.05, **p<0.01, ***p<0.001. Sample sizes vary due to missing data for certain variables or outcomes. WDDS: Women’s Dietary Diversity Score; FVS: Fruits and Vegetables Score; GDQS: Global Diet Quality Score; HDDS: Household Dietary Diversity Score. | | | | | | | | | | | | |

Table S5: Overdispersion Test for Poisson Full Model (Model 3)

|  | Dispersion |
| --- | --- |
| WDDS | 1.0000 |
| FVS | 1.0000 |
| GDQS | 1.0000 |
| HDDS | 1.0000 |
| **Notes**: Dispersion = Pearson chi2 / df. Values > 1 suggest overdispersion, supporting potential use of negative binomial regression. If ≈ 1, Poisson is adequate. WDDS: Women’s Dietary Diversity Score; FVS: Fruits and Vegetables Score; GDQS: Global Diet Quality Score; HDDS: Household Dietary Diversity Score. | |

Table S6: Detailed Model Fit Statistics for Poisson Regression Models

| Metric | DDS  (Model 3) | FVS  (Model 3) | GDQS (Model 3) | HDDS (Model 3) | DDS  (Model 4) | FVS  (Model 4) | GDQS (Model 4) | HDDS (Model 4) |
| --- | --- | --- | --- | --- | --- | --- | --- | --- |
| Log Pseudolikelihood | -3143.485 | -2452.880 | -5269.025 | -3751.011 | -3194.304 | -2540.474 | -5389.131 | -3840.299 |
| Wald Chi-Square | 845.549 | 767.687 | 954.310 | 1157.633 | 511.545 | 526.893 | 497.129 | 644.511 |
| P-Value | <0.001 | <0.001 | <0.001 | <0.001 | <0.001 | <0.001 | <0.001 | <0.001 |
| Degrees of Freedom | 37 | 37 | 37 | 37 | 17 | 17 | 17 | 17 |
| AIC | 6362.970 | 4981.760 | 10614.050 | 7578.022 | 6424.609 | 5116.949 | 10814.262 | 7716.597 |
| BIC | 6572.387 | 5191.177 | 10823.467 | 7787.439 | 6523.806 | 5216.146 | 10913.459 | 7815.795 |
| **Notes**: Model 3 includes sociodemographic covariates, external FE, and personal FE. Model 4 includes FE composite score and sociodemographic covariates. AIC: Akaike Information Criterion; BIC: Bayesian Information Criterion. Wald Chi-Square tests the overall significance of the model. P-values less than 0.001 are reported as <0.001. Sample sizes vary by model and outcome due to missing data (see Table S5 for details). | | | | | | | | |
