# Supplementary material for: The influence of the food environment on diet quality: Insights from an extensive household survey in Ethiopia, focusing on women of reproductive age
Source: BMC Nutr. 2025 Jun 2;11:107. doi: 10.1186/s40795-025-01097-z (PMC12128275; doi:10.1186/s40795-025-01097-z)
Supplement: Supplementary file 3 — Additional file 3. Supplementary figures on predicted diet quality scores by food environment among study samples. [file 40795_2025_1097_MOESM3_ESM.docx]

Additional File 3: Supplementary figures on predicted dietary scores by food environment (FE) among study samples (n=1828)

Description: This file contains supplementary figures on predicted dietary scores by food environment (FE) among study samples (N=1826). It includes:

- Figure S1: Normalized predicted dietary outcomes (WDDS, FVS, and GDQS) by FE categories.

- Figure S2: Predicted WDDS by FE score and wealth tertile.

- Figure S3: Predicted FVS by FE score and wealth tertile.

- Figure S4: Predicted GDQS by FE score and wealth tertile.

- Figure S5: Predicted HDDS by FE score and wealth tertile.

Note: HDDS is not included in Figure S1 due to incomplete data for some FE categories but is presented separately in Figure S5.

Figure S1: Normalized Predicted Dietary Outcomes (WDDS, FVS, and GDQS) by FE Categories

The plot shows the normalized predicted scores (0–1) for three dietary outcomes across food environment (FE) categories (Low FE, Medium FE, High FE), adjusted for sociodemographic covariates including wealth tertiles, sex of household head, and age of household head. Models used: linear regression for all outcomes. Marginal effects were computed by predicting scores at the mean values of continuous covariates and reference levels of categorical covariates. Points represent predicted scores, error bars represent 95% confidence intervals, and dashed lines connect the points for each outcome. The y-axis is scaled from 0.1 to 0.5 to highlight differences between FE categories. Outcomes are color-coded in the legend: Fruits and Vegetables Score (FVS), Global Diet Quality Score (GDQS), and Women’s Dietary Diversity Score (WDDS).

Figure S2: Predicted WDDS by FE Score and Wealth Tertile

The plot shows the predicted Women’s Dietary Diversity Score (WDDS, in counts) across food environment (FE) categories (Low FE, Medium FE, High FE) and wealth tertiles (Poorest, Middle, Richest), adjusted for sociodemographic covariates including sex of household head and age of household head. Models used: Poisson regression. Marginal effects were computed by predicting scores at the mean values of continuous covariates and reference levels of categorical covariates, with FE and wealth tertile as interacting factors. Points represent predicted scores, error bars represent 95% confidence intervals, and dashed lines connect the points for each wealth tertile. Outcomes are color-coded in the legend by wealth tertile.

Figure S3: Predicted FVS by FE Score and Wealth Tertile

The plot shows the predicted Fruits and Vegetables Score (FVS, in counts) across food environment (FE) categories (Low FE, Medium FE, High FE) and wealth tertiles (Poorest, Middle, Richest), adjusted for sociodemographic covariates including sex of household head and age of household head. Models used: Poisson regression. Marginal effects were computed by predicting scores at the mean values of continuous covariates and reference levels of categorical covariates, with FE and wealth tertile as interacting factors. A small constant (0.5) was added to FVS to handle excess zeros. Points represent predicted scores, error bars represent 95% confidence intervals, and dashed lines connect the points for each wealth tertile. Outcomes are color-coded in the legend by wealth tertile.

Figure S4: Predicted GDQS by FE Score and Wealth Tertile

The plot shows the predicted Global Diet Quality Score (GDQS, in counts) across food environment (FE) categories (Low FE, Medium FE, High FE) and wealth tertiles (Poorest, Middle, Richest), adjusted for sociodemographic covariates including sex of household head and age of household head. Models used: Poisson regression. Marginal effects were computed by predicting scores at the mean values of continuous covariates and reference levels of categorical covariates, with FE and wealth tertile as interacting factors. Points represent predicted scores, error bars represent 95% confidence intervals, and dashed lines connect the points for each wealth tertile. Outcomes are color-coded in the legend by wealth tertile.

Figure S5: Predicted HDDS by FE Score and Wealth Tertile

The plot shows the predicted Household Dietary Diversity Score (HDDS, in counts) across food environment (FE) categories (Low FE, Medium FE, High FE) and wealth tertiles (Poorest, Middle, Richest), adjusted for sociodemographic covariates including sex of household head and age of household head. Models used: Poisson regression. Marginal effects were computed by predicting scores at the mean values of continuous covariates and reference levels of categorical covariates, with FE and wealth tertile as interacting factors. Points represent predicted scores, error bars represent 95% confidence intervals, and dashed lines connect the points for each wealth tertile. Outcomes are color-coded in the legend by wealth tertile.


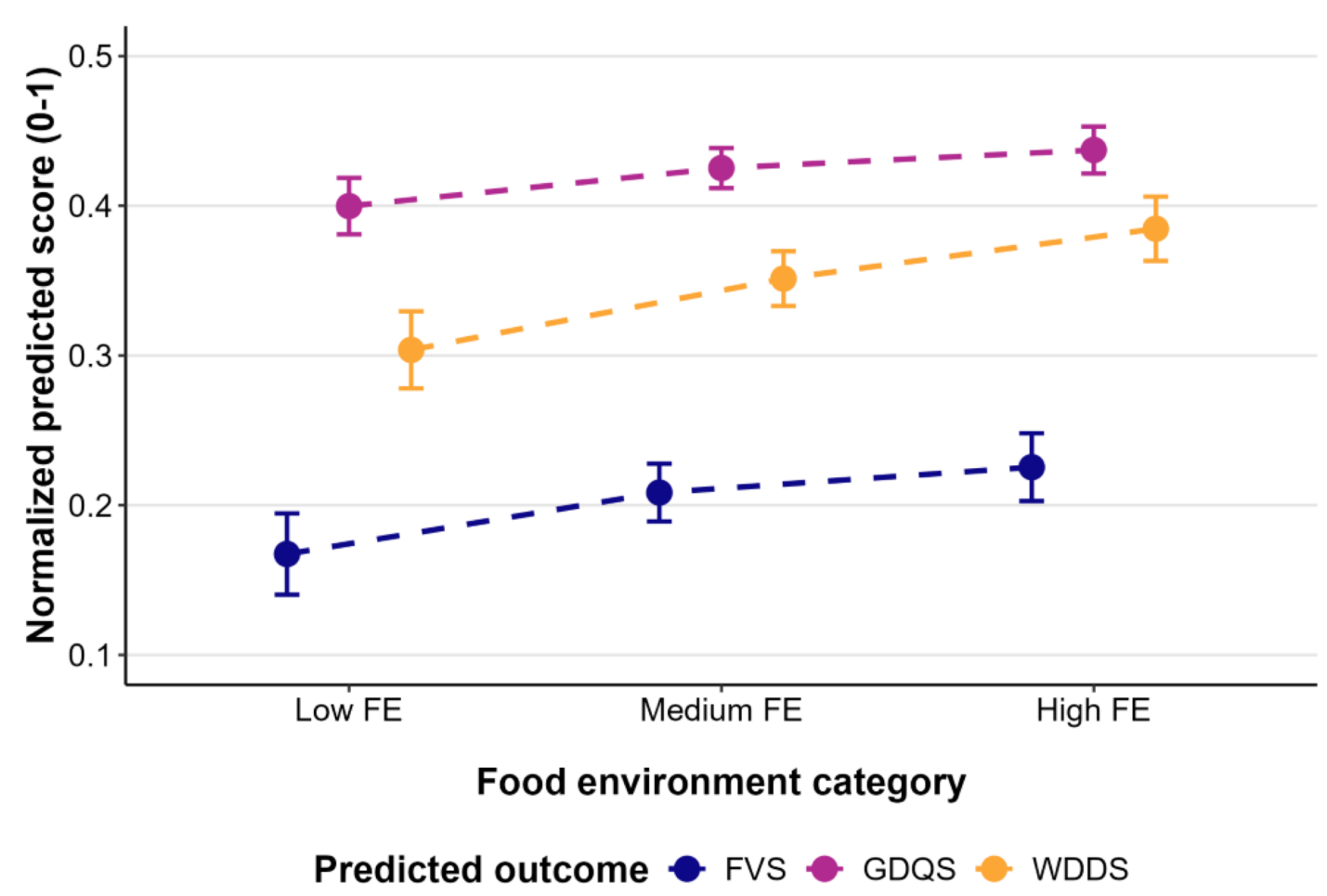
Figure S1


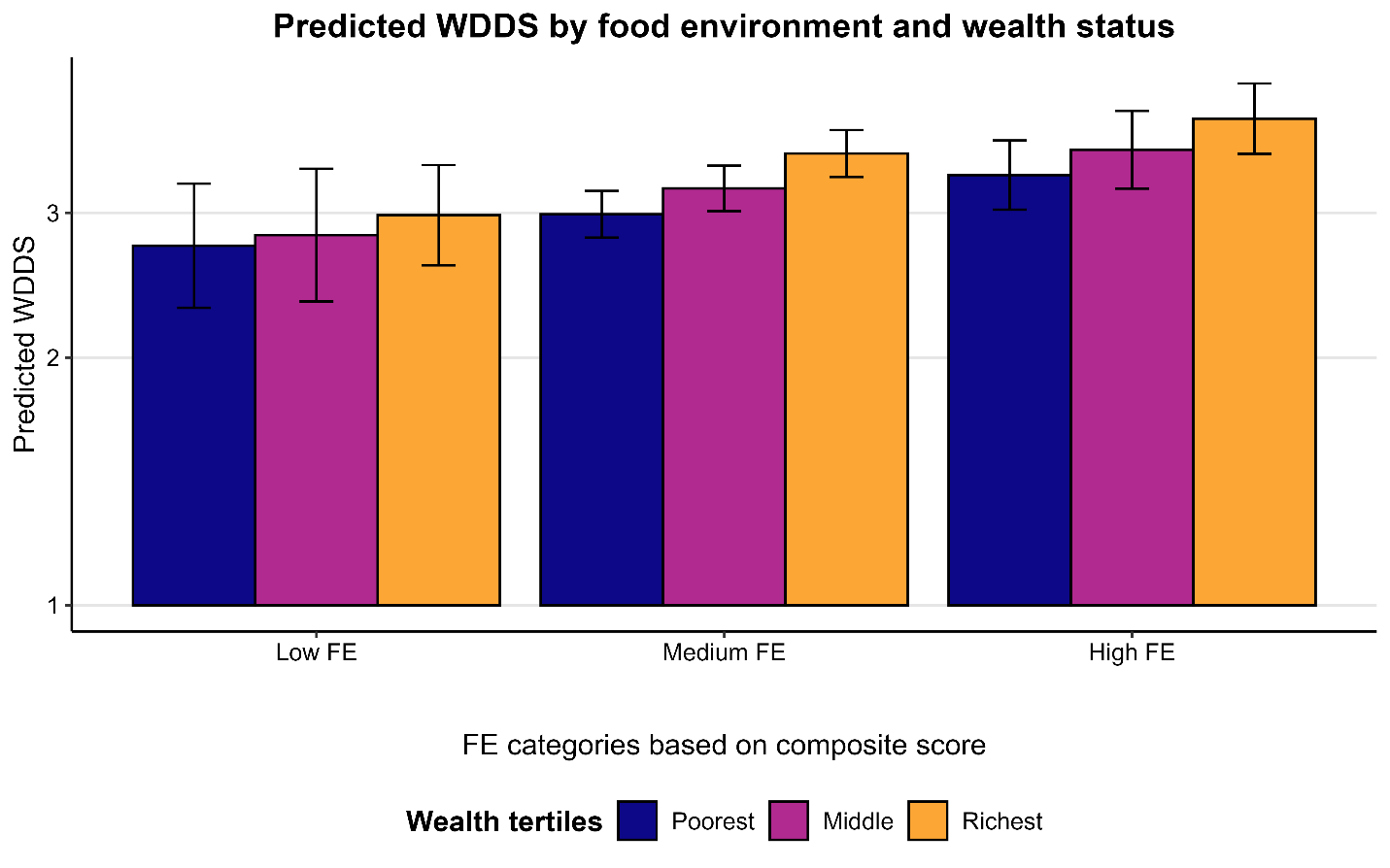
Figure S2


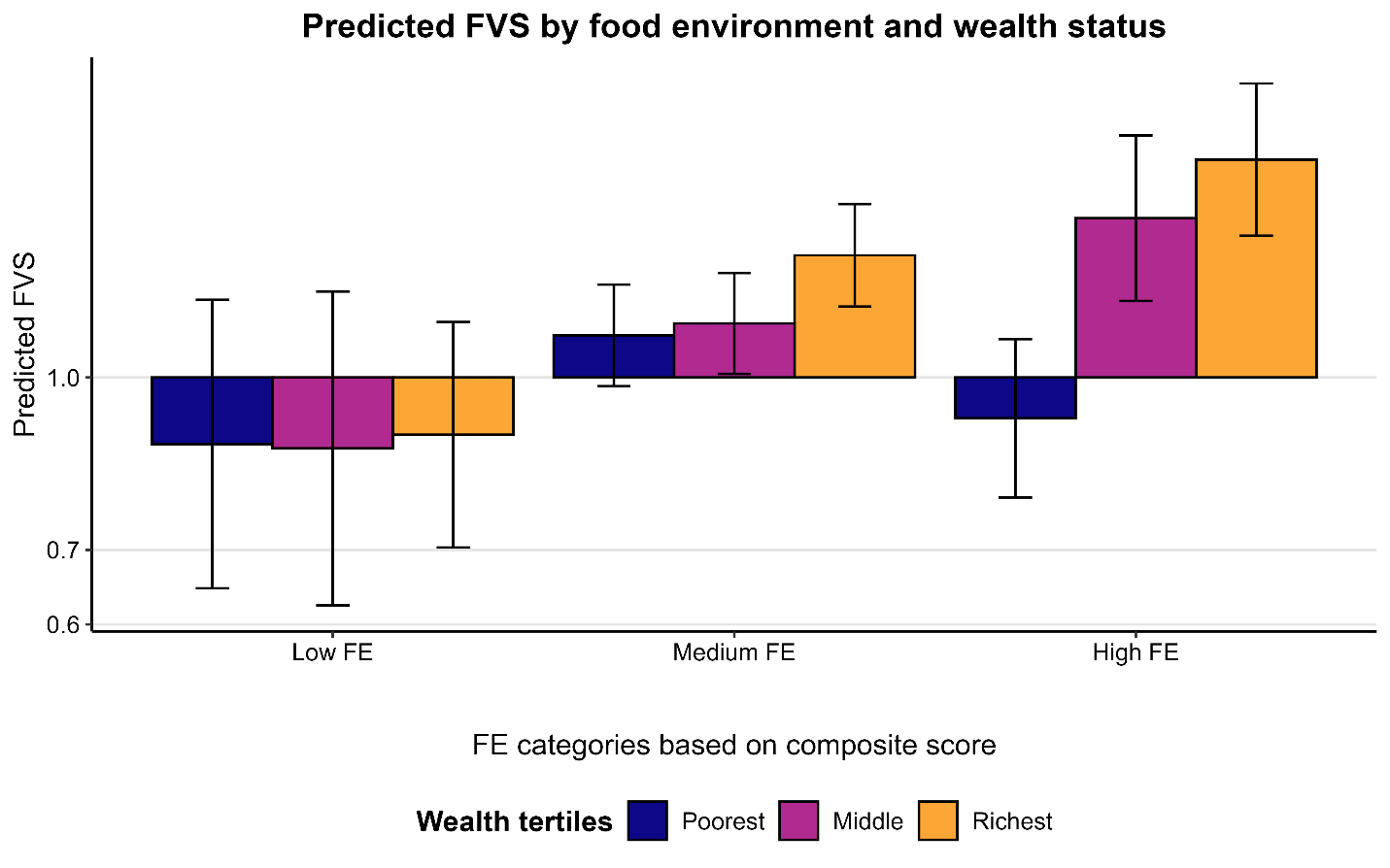
Figure S3


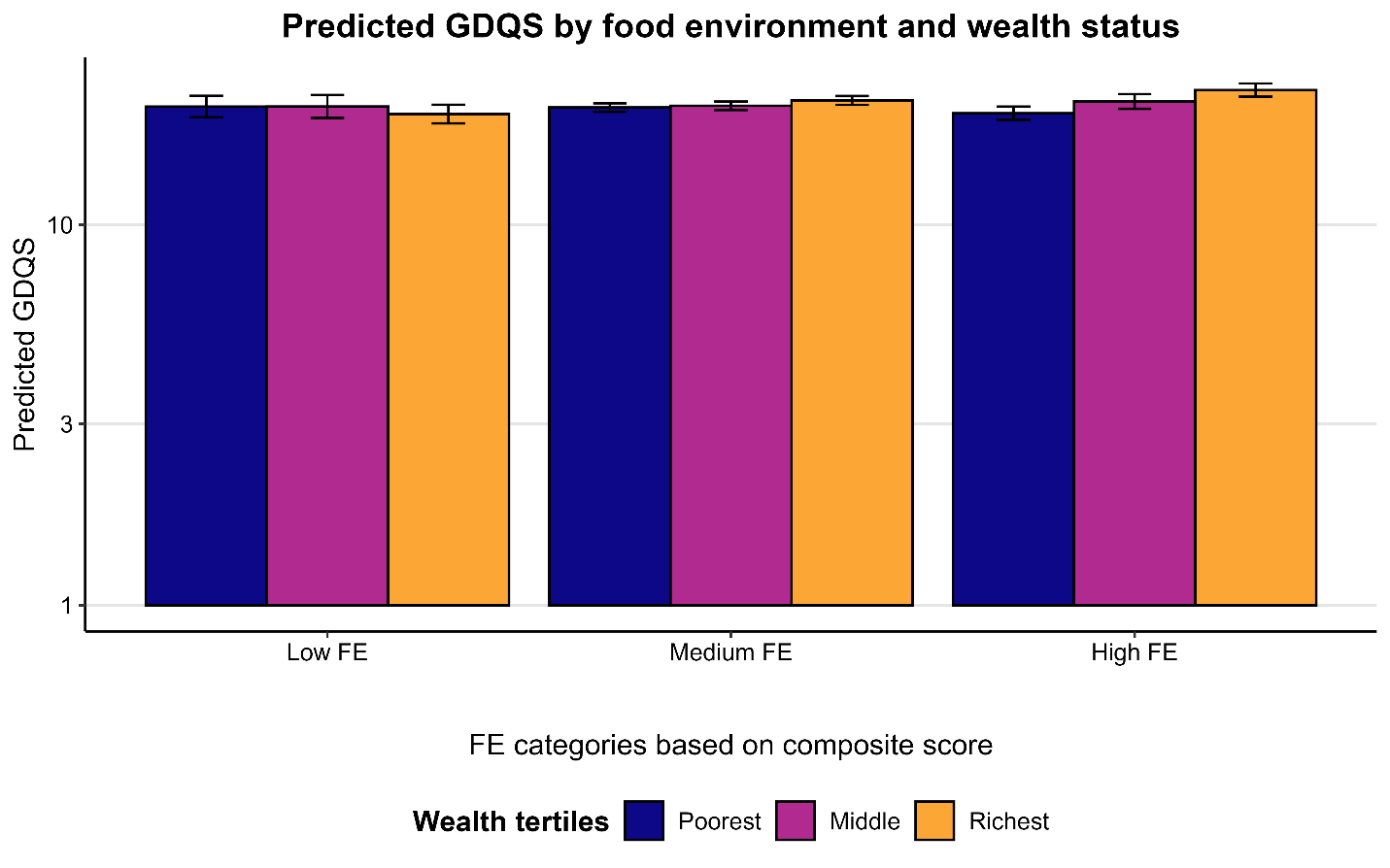
Figure S4


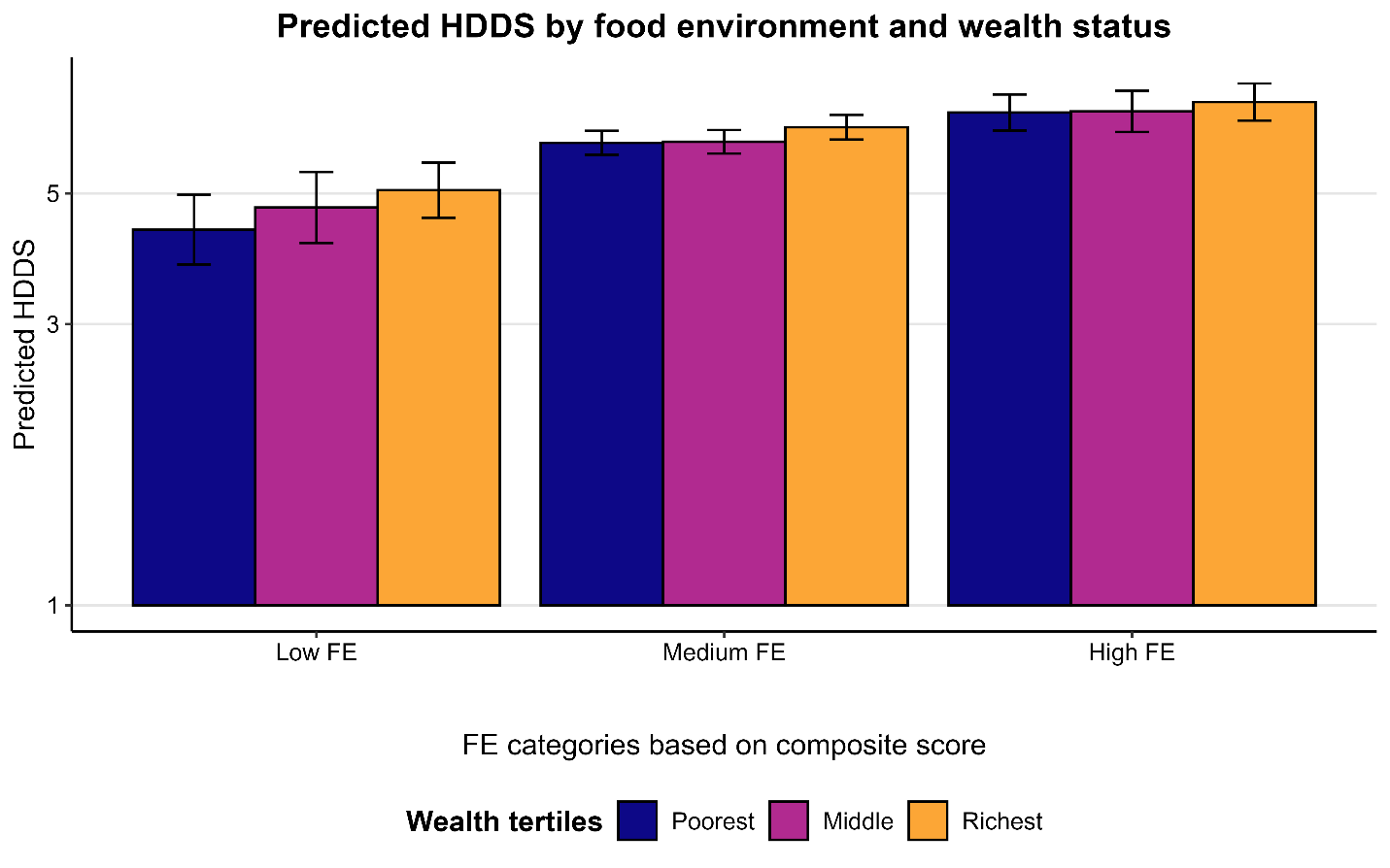
Figure S5
