# Supplementary material for: The influence of the food environment on diet quality: Insights from an extensive household survey in Ethiopia, focusing on women of reproductive age
Source: BMC Nutr. 2025 Jun 2;11:107. doi: 10.1186/s40795-025-01097-z (PMC12128275; doi:10.1186/s40795-025-01097-z)
Supplement: Supplementary file 4 — Additional file 4. Questionnaire utilized to conduct the household survey. [file 40795_2025_1097_MOESM4_ESM.docx]

Additional File 4: Questionnaire utilized to conduct the household survey, Ethiopia

Description

This file contains the household questionnaire used for the dietary analysis in Ethiopia, presented as Appendix S1. The questionnaire, titled "Food System's Approach for Sustainable and Healthy Diet Policy in Ethiopia: Household Questionnaire," includes modules on household identification, socio-demographic characteristics, individual factors, food supply chains, consumer behaviour, social welfare, and access to basic services. It also includes Diet Quality Questionnaires (DQQ) for women and adolescent girls, as well as Food Frequency Questionnaires (FFQ) for caregivers/women and children aged 6-59 months.

Appendix S1: Food System's Approach for Sustainable and Healthy Diet Policy in Ethiopia: Household Questionnaire

# **Module One****: Household Identification and socio-demographic characteristics**

## Geographical location

| **1** | | | | **2** | | | **3** | | | **4** | | **5** | | | **6** | | | | | **7** | | |
| --- | --- | --- | --- | --- | --- | --- | --- | --- | --- | --- | --- | --- | --- | --- | --- | --- | --- | --- | --- | --- | --- | --- |
| **Region** | | | | **Zone** | | | **Woreda** | | | **Town (For rural code 8)** | | **Sub-city (For rural code 88)** | | | **Kebele/FA** | | | | | **EA** | | |
|  | | | |  | | |  | | |  | |  | | |  | | | | |  | | |
|  | **Code** | |  | | **Code** | |  | **Code** | |  | **Code** |  | **Code** | |  | | **Code** | | |  | **Code** | |
|  |  |  |  |  |  |  |  |  |  |  |  |  |  |  |  |  |  |  |  |  |  |  |
| **8** | | | | | **9** | | | **10** | | **11** | | | | | | **12** | | | | | | |
| **Household ID** | | | | | **Household Size** | | |  | | **Household Head Name** | | | | | | **Village name where the HH lives** | | | | | | |
|  | | | | |  | | |  | |  | | | | | |  | | | | | | |
|  |  |  |  | |  |  |  |  |  |  | | | | | |  | | | | | | |
|  | | | | | | | | | | | | | | | | | | | | | | |

| 13 | Household Address GPS Reading | | | | | | | | | |
| --- | --- | --- | --- | --- | --- | --- | --- | --- | --- | --- |
|  |  | | Degree | | Minute | | Second | | | |
|  |  | N |  |  |  |  |  |  |  |  |
|  |  | E |  |  |  |  |  |  |  |  |
| 14 | Accuracy |  | | | | | | Meter | | |
| 15 | Altitude |  | | | | | | Meter | | |
|  | | | | | | | | | | |
| 16 | Supervisor Name |  | | | |  | | | | |
| 17 | Enumerator Name |  | | | |  | | | | |
|  |  |  | | | |  | | | | |

| **Code A** | **Relationship to the HHH** |  | **Code C** | **Type of disability** |  | **Code D** | **Marital Status** |
| --- | --- | --- | --- | --- | --- | --- | --- |
| 0 | Household head |  | 1 | Both eye blind |  | 1 | Never married |
| 1 | Spouse/ Live as a spouse |  | 2 | Single eye blind |  | 2 | Married |
| 2 | Son/Daughter |  | 3 | Total deafness |  | 3 | Divorced |
| 3 | Grandchild |  | 4 | Partial deafness (one ear not hearing) |  | 4 | Separated |
| 4 | Father/Mother |  | 5 | Unable to speak |  | 5 | Widowed |
| 5 | Sister/Brother |  | 6 | Unable to hear/speak |  | 6 | Living together |
| 6 | Niece/Nephew |  | 7 | Blindness and to hear |  |  |  |
| 7 | Uncle/Aunt |  | 8 | Hand or leg lost/crippled |  |  | |
| 8 | Son/Daughter-in-law |  | 9 | Hand or leg paralyzed |  |  |  |
| 9 | Father/Mother-in-law |  | 10 | Leprosy |  | **Code B** | **Religion** |
| 10 | Sister/Sister-in-law |  | 11 | Mental illness/madness |  | 1 | Orthodox |
| 11 | Grandparents |  | 12 | Mental retardation |  | 2 | Catholic |
| 12 | Other |  | 13 | Epilepsy |  | 3 | Protestant |
| 13 | Relatives |  | 14 | Other type of disability |  | 4 | Islam |
| 14 | Servant |  |  |  |  | 5 | Waqi-Feta |
| 15 | Non-relatives |  |  |  |  | 6 | Traditional |
|  |  |  |  |  |  | 7 | No religion/ Atheist |

| **Code E** | **Education – highest grade completed** |  | **Code E** | **Education – highest grade completed** |
| --- | --- | --- | --- | --- |
| 0 | Pre-School: Kindergarten, 0 grade, nursery |  | 21 | TVET Level 5 |
| 1 | 1^st^ Grade Completed |  | 22 | Diploma |
| 2 | 2^nd^ Grade Completed |  | 23 | Bacherlor’s Degree Program |
| 3 | 3^rd^ Grade Completed |  | 24 | Master’s Degree Program (M.A, MPhil, etc.) |
| 4 | 4^th^ Grade Completed |  | 25 | Doctor of Medicine (MD) |
| 5 | 5^th^ Grade Completed |  | 26 | Juris Doctor (J.D) |
| 6 | 6^th^ Grade Completed |  | 27 | Doctor of Philosophy (PhD) |
| 7 | 7^th^ Grade Completed |  | 28 | Other: Informal education (can read and write but has never been in a regular school) |
| 8 | 8^th^ Grade Completed |  | 29 | Other: Adult literacy program |
| 9 | 9^th^ Grade Completed |  | 30 | Other: Satellite |
| 10 | 10^th^ Grade Completed |  | 31 | Other: Non-regular (can read and write by attending a religious institute such as Kes or Kuran but never attended regular school) |
| 11 | 11^th^ Grade Completed |  | 32 | Other: Not educated |
| 12 | 12 Grade Completed |  | 98 | Don’t know |
| 13 | Professional Certification |  |  |  |
| 14 | 1^st^ year college |  |  | |
| 15 | 2^nd^ year college |  |  |  |
| 16 | 3^rd^ year college |  |  |  |
| 17 | TVET Level 1 |  |  |  |
| 18 | TVET Level 2 |  |  |  |
| 19 | TVET Level 3 |  |  |  |
| 20 | TVET Level 4 |  |  |  |
|  | |  |  | |

## Household characteristics and Socio-demographic status

| 1 | 2 | | 3 | | 4 | | 5 | 6 | 7 | | 8 | 9 | 10 | | 11 | |
| --- | --- | --- | --- | --- | --- | --- | --- | --- | --- | --- | --- | --- | --- | --- | --- | --- |
| MEMBER’S ID NO/CODE |  | FOR 5 OR OVER YEARS AGED | |  |  |  |  |  |  |  |  |  |  |  |  |  |
|  | List of HH member | | Relation to the HH | | Sex | | Date of birth (DD/MM/YY) | Age | Disability status | | | Marital Status | HH members’ Educational Status | | | |
|  | Please give me the names of persons who usually live in your HH and have common cooking arrangements & common HH.  ***[TO HAVE A COMPLETE LISTING PROBE AND ASK TO VERIFY PERSONS SUCH AS INFANTS, OLD AGE, NON- RELATIVES, etc.]*** | | What is the relationship of (**NAME**)?  to the head of the household?  **[Code A]** | | What is the sex of (**NAME**)?  1= Male  2= Female | | When was the date of your birth? | How old is (**NAME**)?  **[If age less than 1 year, Record 0 and**  **If 100 or over record 99]** | Does [NAME] has any disability problem?  1= Yes  2= No  **(If No Skip to 9)** | | What type of Disability does [Name] has?  **[See Disability Code C]** | What is  [**NAME**]’s current marital status  ?  **[Code D]** | Can [**NAME**]  read and write?  1= Yes  2= No  **(Skip to module 2 if 10=No)** | | What is the highest school/grade [**NAME**] has completed?  **[Code E]** | |
| 1 |  | |  | |  |  |  |  |  |  |  |  |  |  |  |  |
| 2 |  | |  | |  |  |  |  |  |  |  |  |  |  |  |  |
| 3 |  | |  | |  |  |  |  |  |  |  |  |  |  |  |  |
| 4 |  | |  | |  |  |  |  |  |  |  |  |  |  |  |  |
| 5 |  | |  | |  |  |  |  |  |  |  |  |  |  |  |  |
| 6 |  | |  | |  |  |  |  |  |  |  |  |  |  |  |  |
| 7 |  | |  | |  |  |  |  |  |  |  |  |  |  |  |  |
| 8 |  | |  | |  |  |  |  |  |  |  |  |  |  |  |  |
| 9 |  | |  | |  |  |  |  |  |  |  |  |  |  |  |  |
| 10 |  | |  | |  |  |  |  |  |  |  |  |  |  |  |  |
| 11 |  | |  | |  |  |  |  |  |  |  |  |  |  |  |  |
| 12 |  | |  | |  |  |  |  |  |  |  |  |  |  |  |  |

# **Module Two: Individual Factors**


## Economic

### 2.1.1. Employment Status of Household Members

The following questions apply only for household member **aged 15 and above**

| 1 | 2 | 3 | 4 | 5 | 6 | 7 | 8 | 9 | 10 | 11 | 12 | 13 |
| --- | --- | --- | --- | --- | --- | --- | --- | --- | --- | --- | --- | --- |
| HH members Code number | List of eligible HH members | During the last 7 days was [NAME] engaged in any kind of job or work for at least one hour (including self-employment and family work with or without pay)?  1= yes 🡪 **skip to 5**  2= No | Reason for not working  **[CODE F]** | Sector/industry of employment  **[Code G]** | Type of employer  **[Code H]** | Average monthly earning in Birr from all sources of income including salary, profits, rent, asset sales, interest and gifts? | During the last 24 hours, how many hours did [NAME] spend in home care activities (e.g., food preparation, cleaning, care for children, going to mills and markets.) | During the last 24 hours, how many hours did [NAME] spend in agricultural activities | During the last 24 hours, how many hours did [NAME] spend in fetching water and collecting firewood | During the last 24 hours, how many hours did [NAME] spend on marketing activities?  -----hrs | During the last 7 days, how many times did [NAME] went to nearby market to buy agricultural inputs or sell agriculture products and/or buy foods?  ----frequency | Does the household employ house maid in the last one month?  1= yes  2= no |
|  |  |  |  |  |  |  |  |  |  |  |  |  |
|  |  |  |  |  |  |  |  |  |  |  |  |  |
|  |  |  |  |  |  |  |  |  |  |  |  |  |
|  |  |  |  |  |  |  |  |  |  |  |  |  |
|  |  |  |  |  |  |  |  |  |  |  |  |  |
|  |  |  |  |  |  |  |  |  |  |  |  |  |
|  |  |  |  |  |  |  |  |  |  |  |  |  |
|  |  |  |  |  |  |  |  |  |  |  |  |  |
|  |  |  |  |  |  |  |  |  |  |  |  |  |
|  |  |  |  |  |  |  |  |  |  |  |  |  |
|  |  |  |  |  |  |  |  |  |  |  |  |  |
|  |  |  |  |  |  |  |  |  |  |  |  |  |
|  |  |  |  |  |  |  |  |  |  |  |  |  |
|  |  |  |  |  |  |  |  |  |  |  |  |  |
|  |  |  |  |  |  |  |  |  |  |  |  |  |

12. Would you please point out the most accurate average monthly income category of the household?

| 01= 0‐1000 | 03= 2501‐5000 | 05 = 7,501‐ 10,000 | 07 = 15,001‐ 20,000 | 09 = 30,001‐50,000 | 11= 75,001‐100,000 |
| --- | --- | --- | --- | --- | --- |
| 02= 1001 ‐2500 | 04= 5,001 ‐7,500 | 06 = 10,001– 15,000 | 08 = 20,001‐ 30,000 | 10= 50,001‐75,000 | 12=100,000+ |

| **Code H** | **Types of Employers** |
| --- | --- |
| 01 | Employer (working employer) |
| 02 | Self Employed – Formal sector |
| 03 | Self Employed – Informal sector |
| 04 | Employed – in private enterprise – Formal sector |
| 05 | Employed – in private enterprise – Informal sector |
| 06 | Employed – in public Sector |
| 07 | Employed – in Local NGO |
| 08 | Employed – in International NGO |
| 09 | Employed – in Religious Institution |
| 10 | Employed – in cooperative/unions |
| 11 | Unpaid family work Unpaid (Economic enterprise) |
| 99 | Other specify |

| **Code F** | **Reasons for not working** |
| --- | --- |
| 1 | Unemployed |
| 2 | Student/Training course |
| 3 | Home maker |
| 4 | Retired |
| 5 | Depend on remittance |
| 6 | Old age |
| 7 | Disability |
| 08 | Sick |
| 99 | Other specify |

| **Code G** | Sector/industry |
| --- | --- |
| 1 | Agriculture, Hunting and Forestry |
| 2 | Mining and Quarrying |
| 3 | Manufacturing |
| 4 | Electricity, Gas, Steam and Air Conditioning Supply |
| 5 | Water supply; Sewerage, Waste Management and Remediation Activities |
| 6 | Construction |
| 7 | Wholesale and Retail trade; Repair of Motor Vehicles and Motorcycles |
| 8 | Transportation and Storage |
| 9 | Hotel and Restaurants |
| 10 | Information and Communication |
| 11 | Financial and Insurance Activities |
| 12 | Real Estate Activities |
| 13 | Professional, Scientific and Technical Activities |
| 14 | Administrative and Support Service Activities |
| 15 | Public Administration and Defense; Compulsory Social Security |
| 16 | Education |
| 17 | Human Health and Social Work Activities |
| 18 | Arts, Entertainment and Recreation |
| 19 | Other community, Social and Personal Service Activities |
| 20 | Private Households with Employed Persons |
| 21 | NGOs |
| 99 | Other specify |

## Situational

### 2.2.1. Wealth and Assets

Please tell us the quantity and value of assets currently owned (not borrowed or rented)

*If the item is jointly owned, record the share of the quantity owned by household.*

|  |  | **Unit** | **Quantity owned** | **If shared, your estimated percentage share?** | **If you sell all of your share, how much would you get? (Birr)** |
| --- | --- | --- | --- | --- | --- |
|  | **Electronic appliances and jewelry** |  |  |  |  |
| 1 | Radio | Count |  |  |  |
| 2 | Tape recorder | Count |  |  |  |
| 3 | Television | Count |  |  |  |
| 4 | Refrigerator | Count |  |  |  |
| 5 | Telephone (Fixed landline) | Count |  |  |  |
| 6 | Mobile phone | Count |  |  |  |
|  | **Energy, furniture and transportation** |  |  |  |  |
| 7 | Improved charcoal or wood stove (Lakech or mirt) | Count |  |  |  |
| 8 | Kerosene stove | Count |  |  |  |
| 9 | Biomass or biogas stove (e.g., dung burning) | Count |  |  |  |
| 10 | Bicycle | Count |  |  |  |
| 11 | Motorbike | Count |  |  |  |
| 12 | Clay oven | Count |  |  |  |
| 13 | Metal oven | Count |  |  |  |
| 14 | Pot | Count |  |  |  |
| 15 | Knife | Count |  |  |  |
| 16 | Mortal and pistol | Count |  |  |  |
| 17 | Hand miller | Count |  |  |  |
| 18 | Chopping board | Count |  |  |  |
| 19 | Bucket | Count |  |  |  |
| 20 | Plate | Count |  |  |  |
| 21 | Cup | Count |  |  |  |
| 22 | Spoon | Count |  |  |  |
| 23 | Fork | Count |  |  |  |
| 24 | Ladle | Count |  |  |  |
| 25 | Electric food processor | Count |  |  |  |
| 26 | Electric bakery | Count |  |  |  |
| 27 | Pressure cooker | Count |  |  |  |
|  | **Livestock** |  |  |  |  |
| 28 | Oxen | Count |  |  |  |
| 29 | Cows | Count |  |  |  |
| 30 | Heifer | Count |  |  |  |
| 31 | Bulls | Count |  |  |  |
| 32 | Young bull or young Heifer | Count |  |  |  |
| 33 | Calf | Count |  |  |  |
| 34 | Sheep | Count |  |  |  |
| 35 | Goats | Count |  |  |  |
| 36 | Donkey | Count |  |  |  |
| 37 | Mules | Count |  |  |  |
| 38 | Horses | Count |  |  |  |
| 39 | Camels | Count |  |  |  |
| 40 | Chicken | Count |  |  |  |
| 41 | Beehive | Queens |  |  |  |
|  | **Productive assets** |  |  |  |  |
| 42 | Plough set | Count |  |  |  |
| 43 | Plow (Maresha) | Count |  |  |  |
| 44 | Sickle —Imported (Albin) | Count |  |  |  |
| 45 | Sickle —Local | Count |  |  |  |
| 46 | Pick axe/spade/shovel—(doma) | Count |  |  |  |
| 47 | Axe | Count |  |  |  |
| 48 | Saw | Count |  |  |  |
| 49 | Hoe | Count |  |  |  |
| 50 | Beehive (Traditional) | Count |  |  |  |
| 51 | Beehive (Modern) | Count |  |  |  |
| 52 | Triddle pump | Count |  |  |  |
| 53 | Drip irrigation | Count |  |  |  |
| 54 | Animal-drawn cart (Gari), wheelbarrow, or donkey cart | Count |  |  |  |
| 55 | Barrel carrying device | Count |  |  |  |
| 56 | Flour mill | Count |  |  |  |
| 57 | Knapsack chemical sprayer | Count |  |  |  |

## Aspirations and Preferences

| **1.** | Do you think that you often eat food items that you like/aspire the most? | 1. Yes 🡪 ask 2 and skip to 6  2. No 🡪 Skip to 3 |
| --- | --- | --- |
| **2.** | **P**lease mention the three food items that you aspire and usually have in your dishes? | 1.  2.  3. |
| **3.** | What is the main reason that you are unable to eat food item/s that you like/aspire the most? | 1. Lack of money  2. Health problem  3. The food item/s are not available  4. High price  99. Other (specify) |
| **4.** | If you don’t have any monetary constraints to get food item/s or you don’t have any health problem/s that prevent you from eating food item/s you like the most, and the foods you like/aspire the most are available in your proximity, please mention three food items that you aspire to have in your dishes **daily**? ______________ | 1.  2.  3. |
| **5.** | If you don’t have any monetary constraints to get food item/s or you don’t have any health problem/s that prevent you from eating food item/s you like the most, and the foods you like/aspire the most are available in your proximity, please mention three food items that you aspire to have in your dishes **usually**? ___________ | 1.  2.  3. |

| **6. Risk preferences** |  |
| --- | --- |
| Please tell me, in general, how willing are you to take risks (for example, agricultural risks such as trying new seeds that has a higher yield but are less drought resistant, new food items not popular in your community), using a scale from 0 to 10, where 0 means you are “completely unwilling to take risks” and 10 means you are “very willing to take risks.” You can also use any number between 0 and 10 to indicate where you fall on the scale, using 0, 1, 2, 3, 4, 5, 6, 7, 8, 9, or 10.  Suppose you are sick. You have the choice between two options  Option A: You can get some medicine that will reduce the pain but will not cure you.  Option B: You can get surgery that will cure you; however, there is a small risk of death.  Which option would you choose? |  |
| **7. Time preferences** |  |
| In comparison to others, how willing are you to give up something that is beneficial for you today in order to benefit more from that in the future? Please use a scale from 0 to 10, where a 0 means you are “completely unwilling to give up something today” and a 10 means you are “very willing to give up something today”. You can also use any number between 0 and 10 to indicate where you fall on the scale, using 0, 1, 2, 3, 4, 5, 6, 7, 8, 9, or 10. |  |
| Suppose you are sick due to deficiency of vitamins. The illness is not life-long, but it will last for a couple months. You have the choice between two options.  **Option A:** You can get some manufactured supplementary vitamins today, which will make you feel somewhat better.  **Option B:** You eat foods rich in the required vitamins that will make you feel entirely good but takes relatively long time to heal.  Which option do you choose? |  |

# **Module Three:** **Food supply chains**


## Production systems and input supply

| 1 | Do you have any agricultural land (including any land used for urban agriculture) with holding rights? | 1=Yes  2=No 🡪 skip to Module Four |
| --- | --- | --- |

PARCEL ROSTER

ENUMERATOR: LIST ALL PARCELS

|  |  | 1 | 2 | 3 | | 4 | 5 | 6 | 7 | 8 | 9 | 10 |
| --- | --- | --- | --- | --- | --- | --- | --- | --- | --- | --- | --- | --- |
| ENUMER  ATOR:  "Please  give me  the list of  all  parcels of  land  owned,  rented in  and  rented  out (both  cultivated  and non  cultivated  ) " | PARCEL  ID | PARCEL  DESCRIPTION  List all parcels  of land owned  or rented in  (both  cultivated and  non-cultivated) | What is the  number of  Fields in  this  [Parcel]?  NUMBER | ASK HOLDER: What is the area of  [FIELD]?  **CODES FOR UNIT:**  1=Hectare  square  2=Meters  3=Timad  4=Boy  5=Senga  6=Kert  7=Tilm  8=Medeb  9=Rope (Gemed)  10=Ermija  99=Other (Specify) | | During this season, what  is the status of this  [FIELD]?  1=Cultivated...... 1  2=Pasture .........2  3=Fallow ......... 3  4=Forest ..........4  5=Land Prepared for Belg season  6=Home/homestead  99=Other (specify) | During this season,  What type of crop was planted on this  [FIELD]?  1=Temporary crop  2=Permanent crop  3=Temporary and permanent crop | What was the  method of cropping  in this [FIELD]?  1=Pure stand  2=Mixed crop | ENUMERTAOR: WHAT IS  APPEARANCE OF THIS FIELD?  1=Flat  2=Sloppy-  (moderate)  3=Sloppy-  (steep) | Is [FIELD] under Extension Program  during the current agricultural  season?  1=Yes  2=No | What is the predominant soil type of this [PARCEL]?  READ ANSWERS  1=Leptosoil  2=Cambisoil  3=Vertisoil  4=Luvisoil  5=Mixed Type  6=Other, parcel use (Barn, residential, Etc.)  99=Other Soil Type  (Specify) | Is [FIELD] irrigated during the  current agricultural season?  1=Yes  2=No |
|  |  |  |  | quantity | Unit |  |  |  |  |  |  |  |
|  |  |  |  |  |  |  |  |  |  |  |  |  |
|  |  |  |  |  |  |  |  |  |  |  |  |  |
|  |  |  |  |  |  |  |  |  |  |  |  |  |
|  |  |  |  |  |  |  |  |  |  |  |  |  |
|  |  |  |  |  |  |  |  |  |  |  |  |  |
|  |  |  |  |  |  |  |  |  |  |  |  |  |
|  |  |  |  |  |  |  |  |  |  |  |  |  |
|  |  |  |  |  |  |  |  |  |  |  |  |  |
|  |  |  |  |  |  |  |  |  |  |  |  |  |
|  |  |  |  |  |  |  |  |  |  |  |  |  |
|  |  |  |  |  |  |  |  |  |  |  |  |  |

| 11 | 11a | 11b | 11c | 11d | 12 | 12a | 12b | 12c | 12d |
| --- | --- | --- | --- | --- | --- | --- | --- | --- | --- |
| Do you use  any UREA on  [FIELD] in this  agricultural  season?  1=Yes  2=No  ►Q12 | What is the  quantity of  UREA used  on [FIELD]  in this  agricultural  season? | Did you  purchase  any of the  UREA used  on  [FIELD]?  (Cash or  credit  purchases)  1=Yes  2=No  ►Q12) | How much of  the UREA was  purchased on  cash or  purchased on  credit during  this agricultural  season?  RECORD TOTAL  QUANTITY IN  KG,  REGARDLESS  OF SOURCE. | What was  the value of  all of the  UREA that  you  purchased  on cash or  purchased  on credit  during this  agricultural  season? | Do you use  any DAP on  [FIELD] in  this  agricultural  season?  1=Yes  2=No  ►Q13 | What is the  quantity of  DAP used  on [FIELD]  in this  agricultural  season? | Did you  purchase  any of the  DAP used  on  [FIELD]?  (Cash or  credit  purchases) | How much of  the DAP was  purchased on  cash or  purchased on  credit during  this agricultural  season?  RECORD TOTAL  QUANTITY IN  KG,  REGARDLESS  OF SOURCE. | What was  the value of  all of the  DAP that  you  purchased  on cash or  purchased  on credit  during this  agricultural  season? |
|  | QUANTITY IN KG |  | QUANTITY IN  KG | BIRR |  |  |  |  |  |
|  |  |  |  |  |  | QUANTITY IN KG |  |  |  |
|  |  |  |  |  |  |  |  | QUANTITY IN KG | BIRR |
|  |  |  |  |  |  |  |  |  |  |
|  |  |  |  |  |  |  |  |  |  |
|  |  |  |  |  |  |  |  |  |  |
|  |  |  |  |  |  |  |  |  |  |
|  |  |  |  |  |  |  |  |  |  |
|  |  |  |  |  |  |  |  |  |  |
|  |  |  |  |  |  |  |  |  |  |
|  |  |  |  |  |  |  |  |  |  |
|  |  |  |  |  |  |  |  |  |  |
|  |  |  |  |  |  |  |  |  |  |
|  |  |  |  |  |  |  |  |  |  |

| 13 | 13a | 13b | | 13c | 13d | | 14 | 14a | | 14b | 14c | | 14d |  |
| --- | --- | --- | --- | --- | --- | --- | --- | --- | --- | --- | --- | --- | --- | --- |
| Do you use  any NPS on  [FIELD] in  this  agricultural  season?  1=Yes  2=No  ►Q14 | What is the  quantity of  NPS used  on [FIELD]  in this  agricultural  season? | Did you  purchase  any of the  NPS used  on  [FIELD]?  (Cash or  credit  purchases)  1=Yes  2=No  ►Q14) | | How much of  the NPS was  purchased on  cash or  purchased on  credit during  this agricultural  season?  RECORD TOTAL  QUANTITY IN  KG,  REGARDLESS  OF SOURCE. | What was  the value of  all of the  NPS that  you  purchased  on cash or  purchased  on credit  during this  agricultural  season? | | Do you use  any  blended  chemical  fertilizers  on [FIELD]  in this  agricultural  season?  1=Yes  2=No  ►Q15 | What is the  quantity of  blended  chemical  fertilizers used  on [FIELD]  in this  agricultural  season? | | Did you  purchase  any of the  blended  chemical  fertilizers used  on  [FIELD]?  (Cash or  credit  purchases) | How much of  the blended  chemical  fertilizers was purchased on  cash or  purchased on  credit during  this agricultural  season?  RECORD TOTAL  QUANTITY IN  KG,  REGARDLESS  OF SOURCE. | | What was  the value of  all of the  blended  chemical  fertilizers that  you  purchased  on cash or  purchased  on credit  during this  agricultural  season? |  |
|  | QUANTITY IN KG |  |  | QUANTITY IN  KG | BIRR | |  |  |  |  |  |  |  |  |
|  |  |  |  |  |  |  |  | QUANTITY IN KG | |  |  |  |  |  |
|  |  |  |  |  |  |  |  |  |  |  | QUANTITY IN KG | | BIRR |  |
|  |  |  | |  |  | |  |  | |  |  | |  |  |
|  |  |  | |  |  | |  |  | |  |  | |  |  |
|  |  |  | |  |  | |  |  | |  |  | |  |  |
|  |  |  | |  |  | |  |  | |  |  | |  |  |
|  |  |  | |  |  | |  |  | |  |  | |  |  |
|  |  |  | |  |  | |  |  | |  |  | |  |  |
|  |  |  | |  |  | |  |  | |  |  | |  |  |
|  |  |  | |  |  | |  |  | |  |  | |  |  |
|  |  |  | |  |  | |  |  | |  |  | |  |  |
|  |  |  | |  |  | |  |  | |  |  | |  |  |
|  |  |  | |  |  | |  |  | |  |  | |  |  |
| 15 | | | 16 | | | 17 | | | 18 | | | 19 | | |
| Do you use any manure on [FIELD] in this agricultural season?  1=Yes  2=No | | | Do you use any compost on [FIELD] in this agricultural season?  1=Yes  2=No | | | Do you use any other organic fertilizer on [FIELD]?  1=Yes  2=No | | | For the current season, has any household member worked on this [FIELD] for activities such as land preparation,  planting, ridging,  weeding and  fertilizing?  1=Yes  2=No | | | For the current season, how many days did your household hire labor for activities such as land preparation, planting, ridging, weeding and fertilizing, on this [FIELD]?  1=Yes  2=No | | |
|  | | |  | | |  | | |  | | |  | | |
|  | | |  | | |  | | |  | | |  | | |
|  | | |  | | |  | | |  | | |  | | |
|  | | |  | | |  | | |  | | |  | | |
|  | | |  | | |  | | |  | | |  | | |
|  | | |  | | |  | | |  | | |  | | |
|  | | |  | | |  | | |  | | |  | | |
|  | | |  | | |  | | |  | | |  | | |
|  | | |  | | |  | | |  | | |  | | |
|  | | |  | | |  | | |  | | |  | | |
|  | | |  | | |  | | |  | | |  | | |

| 20 | 21 | 22 | 23 | 24 |
| --- | --- | --- | --- | --- |
| For the current season, has any member of your household worked on any soil conservation measures on the land?  1=Yes  2=No 🡪 Skip to 22 | What type of conservation measures have member of your household used?  1=Indigenous stone bunds (terracing) 2=Indigenous soil bunds  3=Introduced stone bunds (terracing) 4=Introduced soil bunds  5=Contour ploughing  6=Strip cropping  7=Alley cropping (Sesbania)  8=Soil bunds (Planted with Sesbania) 9=Indigenous soil and stone bund 10=Introduced soil and stone bund  99=Other specify | What is the degree of susceptibility of this PARCEL to soil erosion?  1=None  2=Low  3=Moderate  4=High | For the current season, has any member of your household made any long-term investments (e.g., terracing, tree planting, and gully rehabilitation) on this [FIELD]?  1=Yes  2=No 🡪Crop harvest section | What type of long-term investments measures have member of your household used?  1=Irrigation well  2=Irrigation canal  3=Private Pond  4=Leveled  5=Sleared of stones  6=Stone terrace  7=Soil bund  8=Check dam  9=Drainage ditch  10=Ranches  11=Trees planted  12=Grass strips  13=Live fence or barrier  14=Constructed fence  15=Water harvesting  99=Other specify |
|  |  |  |  |  |
|  |  |  |  |  |
|  |  |  |  |  |
|  |  |  |  |  |
|  |  |  |  |  |

|  |  | **1** | | | **2** | **3** | **4** | | **5** | | **6** | | **7** | **8** | |
| --- | --- | --- | --- | --- | --- | --- | --- | --- | --- | --- | --- | --- | --- | --- | --- |
| PARCEL  ID | FIELD  ID | What crop(s) is planted  On [FIELD] in this current  agricultural season?  *COPY ALL CROPS GROWN*  *ON ALL FIELDS FROM THE*  *POST-PLANTING*  *QUESTIONNAIRE, FLAP CCROP*  *ROSTER.* | | | What type of  crop stand was  on the [FIELD]?  READ  RESPONSES  1=Pure stand  (► Q4)  2=Mixed stand | Approximately, how  much of the [FIELD]  was planted with  [CROP]?  1= Less than 1/4  2= 1/4  3= 1/2  4= 3/4  5= More than 3/4 | Have you  harvested any of  the [CROP] from  this [FIELD]?  (For permanent  crop harvested  between XXXXX, XXXX to  XXXX, XXXX) | | How much [CROP]  have you harvested  from this [FIELD]?  THE ESTIMATE OF  HARVEST SHOULD BE  FOR FINAL FORM OF  THE CROP.  SEE UNIT  CODES ABOVE | | WHAT IS THE  ESTIMATED  HARVESTED  CROP IN KG  (IF THE  VALUE  GIVEN IN Q5  IS IN NON  STANDARD  UNIT) | | What is the current  condition of this harvest  [CROP]?  1= Picked (e.g.  Coffee/Chat).1  2= Cut (but not yet  Piled) ..............2  3= Piled/Unshelled/Not  yet threshed  4= Shelled/threshed  99= Other (specify) | What were the  months when the  harvest started and  ended?  1=September  2=October  3=November  4=December  5=January  6=February  7=March  8=April  9=May  10=June  11=July  12=August  13=Pagume | |
|  |  | CROP NAME | | CODE |  | **%** |  |  | QUANTITY | UNIT  CODE | **KG** | |  | **START** | **END** |
|  | | | | | | | | | | | | | | | |
|  |  |  |  | |  |  |  |  |  | |  |  |  |  |  |
|  |  |  |  | |  |  |  |  |  | |  |  |  |  |  |
|  |  |  |  | |  |  |  |  |  | |  |  |  |  |  |
|  |  |  |  | |  |  |  |  |  | |  |  |  |  |  |
|  |  |  |  | |  |  |  |  |  | |  |  |  |  |  |
|  |  |  |  | |  |  |  |  |  | |  |  |  |  |  |
|  |  |  |  | |  |  |  |  |  | |  |  |  |  |  |
|  |  |  |  | |  |  |  |  |  | |  |  |  |  |  |
|  |  |  |  | |  |  |  |  |  | |  |  |  |  |  |
|  |  |  |  | |  |  |  |  |  | |  |  |  |  |  |
|  |  |  |  | |  |  |  |  |  | |  |  |  |  |  |
|  |  |  |  | |  |  |  |  |  | |  |  |  |  |  |

## Storage and distribution

| 1 | 2 | 3 | | 4 | | 5 | 6 | 7 |
| --- | --- | --- | --- | --- | --- | --- | --- | --- |
| Did you sell  any of the  harvested  [CROP]?  1=Yes  2=No 🡪 question 8 | Which crops did you sell? | Who/What were the main buyers/outlets for your [CROP] sales?  LIST UP TO 2 OPTIONS  1= Farmer-Based Club/Organization  2=Agricultural Cooperative  3=Government Agency  4=Local Market  5=Main Market  6=Relative  7=Friend/Neighbor  8=NGO  9=Roadside  99=Other (Specify) | | How much of the  harvested [CROP] was  sold in total? | | What was the  total value of all  [CROP] sales?  ESTIMATE THE  VALUE OF INKIND  PAYMENTS. | What was the main  mode of  transportation  associated with all [CROP] sales?  READ RESPONSES  1=Foot  2=Transport  3=Pack Animals  4=Own Buyer Pick-Up  99=Other (Specify) | What was the  total cost of  transportation  associated with  all [CROP] sales?  INLUDE ALL  TRIPS FROM  AND BACK TO  THE FARM.  IF NOTHING,  RECORD ZERO |
|  |  | #1 | #2 | QTY. | UNIT CODE | BIRR |  | BIRR |
|  |  |  |  |  |  |  |  |  |
|  | | | | | | | | |
|  |  |  | |  | |  |  |  |
|  |  |  | |  | |  |  |  |
|  |  |  | |  | |  |  |  |
|  |  |  | |  | |  |  |  |
|  |  |  | |  | |  |  |  |
|  |  |  | |  | |  |  |  |
|  |  |  | |  | |  |  |  |
|  |  |  | |  | |  |  |  |

| 8 | 9 | 10 | | 11 | | 12 | | | | | 13 | | 14 | |
| --- | --- | --- | --- | --- | --- | --- | --- | --- | --- | --- | --- | --- | --- | --- |
| Do you have  any of the  harvested  [CROP] in  storage now?  1=Yes  2=No🡪  NEXT CROP | What is your main method of storage for this crop?  READ RESPONSES  1=Unprotected pike  2=Heaped in house  3=Bags in house  4=Metallic silo  5=Other silos (modern or traditional)  6=Pit  7=Rented storage  99=Other (Specify) | How much of the  harvested [CROP]  during the  [CURRENT  AGRICULTURE  SEASON] is being  stored by your  household?  Do you use any compost on [FIELD] in this agricultural season?  SEE UNIT  CODES ABOVE | | What did you do to protect the stored [CROP]?  READ RESPONSES. LIST  UP TO 2.  1=Spraying  2=Smoking  3=Hired guard  4=Did nothing  5=Elevation  99=Other  (specify) | | OF the total quantity of harvested crop currently in your storage (of the total quantity reported in Q4) What proportion will you use for……. | | | | | How much of the harvested [CROP] during the [CURRENT AGRICULTURE SEASON] was lost due to rotting, insects, rodents, theft, etc. in the post-harvest period? (If no  damage record "0" and go to  Q23)  INCLUDE THE QUANTITY OF CROP  USED AS ANIMAL FEED DUE TO  PEST DAMAGE.  SEE UNIT  CODES ABOVE | | What was the reason  for loss?  READ RESPONSES. LIST  UP TO 2.  1=Rotting  2=Insects  3=Rodents/Pests  4=Flood  5=Theft  99=Other  (Specify) | |
|  |  |  |  |  |  | Sales | Household  consumption | reimbursements for Land, labour, or reimbursements for inputs | Other Use  (SEED, GIFT,  ANIMAL FEED ETC) | Total | QTY. | UNIT CODE | 1ST | 2ND |
|  |  | QTY. | UNIT CODE | 1ST | 2ND | % | % | % | % | % |  | |  | |
|  | | | | | |  | | | | |  | |  | |
|  |  |  |  |  | |  | | | | |  | |  | |
|  |  |  |  |  | |  | | | | |  | |  | |
|  |  |  |  |  | |  | | | | |  | |  | |
|  |  |  |  |  | |  | | | | |  | |  | |
|  |  |  |  |  | |  | | | | |  | |  | |
|  |  |  |  |  | |  | | | | |  | |  | |
|  |  |  |  |  | |  | | | | |  | |  | |
|  |  |  |  |  | |  | | | | |  | |  | |
|  |  |  |  |  | |  | | | | |  | |  | |

## Livestock By-product Utilization During the Last 12 Months

|  | 1 | 2 | | | | |
| --- | --- | --- | --- | --- | --- | --- |
|  | In the last 12 months,  did you produce any  [BY‐PRODUCT] from  your livestock?  1=Yes  2=No 🡪 next by  product  IF NONE (►NEXT SECTION) | What proportion of livestock and livestock products have you used for … | | | | |
|  |  | Household  consumption | Sale | Wage  In-kind | Other | Total |
|  |  | % | % | % | % | % |
| **LIVESTOCK BY PRODUCT** | CODE |  |  |  |  |  |
|  |  |  |  |  |  |  |
| Butter | 1 |  |  |  |  |  |
| Cheese | 2 |  |  |  |  |  |
| Meat | 3 |  |  |  |  |  |
| Beef | 4 |  |  |  |  |  |
| Mutton/Goat | 5 |  |  |  |  |  |
| Camel Meat | 6 |  |  |  |  |  |
| Eggs | 7 |  |  |  |  |  |
| Honey | 8 |  |  |  |  |  |
| Wax | 9 |  |  |  |  |  |
| Wool (Sheep Hair) | 10 |  |  |  |  |  |
| Skin | 11 |  |  |  |  |  |
| Hides | 12 |  |  |  |  |  |
| Arera | 13 |  |  |  |  |  |
| Aguat | 14 |  |  |  |  |  |
| Others | 15 |  |  |  |  |  |

## Processing and packaging

Which of the following options describes best your situation?

| 1 | Handling | 1= Poor handling of produce  2= Absence of produce sorting (not sorting damaged and good produce)  3=Use of outdated or traditional technology in postharvest handling  4= Poor timing of harvesting  5=None  98=Do not know  99=Other specify |
| --- | --- | --- |
| 2 | Packaging | 1= Inadequate packaging in storage and transport (e.g., use of packaging not suitable for long term storage or long-distance transportation)  2=Low technology packaging (e.g., use of traditional packaging material that may cause damage to product during handling, storage and transportation)  3= Inappropriate use of packaging (e.g., overfilling of product packages and wrong stacking of packages causing bruises, dents, punctures in produce; mixing of products)  4=None  98=Do not know  99= Other specify |

## Retail and marketing

| 1 | Where do you usually sell your produce (crops, livestock and livestock products)? | 1=Local market in my village  2=District crop or livestock market  3=Regional crop or livestock market  4= Directly export to international market  5=None  99= Other specify |
| --- | --- | --- |
| Which of the following options describes best your situation? | | |
| 2 | Market information/product pricing | 1= Lack of information on prevailing product price  2= Peak season – low pricing (overflowing of local markets of product abundance during peak season causes surpluses in the market and prices to plummet, creating losses)  3= No price premium on quality (local market does not pay rewards on quality)  4= High dependence on middlemen about the price of the produce  5= Absence of (well-functioning) marketing system that leads to a wide variation in prices  6=None  98=Do not know  99=Other specify |
| 3 | Infrastructure and connectivity | 1=Absence of quality roads, particularly in rainy seasons  2=Underdevelopment and unreliability of energy sources (electricity)  3= Underdevelopment of banking and finance system  4=Lack of irrigation schemes and water supplies  5=Lack of product and input markets  6=None  98=Do not know  99= Other specify |
| 4 | Chain length | 1=Large number of middlemen leading to long supply chains  2=Dominant role of middlemen - creating dependence of producers, low pricing and cutting of producers from the supply chain  3=Small price margins due to long chain  4=None  98=Do not know  99=Other specify |
| 5 | Crop protection | 1=Absence of pre-harvest disease management  2=Absence of post-harvest disease management 3=Moulds, bacteria, pests and weather 4=None  98=Do not know  99=Other specify |

#

# **Module Four:** **Food Environment**


## Food availability and affordability

| Food ID | COMODITY TYPE | Is enough [item] available at a reasonable distance from your location (60 minutes)?  1=Yes  2=No | Food consumption in the last 7 days | | | | | How much of [item] is consumed by the household in the last 24 hours?  UNIT CODE |
| --- | --- | --- | --- | --- | --- | --- | --- | --- |
|  |  |  | QTY from: | | | Unit | Total expenditure (value) |  |
|  |  |  | 1. Own production | 1. Gift | 1. Purchase |  |  |  |
|  | 1. **1 CEREALS** |  |  |  |  |  |  |  |
| 101 | Teff |  |  |  |  |  |  |  |
| 102 | Barley (Incl. Beso: roasted & milled barely) |  |  |  |  |  |  |  |
| 103 | Wheat (Incl. Flour factory product) |  |  |  |  |  |  |  |
| 104 | Maize |  |  |  |  |  |  |  |
| 105 | Sorghum |  |  |  |  |  |  |  |
| 106 | Millet |  |  |  |  |  |  |  |
| 107 | Rice |  |  |  |  |  |  |  |
| 108 | Oats |  |  |  |  |  |  |  |
| 109 | Other cereal (Specify) |  |  |  |  |  |  |  |
|  | **2 PULSES & NUTS** |  |  |  |  |  |  |  |
| 201 | Horsebeans |  |  |  |  |  |  |  |
| 202 | Chickpea |  |  |  |  |  |  |  |
| 203 | Field Pea |  |  |  |  |  |  |  |
| 204 | Lentils |  |  |  |  |  |  |  |
| 205 | Haricot Beans |  |  |  |  |  |  |  |
| 206 | Ground nuts |  |  |  |  |  |  |  |
| 207 | Vetch |  |  |  |  |  |  |  |
| 208 | Fenugreek |  |  |  |  |  |  |  |
| 209 | mung bean |  |  |  |  |  |  |  |
| 210 | Processed pulses (Shiro) |  |  |  |  |  |  |  |
| 211 | Other pulse or nut (Specify) |  |  |  |  |  |  |  |

| Food ID | COMODITY TYPE | Is sufficient quantity of [item] available at a reasonable distance from your location?  1=Yes 2=No | | Food consumption in the last 7 days | | | | | How much of [item] is consumed by the household in the last 24 hours?  UNIT CODE | |
| --- | --- | --- | --- | --- | --- | --- | --- | --- | --- | --- |
|  |  |  |  | QTY from: | | | Unit | Total expenditure (value) |  |  |
|  |  |  |  | Own production | Gift | 1. Purchase |  |  |  |  |
|  | 1. **3 OIL SEEDS (UNPROCESSED)** |  | |  |  |  |  |  |  | |
| 301 | Niger Seed |  | |  |  |  |  |  |  | |
| 302 | Linseed |  | |  |  |  |  |  |  | |
| 303 | SESAME |  | |  |  |  |  |  |  | |
| 304 | Sunflower |  | |  |  |  |  |  |  | |
| 305 | Other seed (Specify) |  | |  |  |  |  |  |  | |
|  | **4 VEGETABLES** |  | |  |  |  |  |  |  | |
| 401 | Onion |  | |  |  |  |  |  |  | |
| 402 | Green chili pepper (kariya) |  | |  |  |  |  |  |  | |
| 403 | Red pepper (Processed pepper (Berbere)) |  | |  |  |  |  |  |  | |
| 404 | kale, cabbage, Pumpkin Leaf, Lettuce, spinach |  | |  |  |  |  |  |  | |
| 405 | Tomato |  | |  |  |  |  |  |  | |
| 406 | Garlic |  | |  |  |  |  |  |  | |
| 407 | Moringa/Shiferaw/Halloka |  | |  |  |  |  |  |  | |
| 408 | Other vegetable (Specify) |  | |  |  |  |  |  |  | |
| **5** | **FRUITS** |  | |  |  |  |  |  |  | |
| 501 | Banana |  | |  |  |  |  |  |  | |
| 502 | Orange |  | |  |  |  |  |  |  | |
| 503 | Mango |  | |  |  |  |  |  |  | |
| 504 | Papaya |  | |  |  |  |  |  |  | |
| 505 | Avocado |  | |  |  |  |  |  |  | |
| 506 | Other fruit (Specify) |  | |  |  |  |  |  |  | |
| **6** | **TUBERS & STEMS** |  | |  |  |  |  |  |  | |
| 601 | Potato |  | |  |  |  |  |  |  | |
| 602 | Kocho |  | |  |  |  |  |  |  | |
| 603 | Bula |  | |  |  |  |  |  |  | |
| 604 | Sweet potato |  | |  |  |  |  |  |  | |
| 605 | Boye/Yam |  | |  |  |  |  |  |  | |
| 606 | Cassava |  | |  |  |  |  |  |  | |
| 607 | Godere |  | |  |  |  |  |  |  | |
| 608 | Carrot |  | |  |  |  |  |  |  | |
| 609 | Beetroot |  | |  |  |  |  |  |  | |
| 610 | Another tuber or stem (Specify) |  | |  |  |  |  |  |  | |
| **7** | **MEAT, POULTRY, Diary, FISH & Other Condiments** |  |  | |  |  |  |  |  |  |
| 701 | Goat & mutton meat |  |  | |  |  |  |  |  |  |
| 702 | Beef |  |  | |  |  |  |  |  |  |
| 703 | Poultry |  |  | |  |  |  |  |  |  |
| 704 | Fish |  |  | |  |  |  |  |  |  |
| 705 | Milk |  |  | |  |  |  |  |  |  |
| 706 | Cheese |  |  | |  |  |  |  |  |  |
| 707 | Butter/ghee |  |  | |  |  |  |  |  |  |
| 708 | Oils (processed) |  |  | |  |  |  |  |  |  |
| 709 | Eggs |  |  | |  |  |  |  |  |  |
| 710 | Sugar |  |  | |  |  |  |  |  |  |
| 11 | Honey, natural |  |  | |  |  |  |  |  |  |
| 712 | Salt |  |  | |  |  |  |  |  |  |
| 713 | Other condiments |  |  | |  |  |  |  |  |  |
| **8** | **BEVERAGES & STIMULANTS** |  |  | |  |  |  |  |  |  |
| 801 | Coffee |  |  | |  |  |  |  |  |  |
| 802 | Tea |  |  | |  |  |  |  |  |  |
| 803 | Soft drinks/Soda |  |  | |  |  |  |  |  |  |
| 804 | Beer |  |  | |  |  |  |  |  |  |
| 805 | Tella |  |  | |  |  |  |  |  |  |
| 806 | Chat/Kat |  |  | |  |  |  |  |  |  |
| 807 | Hops (gesho) |  |  | |  |  |  |  |  |  |
| **9** | **Other Prepared food** |  |  | |  |  |  |  |  |  |
| 901 | Purchased Injera |  |  | |  |  |  |  |  |  |
| 902 | Purchased bread/biscuit |  |  | |  |  |  |  |  |  |
| 903 | Pasta/Maccaroni |  |  | |  |  |  |  |  |  |
| 904 | Other purchased prepared food |  |  | |  |  |  |  |  |  |

| ***CONSUMPTION UNITS*** | | | | | | |
| --- | --- | --- | --- | --- | --- | --- |
| **UNIT SIZE** | | **UNIT CODE** |  | **UNIT** | **SIZE** | **UNIT CODE** |
| Kilogram | | 1 |  | Kunna/Mishe/Kefer/Enkib | Small | 111 |
| Gram | | 2 |  | Kunna/Mishe/Kefer/Enkib | Medium | 112 |
| Litres | | 4 |  | Kunna/Mishe/Kefer/Enkib | Large | 113 |
| Centilitres | | 5 |  |  | | |
|  | | |  | Medeb | Small | 131 |
| Jog | | 8 |  | Medeb | Medium | 132 |
| Melekiya | | 9 |  | Medeb | Large | 133 |
|  | | |  |  | | |
| Birchiko | Small | 31 |  | Piece/number | Small | 141 |
| Birchiko | Medium | 32 |  | Piece/number | Medium | 142 |
| Birchiko | Large | 33 |  | Piece/number | Large | 143 |
|  | | |  |  | | |
| Esir | Small | 61 |  | Sahin | Small | 151 |
| Esir | Medium | 62 |  | Sahin | Medium | 152 |
| Esir | Large | 63 |  | Sahin | Large | 153 |
|  | | |  |  | | |
| Festal | Small | 71 |  | Sini | Small | 171 |
| Festal | Medium | 72 |  | Sini | Large | 172 |
| Festal | Large | 73 |  |  | | |
|  | | |  | Tasa/Tanika/Shember/Selemon | Small | 181 |
| Kerchat/Kemba | Small | 91 |  | Tasa/Tanika/Shember/Selemon | Medium | 182 |
| Kerchat/Kemba | Medium | 92 |  | Tasa/Tanika/Shember/Selemon | Large | 183 |
| Kerchat/Kemba | Large | 93 |  |  | | |
|  | | |  | Zorba/Akara | Small | 191 |
| Kubaya/Cup | Small | 101 |  | Zorba/Akara | Medium | 192 |
| Kubaya/Cup | Medium | 102 |  | Zorba/Akara | Large | 193 |
| Kubaya/Cup | Large | 103 |  |  | |  |
|  | | | | Other (Specify) | | 900 |

## Food messaging

Have you ever heard the following food messages from extension workers, in radio or Television?

| FBDG Key Messages | | |
| --- | --- | --- |
| 1 | Diversify your diet by selecting from at least 4 food groups in every meal and 6 food groups every day.  በእያንዳንዱ የምግብ ገበታዎ ላይ ቢያንስ አራቱን የምግብ መደቦች እንዲሁም በቀን ከሚመገቡት ምግቦች የምግብ ዝርዝር ውስጥ ከስድስቱን የምግብ መደቦች ይመገቡ፡፡ | 1=Yes 2=No 98=Do not know |
| 2 | Every day eat 80–120 grams of legumes such as beans, chickpeas, peas or lentils. This means; 1 medium scoop of 1 medium ladle shiro/lentils/Pea split/ stew or 3 average adult handful of roasted beans/Chickpeas/peas daily  በየቀኑ ከ 80-120 ግራም የጥራጥሬ ዘሮችን ማለትም ባቄላ፣ ሽምብራ፣ አተር ፣ምስርን ይመገቡ፡፡ *ይህ ማለት፡- 1 መካከለኛ ጭልፋ ሽሮ/ምስር/አተር ክክ ወጥ ወይም 3 እፍኝ የባቄላ/ሽምብራ/አተር ቆሎ በቀን ውስጥ ይመገቡ።* | 1=Yes 2=No 98=Do not know |
| 3 | Eat 100–200 grams of various fruits and vegetables of different colours every day, such as bananas, papayas, kale, carrots and tomatoes. This means; 1 medium-sized banana or 1 medium-sized spoon of salad/ 1 medium-sized scoop of cooked  Kaleሞረ 1 glass of homemade mixed fruit juice daily  በየቀኑ ከ 100-200 ግራም የተለያዩ ቀለማት ያሏቸውን ፍራፍሬዎች እንደ ሙዝ፣ፓፓያ፣የአበሻ ጎመን፣ካሮት እና ቲማቲም ይመገቡ፡፡ *ይህ ማለት፡- 1 መካከለኛ ሙዝ ወይም 1 መካከለኛ ጭልፋ የበሰለ አትክልት/ጎመን/ሳላድ/ ወይም 1 ብርጭቆ የፍርፍሬ ጭማቂ በቀን ውስጥ ይመገቡ።* | 1=Yes 2=No 98=Do not know |
| 4 | Diversify your diet with 10–20 grams of nuts and oil seeds such as groundnuts, and sunflower or sesame seeds. This means; 1 tablespoon of Sunflower juice mixed with pieces of injera or 1 average person’s handful of groundnuts daily  ከ 10 -20 ግራም ለውዝና የቅባት እህሎች ማለትም ሱፍ፣ለውዝና ሰሊጥን ይመገቡ፡፡ *ይህ ማለት፡-1 የሾርባ ማንኪያ የሱፍ/የተልባ ፍትፍት ወይም 1 እፍኝ ለውዝ በቀን ውስጥ ይመገቡ።* | 1=Yes 2=No 98=Do not know |
| 5 | Add animal-source foods such as eggs and meat (60 grams) and dairy foods (300-400 grams) to your meals every day. This means; 1 medium-sized ladle of meat stew or 2 glasses of milk or 1 cup of yogurt daily  የእንስሳት ተዋጽዖ ምርቶችን ለምሳሌ እንቁላልና ስጋ 60 ግራም እንዲሁም የወተት ተዋጽዖዎችን ከ300-400 ግራም በየቀኑ ምግብዎ ላይ ይጨምሩ፡፡ *ይህ ማለት፡- 1 መካከለኛ ጭልፋ የስጋ ወጥ ወይም 2 ስኒ ወተት ወይም 1 ብርጭቆ እርጎ በቀን ውስጥ ይመገቡ።* | 1=Yes 2=No 98=Do not know |
| 6 | Drink 8-10 large glasses of clean water daily. It is good for your health.  በየቀኑ ከ 8-10 ብርጭቆ ንጹህ ውሃ ይጠጡ፡፡ | 1=Yes 2=No 98=Do not know |
| 7 | Be physically active for at least 30 minutes a day.  በየቀኑ ቢያንስ ለ30 ደቂቃ ያህል የአካል እንቅስቃሴ ያድርጉ ፤ ንቁ ይሁኑ፡፡ | 1=Yes 2=No 98=Do not know |
| 8 | Take up to 15–20 grams of fats and oils per day. This means; 1 Tablespoon of oil or 1 Tablespoon of spiced butter daily  በየቀኑ ከ 15–20ግራም ፈሳሽ ዘይትና እና ቂቤ ይመገቡ፡፡ *ይህ ማለት፡- 2 የሾርባ ማንኪያ ዘይት ወይም 1 የሾርባ ማንኪያ ቂቤ ይመገቡ ።* | 1=Yes 2=No 98=Do not know |
| 9 | Limit intake of sugar, sweets and soft drinks to below 30 grams per day.  የስኳር፣የጣፋጭ ምግቦችና የለስላሳ መጠጦች አወሳሰድ መጠንዎ ከ 30 ግራም በታች እንዲሆን ያድርጉ፡፡ | 1=Yes 2=No 98=Do not know |
| 10 | Limit salt intake to below 5 grams per day. This means; limit the daily salt intake below a teaspoon.  በየቀኑ ከ 5 ግራም በታች ጨው ይጠቀሙ፡፡ *ይህ ማለት፡- የቀን የጨው አጠቃቀመወዎን ከ 1 የሻይ ማንኪያ በታች ያድርጉ ማለት ነው።* | 1=Yes 2=No 98=Do not know |
| 11 | Limit alcoholic drinks – both factory-processed and homemade – to no more than 2 glasses per week.  በፋብሪካም ይሁን የቤት ውስጥ የሚዘጋጁ የአልኮል መጠጦች አወሳሰድዎን ይመጥኑ ፤ በሳምንት ከሁለት ብርጭቆ እንዳይበልጥ ይጠንቀቁ፡፡ | 1=Yes 2=No 98=Do not know |

# **Module Five****: Diets and nutrition**


## Household Food insecurity

## Months of Adequate Household Food Provisioning

Now I would like to ask you about your household’s food supply during different months of the year. When responding to these questions, please think back over the last 12 months, starting with the current month until the same time last year.

| **No.** | **QUESTION** | **CODING CATEGORIES** | **SKIP** |
| --- | --- | --- | --- |
|  | Were there month(s), in the past 12 months, in which you did not have enough food to meet your family’s needs? | 0=No  1=Yes | 0**➞** M14 |
| Which were the months in the past 12 months during which you did not have enough food to meet your family’s needs? | | | |

To the RA: This includes any kind of food from any source, such as own production, purchase or exchange, food aid, or borrowing. Do not read the list of months aloud. Use a seasonal calendar if needed to help respondent remember the different months. Probe to make sure the respondent has thought about the entire past 12 months.

Identify the current month (mm/yyyy) *):* /

Identify the month that marks the beginning of this 12-month period(mm/yyyy)

| **No** | **Month** | **Response** |
| --- | --- | --- |
|  | January | 1=Yes 2=No |
|  | February | 1=Yes 2=No |
|  | March | 1=Yes 2=No |
|  | April | 1=Yes 2=No |
|  | May | 1=Yes 2=No |
|  | June | 1=Yes 2=No |
|  | July | 1=Yes 2=No |
|  | August | 1=Yes 2=No |
|  | September | 1=Yes 2=No |
|  | October | 1=Yes 2=No |
|  | November | 1=Yes 2=No |
|  | December | 1=Yes 2=No |

## Household Food insecurity

| Now I would like to ask you some questions about food. During the last 12 MONTHS, was there a time when: | | | |
| --- | --- | --- | --- |
| SN | Questions | Answers |  |
|  | You or others in your household worried about not having enough food to eat because of a lack of money or other resources? | 1=Yes 2= No  98=Don’t know  97=Refused | \|__\|\|__\| |
|  | Still thinking about the last 12 MONTHS, was there a time when you or others in your household were unable to eat healthy and nutritious food because of a lack of money or other resources? | 1=Yes 2= No  98=Don’t know  97=Refused | \|__\|\|__\| |
|  | Was there a time when you or others in your household ate only a few kinds of foods because of a lack of money or other resources? | 1=Yes 2= No  98=Don’t know  97=Refused | \|__\|\|__\| |
|  | Was there a time when you or others in your household had to skip a meal because there was not enough money or other resources to get food? | 1=Yes 2= No  98=Don’t know  97=Refused | \|__\|\|__\| |
|  | Still thinking about the last 12 MONTHS, was there a time when you or others in your household ate less than you thought you should because of a lack of money or other resources? | 1=Yes 2= No  98=Don’t know  97=Refused | \|__\|\|__\| |
|  | Was there a time when your household ran out of food because of a lack of money or other resources? | 1=Yes 2= No  98=Don’t know  97=Refused | \|__\|\|__\| |
|  | Was there a time when you or others in your household were hungry but did not eat because there was not enough money or other resources for food? | 1=Yes 2= No  98=Don’t know  97=Refused | \|__\|\|__\| |
|  | Was there a time when you or others in your household went without eating for a whole day because of a lack of money or other resources? | 1=Yes 2= No  98=Don’t know  97=Refused | \|__\|\|__\| |
| During the last 12 months, was there a time when any of the children younger than 5 years old | | Skip if no children<5 |  |
|  | Did not eat healthy and nutritious foods because of a lack of money or other resources? | 1=Yes 2= No  98=Don’t know  97=Refused |  |
|  | Was not given enough food because of a lack of money or other resources? | 1=Yes 2= No  98=Don’t know  97=Refused |  |

##

## Nutritional and health status of Women of Reproductive Age (15-49 Years)

|  | Woman’s unique code |  |  |
| --- | --- | --- | --- |
|  | Woman’s age in years? | \|__\|\|__\| |  |
|  | What was your age at first marriage 🡪 if married in for marital status in section 1.2 | Year (specify) ______  98=Don’t know | \|__\|\|__\| |
|  | Have you ever been pregnant? | 1= Yes 2 = No **→Skip to 11** |  |
|  | What was your age at your first pregnancy | Year (specify)______  98=Don’t know **→ Skip to 11** | \|__\|\|__\| |
|  | Were you pregnant in the last 5 years? | 1 = Yes 2 =No **→Skip to 11** |  |
|  | Did you see anyone for antenatal care for the recent pregnancy? | 1 = Yes 2 = No **→Skip to 11** | \|__\| |
|  | How many times did you receive antenatal care during this pregnancy? | Number of times (specify)  98=Don’t know **→Skip to 11** | \|__\|\|__\| |
|  | During this pregnancy, did you practice fasting? | 1=Yes 2=No | \|__\|\|__\| |
|  | Are you currently pregnant? | 1=Yes 2=No **→Skip to 11**  98=Don't Know**→Skip to 11** |  |

## Child Nutritional and Health Status

| Now I would like to ask some questions about the health of your children born in the last 5 years. We will talk about each separately. | | | |
| --- | --- | --- | --- |
| **S.N** | **Question** | **Response** | **skip** |
|  | Child’s Unique Code |  | \|__\|\|__\| |
|  | Child (NAME) age?  *Age in months or age at the time of the child’s death* | _________ | \|__\|\|__\| |
|  | Is the child alive now? | 1=Yes 2=No **→ Skip to next module** |  |
|  | In the last six months, was (NAME) given any vitamin A supplement? | 1 =Yes 2=No 98=Don't know | \|__\|\|__\| |
|  | In the last 12 months, was (NAME) given any iron tablet or syrup or supplement? | 1 =Yes 2=No 98=Don't know | \|__\|\|__\| |
|  | In the last 6 months, was (NAME) given any medicine for intestinal worms? | 1 =Yes 2=No 98=Don't know | \|__\|\|__\| |
|  | In the last 6 months, what did the health care provider or community health worker talk with you about?  Anything else?  RECORD ALL MENTIONED. | 1=Breastfeeding 2=Not feeding water or other liquids before six months 3=Introducing food and liquids (other than Breast milk) when the baby reaches Six Months of age 4=Giving a variety of foods 5=Giving animal source foods specifically (e.g., eggs, milk, meat, fish)  6=How often to feed foods 7=Not feeding sugar-sweetened beverages  8=None |  |

## Infant and young child feeding practices

| **For children 0-23 months** | | | | | | | | | |
| --- | --- | --- | --- | --- | --- | --- | --- | --- | --- |
| This module is to be administered to the mother/caregiver of children born 0-23 months before the survey, living with respondents. Verify that the respondent you are speaking to is the mother/caregiver of the child. | | | | | | | | | |
| **Feeding immediately after birth:**  The respondents are women of reproductive age who have given birth (all live births) in the last two years. whether the child is **living or deceased** | | | | | | | | | |
|  | | Was (NAME) ever breastfed? | | 1=Yes  2=No **→ Skip to next module** | | | \|__\|\|__\| | | |
|  | | Was the child given the first milk (colostrum) after birth? | | 1=Yes  2=No  98=Don’t know | | |  | | |
|  | | How long after birth did you first put (NAME) to the breast?  If immediately, record “000”  If less than one hour, record “00” hours  If less than 24 hours, record hours  Otherwise, record days | | Immediately after birth \|__\|\|__\|\|__\|  hours \|__\|\|__\|  days \|__\|\|__\| | | | \|__\|\|__\| | | |
|  | | In the first **two days** after delivery, was [NAME] given anything other than breast milk to eat or drink – anything at all like water, infant formula, or [water with sugar…]? | | 1=Yes  2=No | | |  | | |
| **Current breast- and bottle feeding**:  Questions about current breast- and bottle feeding are asked for all **living** children under 24 months | | | | | | | | | |
|  | | Was [NAME] breastfed yesterday from sunrise until today sunrise? | | 1=Yes  2=No **→Skip to 7**  98=Don’t know **→ Skip to 7** | | | \|__\|\|__\| | | |
|  | | Did *(NAME)* drink anything from a bottle with a nipple yesterday during the day or night? | | 1=Yes  2=No  98=Don’t know | | | **\|__\|\|__\|** | | |
| **Liquids**  Questions about liquids should be asked for all living children aged under two years. | | | | | | | | | |
|  | Now, I would like to ask you about some liquids that *(NAME)* may have had yesterday from sunrise until today sunrise?  *Please tell me about all drinks, whether [NAME] had them at home, or somewhere else*. | | | Yesterday during the day or at night, did [NAME] have…?  1=Yes  2=No  98=Don’t know | | \|__\|\|__\| | | | |
|  |  | | Plain water | 1=Yes  2=No  98 = Don’t know | | \|__\|\|__\| | | | |
|  |  |  | Infant formula such as S-26, Baby Luck, Nan, Cerelac, Liptomil? | 1=Yes 2=No**→Skip to** **11**  98=Don’t know **→Skip to** **11** | | \|__\|\|__\| | | | |
|  |  |  | If “Yes”: How many times did [NAME] drink formula?  If 7 or more, record “7”  If number of times not known, record “98” | \|__\|\|__\| | |  | | | |
|  |  |  | Milk from animals, such as fresh, tinned or powdered milk? | 1=Yes 2=No **→ Skip to 14**  98=Don’t know **🡪 skip to 14** | | \|__\|\|__\| | | | |
|  |  |  | If “yes”: How many times did [NAME] drink milk? If 7 or more, record “7” If number of times not known, record “98” | \|__\|\|__\| | | \|__\|\|__\| | | | |
|  |  |  | If “yes”: Was the milk or were any of the milk drinks a sweet or flavored type of milk? | 1=Yes 2=No  98=Don’t know | |  | | | |
|  |  |  | Yogurt drink? Such as *Holland*… yogurt | 1=Yes 2=No **🡪 skip to 17**  98=Don’t know **🡪 skip to 17** | | \|__\|\|__\| | | | |
|  |  |  | If “yes”: How many times did [NAME] drink yogurt? If 7 or more, record “7” If number of times not known, record “98” | \|__\|\|__\| | |  | | | |
|  |  |  | If “yes”: Was the yogurt or were any of the yogurt drinks a sweet or flavored type of yogurt drink? | 1=Yes 2=No  98=Don’t know | | \|__\|\|__\| | | | |
|  |  |  | Chocolate-flavored drinks including those made from syrups or powders? | 1=Yes 2=No  98=Don’t know | | \|__\|\|__\| | | | |
|  |  |  | Fruit juice or fruit-flavored drinks including those made from syrups or powders? | 1=Yes 2=No  98=Don’t know | | \|__\|\|__\| | | | |
|  |  |  | Sodas, malt drinks, sports drinks or energy drinks? | 1=Yes 2=No  98=Don’t know | | \|__\|\|__\| | | | |
|  |  |  | Tea, coffee, or herbal drinks? | 1=Yes 2=No **🡪 skip to** **22**  98=Don’t know **🡪 skip to 22** | | \|__\|\|__\| | | | |
|  |  |  | If “yes”: Was the drink/ Were any of these drinks sweetened? | 1=Yes 2=No  98=Don’t know | |  | | | |
|  |  |  | Clear broth or clear soup or *Atmit*? | 1=Yes 2=No  98=Don’t know | | \|__\|\|__\| | | | |
|  |  |  | Any other liquids? | 1=Yes 2=No **🡪 skip to** **26**  98=Don’t know**🡪 skip to** **26** | | \|__\|\|__\| | | | |
|  |  | | If “yes”: what was the liquid or what were the liquids? | _____________ | |  | | | |
|  |  | | If “yes”: Was the drink or were any of these drinks sweetened? | 1=Yes 2=No  98=Don’t know | |  | | | |
| **Breast milk Substitute** | | | | | | | | | |
|  | In the past six months, have you heard or seen any promotion or advertising for infant formula or other milk targeted for babies? | | | 1=Yes  2=No **🡪 skip to** **28** | |  | | | |
|  | Where did you see or hear it?  Anywhere else?  RECORD ALL MENTIONED. | | | 1= Hospital  2= Other Health Facility  3= TV/Radio  4= Magazine/Newspaper  5= Internet/social media  6= Shop/Pharmacy  7= Billboard/Public Poster  99= Other (Specify) | |  | | | |
| **Foods:**  Questions about foods are asked for all living children under two years. | | | | | | | | | |
| **OPEN RECALL QUESTIONNAIRE FOR FOODS** | | | | | | | | | |
| Now I would like to ask you about everything that [NAME] ate yesterday during the day or the night. I am interested in foods your child ate whether at home or somewhere else.  Think about when [NAME] woke up yesterday. Did (he/ she) eat anything at that time?  If “yes” ask: Please tell me everything [NAME] ate at that time.  Probe: Anything else? Record answers using the food groups below.  What did [NAME] do after that? Did he/she eat anything at that time?  Repeat this series of questions, recording in the food groups, until the respondent tells you that the child woke up this morning.  If a mixed dish is mentioned: Probe: What were the main ingredients in [MIXED DISH]?  Record answers in the correct food groups **28-45.**  Yesterday during the day or at night, did (NAME) eat: | | | | | | | | | |
| **Second pass** | | For each food group **not** mentioned after completing the above, ask: Just to make sure, did [NAME] eat [FOOD GROUP ITEMS] yesterday during the day or the night? | | | | | | Response Code | Skip |
|  | | | | | 1=Yes 2=No 98= Don’t know | | | |  |
|  | | Yogurt, other than yogurt drink? | | | | | | \|__\|\|__\| |  |
|  | | If “yes”: How many times did [NAME] eat yogurt? If more than 7, record “7” If number of times not known, record “98” | | | | | | \|__\|\|__\| |  |
|  | | Rice, pasta, macaroni, or commercial white bread? | | | | | |  |  |
|  | | Enjera, homemade bread, kita, chechebassa, nifiro, qinche, porridge, maize, or barley? | | | | | | \|__\|\|__\| |  |
|  | | Pumpkin, carrots, squash, or sweet potatoes that are yellow or orange inside? | | | | | | \|__\|\|__\| |  |
|  | | White potatoes, white yams, bulla, kocho, manioc, cassava or any other foods made from roots? | | | | | | \|__\|\|__\| |  |
|  | | Mitil gamfo, kik, shiro, ful, ashuk, niforo from beans, eshset from beans, chickpeas, or peas? | | | | | |  |  |
|  | | Carrots, pumpkin, or sweet potatoes that are orange inside? | | | | | |  |  |
|  | | Ethiopian kale, Swiss chard, broccoli, spinach, moringa leaves, cassava leaves, or sweet potato leaves? | | | | | |  |  |
|  | | Tomatoes, eggplant, beetroot, zucchini, or head cabbage? | | | | | |  |  |
|  | | Cucumber, lettuce, green pepper, cauliflower, or celery? | | | | | |  |  |
|  | | Mango or papaya? | | | | | | \|__\|\|__\| |  |
|  | | Orange, mandarin, or grapefruit? | | | | | | \|__\|\|__\| |  |
|  | | Banana, avocado, pineapple, apple, or watermelon? | | | | | | \|__\|\|__\| |  |
|  | | Guava, dates, prickly pear, strawberries, prim, or peaches? | | | | | | \|__\|\|__\| |  |
|  | | Cakes, cookies, biscuits, sweet breads, baklava, mushebek, or bombolino? | | | | | |  |  |
|  | | Ice cream, candy, or chocolates? | | | | | |  |  |
|  | | Eggs? | | | | | |  |  |
|  | | Cheese, cottage cheese, or feta? | | | | | |  |  |
|  | | Dulet, liver, kidney, heart, lung, or gizzard? | | | | | | \|__\|\|__\| |  |
|  | | Sausages, canned meat, or kuanta? | | | | | | \|__\|\|__\| |  |
|  | | Beef, sheep, goat, or raw meat? | | | | | | \|__\|\|__\| |  |
|  | | Pork or camel? | | | | | | \|__\|\|__\| |  |
|  | | Chicken? | | | | | | \|__\|\|__\| |  |
|  | | Fish, dried fish, tuna, or canned fish? | | | | | | \|__\|\|__\| |  |
|  | | Milk and milk products such as: cottage cheese, cheese (hard or soft) or other food made from milk? | | | | | | \|__\|\|__\| |  |
|  | | Groundnuts, peanut butter, selit, suf fitfit, suf water, or kolo with nuts or with suf? | | | | | |  |  |
|  | | Potato chips? | | | | | | \|__\|\|__\| |  |
|  | | Indomie? | | | | | | \|__\|\|__\| |  |
|  | | Chips, fried dough, samosa, spring rolls, or deep-fried vegetables? | | | | | |  |  |
|  | | Other solid, semi-solid or soft foods?  List all other solid, semi-solid or soft foods that do not fit food groups **28-45** here | | | | | | \|__\|\|__\| |  |
|  | | Yesterday, did [NAME] eat food from any place like hotel, restorant etc,  Chicken Hut, Pizza Hut, Wow burger, In-and-Out, Kaldis burger, or any burger cafe? | | | | | | 1=Yes  2=No  98= Don’t know | |
|  | | How many times did [NAME] eat any solid, semi-solid or soft foods yesterday during the day or night? If 7 or more times, record “7”. If number of times not known, record “98” | | | | | | Number of times  \|__\|\|__\| | |

## Diet Quality Questionnaire (DQQ) for Children (6-23 Months old)

| 1 | Was [NAME] ever breastfed? | 1=Yes 2=No | DON'T KNOW (DK) |
| --- | --- | --- | --- |
| 2 | How long after birth was [NAME] first put to the breast? | 1= Immediately  2= Within in an hour  3= With in less than 24 hours  4= Otherwise፣ record days |  |
| 3 | In the first 2 days after delivery፣ was [NAME] given anything other than breastmilk to eat or drink –anything at all like water፣ formula፣ herbal drinks፣ tena adam፣ fenugreek water፣ sugar water፣ butter? | 1=Yes 2=No | DK |
| 4 | Was [NAME] breastfed yesterday during the day or at night? | 1=Yes 2=No | DK |
| 5 | Did [NAME] drink anything from a bottle with a nipple yesterday during the day or at night? | 1=Yes 2=No | DK |
| 6 | Now I would like to ask you about liquids that [NAME] may have had yesterday during the day or at night.  Please tell me about all drinks፣ whether [NAME] had them at home፣ or somewhere else.  Yesterday during the day or at night፣ did [NAME] have... |  | |
| 6A | Plain water? | 1=Yes 2=No | DK |
| 6B | Formula፣ such as Baby Luck፣ Nan፣ Cerelac፣ S-26፣ or Liptomil? | 1=Yes 2=No | DK |
| 6Bnum | IF YES: How many times did (NAME) drink infant formula? (IF 7 OR MORE TIMES፣ RECORD '7'). | # | DK |
| 6C.25 | Milk from animals፣ including fresh or packaged? | 1=Yes 2=No | DK |
| 6Cnum | IF YES: How many times did (NAME) drink milk? (IF 7 OR MORE TIMES፣ RECORD '7'). | # | DK |
| 6Cswt.26 | IF YES: Was any of the milk a sweet or flavoured type of milk? | 1=Yes 2=No | DK |
| 6K | N/A | 1=Yes 2=No | DK |
| 6Kswt | N/A | 1=Yes 2=No | DK |
| 6E | N/A | 1=Yes 2=No | DK |
| 6F.27 | Fresh fruit juice፣ packed fruit juice or fruit drinks? | 1=Yes 2=No | DK |
| 6G.28 | Leslassa such as Coke፣ Fanta፣ Sprite፣ Sofi Malt፣ or Malta Guinness? | 1=Yes 2=No | DK |
| 6H | Tea፣ coffee፣ or herbal drinks? | 1=Yes 2=No | DK |
| 6Hswt.26 | IF YES: was the drink sweetened? | 1=Yes 2=No | DK |
| 6I | Clear broth or clear soup? | 1=Yes 2=No | DK |
| 6J | Any other liquids? | 1=Yes 2=No | DK |
|  | IF YES: What was the liquid or what were the liquids? |  |  |
| 6Jswt | IF YES: Was the drink sweetened? | 1=Yes 2=No | DK |
| 7 | Now I would like to ask you about foods that [NAME] had yesterday during the day or at night. I am interested in foods your child ate whether at home or somewhere else. Please think about snacks and small meals as well as main meals.  I will ask you about different types of foods፣ and I would like to know whether your child ate the food even if it was combined with other foods. Please do not answer ‘yes’ for any food or ingredient used in a small amount to add flavour to a dish.  Yesterday during the day or at night፣ did [NAME] eat: |  | |
| 7.15 | Yogurt? | 1=Yes 2=No | DK |
| 7.15num | IF YES: How many times did (NAME) have yogurt? | # | DK |
| 6D | IF YES: Did (NAME) have any yogurt as a drink? | 1=Yes 2=No | DK |
| 6Dswt | IF YES: Was it a sweet or flavored type of drink? | 1=Yes 2=No | DK |
|  | Yesterday፣ did [NAME] eat any of the following foods: |  |  |
| 7.1 | Rice፣ pasta፣ macaroni፣ or commercial white bread? | 1=Yes 2=No | DK |
| 7.2 | Enjera፣ homemade bread፣ kita፣ chechebassa፣ nifiro፣ qinche፣ porridge፣ maize፣ or barley? | 1=Yes 2=No | DK |
| 7.3 | Potato፣ sweet potato፣ any food from enset፣ yam፣ anchote፣ cassava፣ or taro? | 1=Yes 2=No | DK |
| 7.4 | TRT- | 1=Yes 2=No | DK |
|  | Yesterday፣ did [NAME] eat any of the following vegetables |  |  |
| 7.5 | Carrots፣ pumpkin፣ or sweet potatoes that are orange inside? | 1=Yes 2=No | DK |
| 7.6 | Ethiopian kale፣ Swiss chard፣ broccoli፣ spinach፣ moringa leaves፣ cassava leaves፣ or sweet potato leaves? | 1=Yes 2=No | DK |
| 7.7.1 | Tomatoes፣ eggplant፣ beetroot፣ zucchini፣ or head cabbage? | 1=Yes 2=No | DK |
| 7.7.2 | Cucumber፣ lettuce፣ green pepper፣ cauliflower፣ or celery? | 1=Yes 2=No | DK |
|  | Yesterday፣ did [NAME] eat any of the following fruits: |  |  |
| 7.8 | Mango or papaya? | 1=Yes 2=No | DK |
| 7.9 | Orange፣ mandarin፣ or grapefruit? | 1=Yes 2=No | DK |
| 7.10.1 | Banana፣ avocado፣ pineapple፣ apple፣ or watermelon? | 1=Yes 2=No | DK |
| 7.10.2 | Guava፣ dates፣ prickly pear፣ prim፣ or peaches? | 1=Yes 2=No | DK |
|  | Yesterday፣ did [NAME] eat any of the following sweets: | 1=Yes 2=No | DK |
| 7.11 | Cakes፣ cookies፣ biscuits፣ sweet breads፣ baklava፣ mushebek፣ or bombolino? | 1=Yes 2=No | DK |
| 7.12 | Ice cream፣ candy፣ or chocolates? | 1=Yes 2=No | DK |
|  | Yesterday፣ did [NAME] eat any of the following foods of animal origin: |  |  |
| 7.13 | Eggs? | 1=Yes 2=No | DK |
| 7.14 | Cheese፣ cottage cheese፣ or feta? | 1=Yes 2=No | DK |
| 7org | Dulet፣ liver፣ kidney፣ heart፣ lung፣ or gizzard? | 1=Yes 2=No | DK |
| 7.16 | Sausages፣ canned meat፣ or kuanta? | 1=Yes 2=No | DK |
| 7.17 | Beef፣ sheep፣ goat፣ or raw meat? | 1=Yes 2=No | DK |
| 7.18 | Pork or camel? | 1=Yes 2=No | DK |
| 7.19 | Chicken? | 1=Yes 2=No | DK |
| 7.20 | Fish፣ dried fish፣ tuna፣ or canned fish? | 1=Yes 2=No | DK |
| 7insect | N/A | 1=Yes 2=No | DK |
|  | Yesterday፣ did [NAME] eat any of the following other foods: | 1=Yes 2=No | DK |
| 7.21 | Groundnuts፣ peanut butter፣ selit፣ suf fitfit፣ suf water፣ or kolo with nuts or with suf? | 1=Yes 2=No | DK |
| 7.22 | Potato chips? | 1=Yes 2=No | DK |
| 7.23 | Indomie? | 1=Yes 2=No | DK |
| 7.24 | Chips፣ fried dough፣ samosa፣ spring rolls፣ or deep-fried vegetables? | 1=Yes 2=No | DK |
| 7red | N/A | 1=Yes 2=No | DK |
| 7R | Any other solid፣ semi-solid፣ or soft food? | 1=Yes 2=No | DK |
|  | IF YES: What was the food? | 1=Yes 2=No | DK |
|  | Yesterday፣ did [NAME] eat food from any place like... | 1=Yes 2=No | DK |
| 7.29 | Chicken Hut፣ Pizza Hut፣ Wow burger፣ In-and-Out፣ Kaldis burger፣ or any burger cafe? | 1=Yes 2=No | DK |
| CHECK  ማረጋገጫ | Note for interviewer: If not a single “yes” for foods is recorded፣ ask 7S. |  | |
|  | If at least one “yes” for foods፣ skip to 8. |  |  |
| 7S | Did [NAME] eat any solid፣ semi-solid፣ or soft food yesterday during the day or night? | 1=Yes 2=No | DK |
| 8 | How many times did [NAME] eat any solid፣ semi-solid or soft foods yesterday during the day or night?  If 7 or more times፣ record “7 | 1=Yes 2=No | DK |

## Diet Quality Questionnaire (DQQ) for Children (2-5 years old)

Read: Now I’d like to ask you some yes-or-no questions about foods and drinks that your child consumed yesterday during the day or night, whether he/she had it at home or somewhere else.

First, I would like you to think about yesterday, from the time your child woke up through the night. Think to yourself about the first thing you ate or drank after you woke up in the morning … Think about where he/she were when you had any food or drink in the middle of the day … Think about where he/she were when you had any evening meal … and any food or drink he/she may have had in the evening or late-night... and any other snacks or drinks he/she may have had between meals throughout the day or night.

I am interested in whether you had the food items I will mention even if they were combined with other foods.

Please listen to the list of foods and drinks, and if you ate or drank ANY ONE OF THEM, say yes.

|  | **Yesterday, did you eat any of the following foods:** | **CODE** |
| --- | --- | --- |
| 1 | Rice, pasta, macaroni, or commercial white bread? | 1=Yes 2=No |
| 2 | Enjera, homemade bread, kita, chechebassa, nifiro, qinche, porridge, maize, or barley፣ Kolo from grains, chiko, or beso? | 1=Yes 2=No |
| 3 | Potato, sweet potato, any food from enset, yam, anchote, cassava, or taro? | 1=Yes 2=No |
| 4 | Kik, shiro, ful, helbet, siljo, ashuk, nifro from beans, kolo from beans, eshet from beans, chickpeas, or peas? | 1=Yes 2=No |
|  | **Yesterday, did you eat any of the following vegetables:** |  |
| 5 | Carrots, pumpkin, or sweet potatoes that are orange inside? | 1=Yes 2=No |
| 6 | Ethiopian kale, Swiss chard, broccoli, spinach, moringa leaves, cassava leaves, or sweet potato leaves? | 1=Yes 2=No |
| 7.1 | Tomatoes, eggplant, beetroot, zucchini, or head cabbage? | 1=Yes 2=No |
| 7.2 | Cucumber, lettuce, green pepper, cauliflower, or celery? | 1=Yes 2=No |
|  | **Yesterday, did you eat any of the following fruits:** |  |
| 8 | Mango or papaya? | 1=Yes 2=No |
| 9 | Orange, mandarin, or grapefruit? | 1=Yes 2=No |
| 10.1 | Banana, avocado, pineapple, apple, or watermelon? | 1=Yes 2=No |
| 10.2 | Guava, dates, prickly pear, strawberries, prim, or peaches? | 1=Yes 2=No |
|  | **Yesterday, did you eat any of the following sweets:** |  |
| 11 | Cakes, cookies, biscuits, sweet breads, baklava, mushebek, or bombolino? | 1=Yes 2=No |
| 12 | Ice cream, candy, or chocolates? | 1=Yes 2=No |
|  | **Yesterday, did you eat any of the following foods of animal origin:** |  |
| 13 | Eggs? | 1=Yes 2=No |
| 14 | Cheese, cottage cheese, or feta? | 1=Yes 2=No |
| 15 | Yogurt? | 1=Yes 2=No |
| 16 | Sausages, canned meat, or kuanta? | 1=Yes 2=No |
| 17 | Beef, sheep, goat, or raw meat? | 1=Yes 2=No |
| 18 | Pork or camel? | 1=Yes 2=No |
| 19 | Chicken? | 1=Yes 2=No |
| 20 | Fish, dried fish, tuna, or canned fish? | 1=Yes 2=No |
|  | **Yesterday, did you eat any of the following other foods:** |  |
| 21 | Groundnuts, peanut butter, selit, suf fitfit, suf water, or kolo with nuts or with suf? | 1=Yes 2=No |
| 22 | Potato chips? | 1=Yes 2=No |
| 23 | Indomie? | 1=Yes 2=No |
| 24 | Chips, fried dough, samosa, spring rolls, or deep fried vegetables? | 1=Yes 2=No |
|  | **Yesterday, did you have any of the following beverages:** | 1=Yes 2=No |
| 25 | Dairy milk, milk powder, or milk in tea? | 1=Yes 2=No |
| 26 | Tea with sugar, coffee with sugar, sprisse with sugar, macchiato, or milk with sugar? | 1=Yes 2=No |
| 27 | Fresh fruit juice, packed fruit juice or fruit drinks? | 1=Yes 2=No |
| 28 | Sweet beverages (Leslassa) such as Coke, Fanta, Sprite, Sofi Malt, or Malta Guinness? | 1=Yes 2=No |
|  | **Yesterday, did you get food from any place like...** |  |
| 29 | Chicken Hut, Pizza Hut, Wow burger, In-and-Out, Kaldis burger, or any burger cafe? | 1=Yes 2=No |

## Women empowerment

| **Sr. No** | **Question** | **Response** |
| --- | --- | --- |
|  | Have you done any work in last 12 months? | 0=No; In the past year**→ Skip to 4**  1=Have a job, but on leave last 7 days  2= Currently working |
|  | What is your occupation? That is, what kind of work do you mainly do? | ____________ |
|  | Are you paid in cash or kind for this work or are you not paid at all? | 1= Cash only  2= Cash and kind  3= In kind only  4= Not paid |
|  | How often do you read a newspaper or magazine: almost every day, at least once a week, less than once a week or not at all? | 1= at least once a week  2= less than once a week  3= not at all |
|  | Who usually decides how the money you earn will be used? | 1= Mainly myself  2= Mainly husband/partner  3 =Joint decision  99 = Other specify |
|  | Who usually decides how your (husband's/partner's) earnings will be used: you, your (husband/partner), or you and your (husband/partner) jointly? | 1= Myself  2= husband/partner  3 =Joint decision  4= husband/partner has no earning  99 = Other specify |
|  | Who usually makes decisions about health care for yourself? | 1= Mainly myself  2 = Mainly husband/partner  3 =Joint decision  4= Someone else  99=Other |
|  | Who usually makes decisions about making major household purchases? | 1=Mainly myself  2=Mainly husband/partner  3=Joint decision  4=Someone else  99=Other specify |
|  | Who usually makes decisions about visits to your family or relatives? | 1= Mainly myself  2 = Mainly husband/partner  3 =Joint decision  4= Someone else  99= Other specify |
|  | Who usually makes decisions about your child health care? | 1= Mainly myself  2 = Mainly husband/partner  3 =Joint decision  4= Someone else  99= Other specify (have no children) |
|  | Do you own this or any other house either alone or jointly with someone else? | 1= Alone  2= Jointly with husband/partner  3= Jointly with someone else  4= Jointly with husband/partner and someone else  5= Both alone and jointly  6= Does not own |
|  | Do you own any agricultural or non-agricultural land either alone or jointly with someone else? | 1= Alone  2= Jointly with husband/partner  3= Jointly with someone else  4= Jointly with husband/partner and someone else  5= Both alone and jointly  6= Does not own |
|  | In your opinion, is a husband justified in hitting or beating his wife in the following situations  A= if she goes out without telling him  B= if she neglects the children  C= if she argues with him  D= if she refused having sex with him  E= if she burns the food | 1=Yes  2=No  98=Don’t know  A= goes out  B= neglects the children  C= argues  D= refused sex  E= burns food |

## Diet Quality Questionnaire (DQQ) for Women

Read: Now I’d like to ask you some yes-or-no questions about foods and drinks that you consumed yesterday during the day or night, whether you had it at home or somewhere else.

First, I would like you to think about yesterday, from the time you woke up through the night. Think to yourself about the first thing you ate or drank after you woke up in the morning … Think about where you were when you had any food or drink in the middle of the day … Think about where you were when you had any evening meal … and any food or drink you may have had in the evening or late-night... and any other snacks or drinks you may have had between meals throughout the day or night.

I am interested in whether you had the food items I will mention even if they were combined with other foods.

Please listen to the list of foods and drinks, and if you ate or drank ANY ONE OF THEM, say yes.

|  | **Yesterday, did you eat any of the following foods:** | **(Circles answer)** |
| --- | --- | --- |
| 1 | Rice, pasta, macaroni, or commercial white bread? | 1=Yes 2=No |
| 2 | Enjera, homemade bread, kita, chechebassa, nifiro, qinche, porridge, maize, or barley? | 1=Yes 2=No |
| 3 | Potato, sweet potato, any food from enset, yam, anchote, cassava, or taro? | 1=Yes 2=No |
| 4 | Kik, shiro, ful, helbet, siljo, ashuk, nifro from beans, kolo from beans, eshet from beans, chickpeas, or peas? | 1=Yes 2=No |
|  | **Yesterday, did you eat any of the following vegetables:** | 1=Yes 2=No |
| 5 | Carrots, pumpkin, or sweet potatoes that are orange inside? | 1=Yes 2=No |
| 6.1 | Ethiopian kale, Swiss chard, broccoli, spinach, moringa leaves, cassava leaves, or sweet potato leaves? | 1=Yes 2=No |
| 7.1 | Tomatoes, eggplant, beetroot, zucchini, or head cabbage? | 1=Yes 2=No |
| 7.2 | Cucumber, lettuce, green pepper, cauliflower, or celery? | 1=Yes 2=No |
|  | **Yesterday, did you eat any of the following fruits:** |  |
| 8 | Mango or papaya? | 1=Yes 2=No |
| 9 | Orange, mandarin, or grapefruit? | 1=Yes 2=No |
| 10.1 | Banana, avocado, pineapple, apple, or watermelon? | 1=Yes 2=No |
| 10.2 | Guava, dates, prickly pear, strawberries, prim, or peaches? | 1=Yes 2=No |
|  | **Yesterday, did you eat any of the following sweets:** | 1=Yes 2=No |
| 11 | Cakes, cookies, biscuits, sweet breads, baklava, mushebek, or bombolino? | 1=Yes 2=No |
| 12 | Ice cream, candy, or chocolates? | 1=Yes 2=No |
|  | **Yesterday, did you eat any of the following foods of animal origin:** | 1=Yes 2=No |
| 13 | Eggs? | 1=Yes 2=No |
| 14 | Cheese, cottage cheese, or feta? | 1=Yes 2=No |
| 15 | Yogurt? | 1=Yes 2=No |
| 16 | Sausages, canned meat, or kuanta? | 1=Yes 2=No |
| 17 | Beef, sheep, goat, or raw meat? | 1=Yes 2=No |
| 18 | Pork or camel? | 1=Yes 2=No |
| 19 | Chicken? | 1=Yes 2=No |
| 20 | Fish, dried fish, tuna, or canned fish? | 1=Yes 2=No |
|  | **Yesterday, did you eat any of the following other foods:** | 1=Yes 2=No |
| 21 | Groundnuts, peanut butter, selit, suf fitfit, suf water, or kolo with nuts or with suf? | 1=Yes 2=No |
| 22 | Potato chips? | 1=Yes 2=No |
| 23 | Indomie? | 1=Yes 2=No |
| 24 | Chips, fried dough, samosa, spring rolls, or deep-fried vegetables? | 1=Yes 2=No |
|  | **Yesterday, did you have any of the following beverages:** | 1=Yes 2=No |
| 25 | Dairy milk, milk powder, or milk in tea? | 1=Yes 2=No |
| 26 | Tea with sugar, coffee with sugar, sprisse with sugar, macchiato, or milk with sugar? | 1=Yes 2=No |
| 27 | Fresh fruit juice, packed fruit juice or fruit drinks? | 1=Yes 2=No |
| 28 | Sweet beverages (Leslassa) such as Coke, Fanta, Sprite, Sofi Malt, or Malta Guinness? | 1=Yes 2=No |
|  | Yesterday, did you get food from any place like... | 1=Yes 2=No |
| 29 | Chicken Hut, Pizza Hut, Wow burger, In-and-Out, Kaldis burger, or any burger cafe? | 1=Yes 2=No |

## Adolescent girls (10-19 Years)

| Hint: This section is administered for Adolescent girls 10-19 years old. Provide a paper copy of both the informed consent and Assent Form to the respondent Read the consent (for mothers of adolescent girls) and Assent (adolescent girls) form | | | |
| --- | --- | --- | --- |
|  | Girl’s unique code |  | \|__\|\|__\| |
|  | Are you currently a student? | 1=Yes  2=No | **\|__\|** |
|  | Were you given any iron/folate tablets at school or out of school? (***Show the tablet)*** | 1=Yes  2=No | **\|__\|** |
|  | How many weeks per month have you taken the iron tablets? | Weeks per month (specify)  98=Don’t know | **\|__\|\|__\|** |
|  | Were you given any drug for intestinal worms at school or out of school in the last six months? | 1=Yes  2=No | **\|__\|** |
|  | Have you received any nutrition counseling in the last six months? | 1=Yes  2=No | **\|__\|** |
|  | Did you receive nutritional assessment services in health facilities when you went for any kind of health service? | 1=Yes  2=No | **\|__\|** |
|  | Is there any food taboo for adolescent girls in your community? | 1=Yes  2=No | **\|__\|** |
|  | Mention types of food taboo? |  | |

##

## Diet Quality Questionnaire (DQQ) for Adolescent girls (10-19 Years)

Read: Now I’d like to ask you some yes-or-no questions about foods and drinks that you consumed yesterday during the day or night, whether you had it at home or somewhere else.

First, I would like you to think about yesterday, from the time you woke up through the night. Think to yourself about the first thing you ate or drank after you woke up in the morning … Think about where you were when you had any food or drink in the middle of the day … Think about where you were when you had any evening meal … and any food or drink you may have had in the evening or late-night... and any other snacks or drinks you may have had between meals throughout the day or night.

I am interested in whether you had the food items I will mention even if they were combined with other foods.

Please listen to the list of foods and drinks, and if you ate or drank ANY ONE OF THEM, say yes.

|  | Yesterday, did you eat any of the following foods: | (Circles answer) |
| --- | --- | --- |
| 1 | Rice, pasta, macaroni, or commercial white bread? | YES or NO |
| 2 | Enjera, homemade bread, kita, chechebassa, nifiro, qinche, porridge, maize, or barley? | YES or NO |
| 3 | Potato, sweet potato, any food from enset, yam, anchote, cassava, or taro? | YES or NO |
| 4 | Kik, shiro, ful, helbet, siljo, ashuk, nifro from beans, kolo from beans, eshet from beans, chickpeas, or peas? | YES or NO |
|  | **Yesterday, did you eat any of the following vegetables:** |  |
| 5 | Carrots, pumpkin, or sweet potatoes that are orange inside? | YES or NO |
| 6.1 | Ethiopian kale, Swiss chard, broccoli, spinach, moringa leaves, cassava leaves, or sweet potato leaves? | YES or NO |
| 7.1 | Tomatoes, eggplant, beetroot, zucchini, or head cabbage? | YES or NO |
| 7.2 | Cucumber, lettuce, green pepper, cauliflower, or celery? | YES or NO |
|  | **Yesterday, did you eat any of the following fruits:** |  |
| 8 | Mango or papaya? | YES or NO |
| 9 | Orange, mandarin, or grapefruit? | YES or NO |
| 10.1 | Banana, avocado, pineapple, apple, or watermelon? | YES or NO |
| 10.2 | Guava, dates, prickly pear, strawberries, prim, or peaches? | YES or NO |
|  | **Yesterday, did you eat any of the following sweets:** |  |
| 11 | Cakes, cookies, biscuits, sweet breads, baklava, mushebek, or bombolino? | YES or NO |
| 12 | Ice cream, candy, or chocolates? | YES or NO |
|  | **Yesterday, did you eat any of the following foods of animal origin:** |  |
| 13 | Eggs? | YES or NO |
| 14 | Cheese, cottage cheese, or feta? | YES or NO |
| 15 | Yogurt? | YES or NO |
| 16 | Sausages, canned meat, or kuanta? | YES or NO |
| 17 | Beef, sheep, goat, or raw meat? | YES or NO |
| 18 | Pork or camel? | YES or NO |
| 19 | Chicken? | YES or NO |
| 20 | Fish, dried fish, tuna, or canned fish? | YES or NO |
|  | **Yesterday, did you eat any of the following other foods:** |  |
| 21 | Groundnuts, peanut butter, selit, suf fitfit, suf water, or kolo with nuts or with suf? | YES or NO |
| 22 | Potato chips? | YES or NO |
| 23 | Indomie? | YES or NO |
| 24 | Chips, fried dough, samosa, spring rolls, or deep-fried vegetables? | YES or NO |
|  | **Yesterday, did you have any of the following beverages:** |  |
| 25 | Dairy milk, milk powder, or milk in tea? | YES or NO |
| 26 | Tea with sugar, coffee with sugar, sprisse with sugar, macchiato, or milk with sugar? | YES or NO |
| 27 | Fresh fruit juice, packed fruit juice or fruit drinks? | YES or NO |
| 28 | Sweet beverages (Leslassa) such as Coke, Fanta, Sprite, Sofi Malt, or Malta Guinness? | YES or NO |
|  | **Yesterday, did you get food from any place like...** |  |
| 29 | Chicken Hut, Pizza Hut, Wow burger, In-and-Out, Kaldis burger, or any burger cafe? | YES or NO |

## Dietary Intake (FFQ) for Caregiver/Women

|  | Food frequency questionnaire answered by the mothers  **Instruction:** For each food item listed below, indicate with a checkmark (√) the category that best describes the frequency with which you usually eat that particular food item. Thinking about the last three months, how frequently did you eat the following food items within per week during the last one months and one year | | | | | | | | | | | | | | | | | |  |
| --- | --- | --- | --- | --- | --- | --- | --- | --- | --- | --- | --- | --- | --- | --- | --- | --- | --- | --- | --- |
|  |  |  |  |  |  |  |  |  |  | |  | |  | |  | |  | | |
|  | Food item | Once or more than once per day | 3-6 times per week | Once or twice per week | Twice per month or less | never |  | Food item | Once or more than once per day | | 3-6 times per week | | Once or twice per week | | Twice per month or less | | never | |  |
|  |  |  |  |  |  |  |  |  |  | |  | |  | |  | |  | |  |
|  | Orange |  |  |  |  |  |  | Avocado |  | |  | |  | |  | |  | | |
|  | Banana |  |  |  |  |  |  | Carrot |  | |  | |  | |  | |  | | |
|  | Mango |  |  |  |  |  |  | pumpkin |  | |  | |  | |  | |  | | |
|  | Tomato |  |  |  |  |  |  | Sweet potatoes |  | |  | |  | |  | |  | | |
|  | Potatoes |  |  |  |  |  |  | Sorghum |  | |  | |  | |  | |  | | |
|  | Beetroot |  |  |  |  |  |  | Maize |  | |  | |  | |  | |  | | |
|  | Cabbage/kale |  |  |  |  |  |  | Teff |  | |  | |  | |  | |  | | |
|  | Papaya |  |  |  |  |  |  | Cheese/cottage cheese |  | |  | |  | |  | |  | | |
|  | Lentils |  |  |  |  |  |  | Butter |  | |  | |  | |  | |  | | |
|  | Chickpeas |  |  |  |  |  |  | Whey |  | |  | |  | |  | |  | | |
|  | Lettuce |  |  |  |  |  |  | Yogurt |  | |  | |  | |  | |  | | |
|  | Haricot beans |  |  |  |  |  |  | Chicken |  | |  | |  | |  | |  | | |
|  | Enset |  |  |  |  |  |  | Eggs |  | |  | |  | |  | |  | | |
|  | Beans/ broad bean |  |  |  |  |  |  | Fish |  | |  | |  | |  | |  | | |
|  | Wheat |  |  |  |  |  |  | Beef | |  | |  | |  | |  | |  | |
|  | Barley |  |  |  |  |  |  | Milk | |  | |  | |  | |  | |  | |
|  | Millet |  |  |  |  |  |  | Rice | |  | |  | |  | |  | |  | |
|  | Moringa |  |  |  |  |  |  | Guava | |  | |  | |  | |  | |  | |

## Dietary Intake (FFQ) for Children (6-59 Months)

|  | Food frequency questionnaire answered by the mothers  **Instruction:** For each food item listed below, indicate with a checkmark (√) the category that best describes the frequency with which the child usually eat that particular food item. Thinking about the last three months, how frequently did your child eat the following food items within per week during the last one months and one year | | | | | | | | | | | | | | | | | |  |
| --- | --- | --- | --- | --- | --- | --- | --- | --- | --- | --- | --- | --- | --- | --- | --- | --- | --- | --- | --- |
|  |  |  |  |  |  |  |  |  |  | |  | |  | |  | |  | | |
|  | Food item | Once or more than once per day | 3-6 times per week | Once or twice per week | Twice per month or less | never |  | Food item | Once or more than once per day | | 3-6 times per week | | Once or twice per week | | Twice per month or less | | never | |  |
|  |  |  |  |  |  |  |  |  |  | |  | |  | |  | |  | |  |
|  | Orange |  |  |  |  |  |  | Avocado |  | |  | |  | |  | |  | | |
|  | Banana |  |  |  |  |  |  | Carrot |  | |  | |  | |  | |  | | |
|  | Mango |  |  |  |  |  |  | pumpkin |  | |  | |  | |  | |  | | |
|  | Tomato |  |  |  |  |  |  | Sweet potatoes |  | |  | |  | |  | |  | | |
|  | Potatoes |  |  |  |  |  |  | Sorghum |  | |  | |  | |  | |  | | |
|  | Beetroot |  |  |  |  |  |  | Maize |  | |  | |  | |  | |  | | |
|  | Cabbage/kale |  |  |  |  |  |  | Teff |  | |  | |  | |  | |  | | |
|  | Papaya |  |  |  |  |  |  | Cheese/cottage cheese |  | |  | |  | |  | |  | | |
|  | Lentils |  |  |  |  |  |  | Butter |  | |  | |  | |  | |  | | |
|  | Chickpeas |  |  |  |  |  |  | Whey |  | |  | |  | |  | |  | | |
|  | Lettuce |  |  |  |  |  |  | Yogurt |  | |  | |  | |  | |  | | |
|  | Haricot beans |  |  |  |  |  |  | Chicken |  | |  | |  | |  | |  | | |
|  | Enset |  |  |  |  |  |  | Eggs |  | |  | |  | |  | |  | | |
|  | Beans/ broad bean |  |  |  |  |  |  | Fish |  | |  | |  | |  | |  | | |
|  | Wheat |  |  |  |  |  |  | Beef | |  | |  | |  | |  | |  | |
|  | Barley |  |  |  |  |  |  | Milk | |  | |  | |  | |  | |  | |
|  | Millet |  |  |  |  |  |  | Rice | |  | |  | |  | |  | |  | |
|  | Moringa |  |  |  |  |  |  | Guava | |  | |  | |  | |  | |  | |

# **Module Six****: Consumer behavior**

## Knowledge, Attitudes and Motives

### Knowledge

| 1. | How long after birth should a baby start breastfeeding? | 1=Immediately, within 1 hour of delivery  2= Some hours later but within 24 hours  3=After 1 day  4=After 2 days  5=After 3 days  6=Do not think a baby should be breastfed  98=Don’t know |
| --- | --- | --- |
| 2. | How long should a baby receive nothing other than breast milk? | 1= From birth to six months  98= Don’t know  99= Other specify |
| 3. | How often should a baby younger than six months be breastfed? | 1= On demand, whenever the baby wants  98= Don’t know  99= Other specify |
| 4. | How much should a child be fed when he/she is sick? | 1=Less than usual  2=Same as usual  3=More than usual  98=Don’t know |
| 5. | How often should a child be fed when he/she is sick? | 1=Less than usual  2=Same as usual  3=More than usual  98=Don’t know |
| 6 | What should you do (in relation to feeding) AFTER a child has recovered from diarrhea or other illness?  (MULTIPLE RESPONSES POSSIBLE) | 1=Feed less than usual  2=Feed as much food as usual  3=Feed more than usual  4=Feed an extra meal every day for 2 weeks  5=Give more liquids than usual  6=Continue breastfeeding  99=Other specify |
| 7 | Have you ever heard of child stunting? | 1=Yes  2=No **if no skip to 10** |
| 8 | At what age are children at highest risk of becoming stunted? | 1=At the first two years of life  98=Don’t know  99=Other specify |
| 9 | What are the consequences of stunting for young children?  Mark all that are mentioned by the respondent  Don’t prompt | 1= Higher risk of severe infectious diseases  2=Poor educational performance  3=Weaker immune system  4=Low adult wages  5=Lost productivity  6=Excessive weight gain in later life  7=Increased risk of nutrition-related chronic diseases in adult life  98=Do not know |
| 10 | Have you heard about vitamin A deficiency or lack of vitamin A? | 1= Yes  2= No  99= don’t know |
| 11 | If Yes:  Can you tell me how you can recognize someone who lacks vitamin A in his or her body? | 1= Weakness/feels less energetic  2= be more likely to become sick (less immunity to infections)  3= Eye problems; night blindness (inability to see at dusk and in dim light), dry eyes, corneal damage, blindness  98= other  99= don’t know |
| 12 | Have you heard about iron-deficiency anemia? | 1= Yes  2= No  99= Don’t know |
| 13 | If Yes:  Can you tell me how you can recognize someone who has anemia? | 1= Less energy/weakness  2= Paleness/pallor  3= Spoon nails/bent nails (koilonychia)  4= More likely to become sick (less immunity to infections)  98= Other  99= don’t know |
| 14 | How can you recognize that someone is not having enough food? | 1= Lack of energy/weakness; cannot work, study or play as normal (disability)  2= Weakness of the immune system (become ill easily or becomes seriously ill)  3= loss of weight / thinness  4= children do not grow as they should (growth faltering)  98= other  99= do not know |
| 15 | What are the reasons why people are undernourished? | 1= Not getting enough food  2= food is watery, does not contain enough nutrients  3= Disease/ ill and not eating food  98= other  99= don’t know |
| 16 | Most women would benefit from two types of supplements, or tablets, during pregnancy. Which are they? | 1= Iron supplements  2= Folic acid supplements  98= other  99= don’t know |
| 17 | When I have questions on healthy nutrition, I know where I can find information on this issue. | 1=Yes  2=No |

### Attitudes and motives

Instruction: I am going to read you some statements attitudes and motives on about your nutrition and feeding. Please tell me if you agree with these statements.

**Remember, there are no correct answers! I would like to know your opinion.*

|  | What is the ***primary*** motive for the food you eat in a typical day? | 1=Food easy to prepare |
| --- | --- | --- |
|  |  | 2=Food easily available |
|  |  | 3=Food that smells, tastes, and looks nice |
|  |  | 4=Food with a pleasant texture |
|  |  | 5=Food that is relatively cheap |
|  |  | 6=Food that is healthy |
|  |  | 99=Other (Specify) |
| 2 | How good do you think it is to eat less, for example by eating smaller portions of food? | 1=Not good  2=You are not sure  3=Good |
| 3 | Can you tell me the reasons why it is not good? |  |
| 4 | How likely do you think you are to become overweight or obese? | 1=Not likely 2=You are not sure  3=Likely |
| 5 | How serious do you think it is to be overweight or obese? | 1=Not really serious  2=Neutral/serious  3=Serious |
| 6 | Poor diet during pregnancy and the first two years of child age can cause child stunting | 1= Do not agree  2= Neutral  3= Agree  98=Don’t Know |
| 7 | Colostrum (the "first yellowish milk") is not good for the baby and should be discarded | 1= Do not agree  2= Neutral  3= Agree  98=Don’t Know |
| 8 | It is good to give a baby only breastmilk and no other foods or liquids for the first six months | 1= Do not agree  2= Neutral  3= Agree  98=Don’t Know |
| 9 | If a child is sick (for example has fever/diarrhea) breastfeeding must be stopped | 1= Do not agree  2= Neutral  3= Agree  98=Don’t Know |
| 10 | A woman should consume iodized salt during pregnancy | 1= Do not agree  2= Neutral  3= Agree  98=Don’t Know |
| 11 | How good do you think is it to give diverse foods to your child each day?  **Nyaata gosa garaa garaa daa’ima keefi guyyaa guyyaa ti kennuun hagam gaariidha jettee yaadda?** | 1=not good 2=Not are not sure  3=good 98= don’t know  1=gaarii miti 2=adda hin baafne  3=gaariidha 98=Hin beeku |
| 12 | How likely do you think that your baby could be born underweight?  **Daa’ima ulfaatini isaa/ishee xiqqaa ta’e nin da’a jettee ni yaaddaa?** | 1=not good 2=Not are not sure  3=good 98= don’t know  1=gaarii miti 2=adda hin baafne  3=gaariidha 98=Hin beeku |
| 13 | How likely do you think your child is to be undernourished, that is they stop growing or lose weight?  **Daa’imni kee nyaataa ga’aa nyaatuu dhanuu irraan kan ka’e guddina dhaabe ykn qallate jette ni yaaddaa?** | 1=not good 2=Not are not sure  3=good 98= don’t know  1=gaarii miti 2=adda hin baafne  3=gaariidha 98=Hin beeku |

## Practices

### Access to clean water

| 1 | What is the main source of drinking-water for members of your household? | 1= Piped water into dwelling  2= Piped water to yard/plot  3= Public tap/standpipe  4= Tube well/borehole  5= Protected dug well  6= Unprotected dug well  7= Protected spring  8= Unprotected spring  9= Rainwater collection  10= Bottled water  11= Surface water (river, dam, lake, pond, stream, canal, irrigation channels)  99=Other specify |
| --- | --- | --- |
| 2 | What is the main source of water used by your household for other purposes, such as cooking and hand washing? | 1= Piped water into dwelling  2= Piped water to yard/plot  3= Public tap/standpipe  4= Tube well/borehole  5= Protected dug well  6= Unprotected dug well  7= Protected spring  8= Unprotected spring  9= Rainwater collection  10= Bottled water  11= Surface water (river, dam, lake, pond, stream, canal, irrigation channels)  99=Other specify |
| 3 | How long does it take to go there, get drinking water, and come back? | \|___\|___\|___\| (minutes) (0 if water is on premises)  98=Do not know |
| 4 | How long does it take to go there, get water other purpose, and come back? | \|___\|___\|___\| (minutes) (0 if water is on premises) |
| 5 | What do you usually do to the water to make it safer to drink? | 1=Boil  2=Add bleach/chlorine  3=Strain it through a cloth  4=Use a water filter (ceramic, sand, composite, etc.)  5=Solar disinfection  6=Let it stand and settle  7=Nothing  98=Do not know  99=Other (specify) |
| 6 | When do you wash your hands?  (Multiple Responses Possible)  PROBE: Make sure you capture all the responses by gently probing: “Is there any other time?” | 1 = After defecation/using toilet  2 = Before eating  3 = Before preparing food  4 = Before serving a meal  4 = Before praying  5 = Before feeding a child  6 = After changing a baby’s diaper/cleaning  7 = After touching dirty things  8 = After cleaning home  9 = When I wake up in morning  99= Other specify |

### Access to clean energy

| 1 | Which of the following household fuels do you use? | 1= Solid fuel in an open/three stone fire  2= Solid fuel in a traditional stove  3= Solid fuel in an improved stove (lakech, mirt)  4= Liquid or gas fuel and associated stove  5= Electricity and associated stove  99=Other specify |
| --- | --- | --- |
| 2 | Do you have access to electricity? | 1= No access to electricity at all  2= Access to third party charging only  3= Access to solar lantern or solar phone charger  4=Yes, intermittent electricity  5= Yes, with reliable electricity access  99=Other specify |
| 3 | If no access to electricity, how long does it take to go there, get firewood, and come back? | \|___\|___\|___\| (minutes)  98=Do not know |
| 4 | Total amount of money spent on energy per month | ---------------------(in birr) |

### Food Preparation

| 1 | In a typical day and for a typical food, h*ow many hours on average do you spend preparing, cooking, and cleaning up from meals each time?* | *\|__\|__\|__\| minutes (enter 0 if you do not prepare a food at home)*  Or  0= *Do not prepare a food at home*  1= less than 1 hour/day  2=between 1–2 hours/day  3=More than 2 hours/day  98=Do not know |
| --- | --- | --- |
| 2 | Have you attended a cooking demonstration in the last six months in your community? | 1=Yes  2=No  98=Do not know |

|  | Top 3 most frequently prepared dishes | Ingredients | Usual Cooking time (in minutes) |
| --- | --- | --- | --- |
| 2 | Dish 1 |  |  |
| 3 | Dish 2 |  |  |
| 4 | Dish 3 |  |  |

## Storage

| 1. | Do you use refrigeration as a method of preservation of foods? | 1. Yes  2. No 🡪 |
| --- | --- | --- |
| 2. | Do you dry foods as a method of preservation of food items? | 1. Yes  2. No |
| 3. | Do you use traditional or modern methods of persevering food items such as milk? |  |
| 4. | Do you use food rack as a method of preserving foods? | 1. Yes  2. No |

# **Module Seven:** **Social Welfare and Public Transfer Programs**

|  | In the last 12 months, has your household been receiving payments for doing public works for the PSNP? | | 1=Yes  2=No 🡪Skip to 7 |
| --- | --- | --- | --- |
|  |  | **Unit** |  |
|  | In the last 12 months, how many days have adult members in your household undertaken PSNP work? | (Days) |  |
|  | How much cash has your household received? | (Birr) |  |
|  | How much grain has your household received? Put the cash equivalent value? | (Birr) |  |
|  | How much cooking oil has your household received? Put the cash equivalent value? | (Birr) |  |
|  | How much pulses have the household received? Put the cash equivalent value? | (Birr) |  |

|  | In the last 12 months, has anyone in your household received other kinds of social transfers, such as pension, government transfer and support from non-governmental organizations (NGO)? | | | | 1=Yes  2=No 🡪Skip to module 8 |
| --- | --- | --- | --- | --- | --- |
|  | Type of social welfare and public transfers | How much cash has your household received in the last 12 months?  (Birr) | How much grain has your household received in the last 12 months?  (Put cash equivalent value)  (Birr) | How much cooking oil has your household received in the last 12 months?  (Put cash equivalent value)  (Birr) | How much pulses has the household received in the last 12 months?  (Put the cash equivalent value)  (Birr) |
|  | Pension |  |  |  |  |
|  | Social cash transfers |  |  |  |  |
|  | Government transfers other than PSNP |  |  |  |  |
|  | Transfers from NGO |  |  |  |  |
|  | Other social or public transfers |  |  |  |  |

# **Module Eight:** **Access to Basic Services**

|  |  |  |
| --- | --- | --- |
| 1 | What is the distance from the household's residence to the nearest place where there is ***a commercial bank***? | \|__\|__\|__\| Kilometers  \|__\|__\|__\| Minutes |
| 2 | What is the distance from the household's residence to the nearest place where there is a ***microfinance institution***? | \|__\|__\|__\| Kilometers  \|__\|__\|__\| Minutes |
| 3 | What is the distance from the household's residence to the nearest place where there is an ***SACCO***? | \|__\|__\|__\| Kilometers  \|__\|__\|__\| Minutes |
| 4 | What is the distance from the household's residence to the nearest place where there is an ***Insurance branch***? | \|__\|__\|__\| Kilometers  \|__\|__\|__\| Minutes |
| 5 | What is the distance from the household's residence to the nearest place where there is ***a health post***? | \|__\|__\|__\| Kilometers  \|__\|__\|__\| Minutes |
| 6 | How far is the household's residence from the ***WOREDA*** town? | \|__\|__\|__\| Kilometers  \|__\|__\|__\| Minutes |
| 7 | What is the type of main ***access road surface*** in this community?  1=Tar/Asphalt  2=Graded graveled  3=Dirt Road (Maintained)  4=Dirt Track  5=No road | \|__\|__\|__\| Kilometers  \|__\|__\|__\| Minutes |
| 8 | How far is it from the household's residence to the nearest ***tar/ asphalt road***?  *Write '0' if there is a Tar/Asphalt Road in the community.* | \|__\|__\|__\| Kilometers  \|__\|__\|__\| Minutes |
| 9 | How far is it the household's residence from to the ***nearest bus station***?  *IF IN COMMUNITY, WRITE "0".* |  |
| 10 | What is the distance from the household's residence to the ***nearest large weekly market***? | \|__\|__\|__\| Kilometers  \|__\|__\|__\| Minutes |
