# Supplementary material for: The influence of the food environment on diet quality: Insights from an extensive household survey in Ethiopia, focusing on women of reproductive age
Source: BMC Nutr. 2025 Jun 2;11:107. doi: 10.1186/s40795-025-01097-z (PMC12128275; doi:10.1186/s40795-025-01097-z)
Supplement: Supplementary file 5 — Additional file 5: Table S1: Overview of additional files supporting the diet quality and food environment analysis. [file 40795_2025_1097_MOESM5_ESM.docx]

Table S1: Overview of additional files supporting the diet quality and food environment analysis, Ethiopia

| **Additional File** | Title | Contents |
| --- | --- | --- |
| Additional file 1 | Supplementary tables: GDQS scoring methods and women’s 24-hour food group consumption (Ethiopia, n=1828) | Table S2: GDQS scoring and mean intake; Table S3: Percentage distribution of women by intake categories |
| Additional file 2 | Model fit and full Poisson regression results for dietary and food environment analysis, Ethiopia | Table S4: Poisson regression results; Table S5: Overdispersion test; Table S6: Model fit statistics |
| Additional file 3 | Supplementary figures on predicted diet quality scores by food environment (FE) among study samples (N=1828) | Fig S1: Normalized predicted dietary outcomes by FE; Fig S2: WDDS by FE and wealth; Fig S3: FVS by FE and wealth; Fig S4: GDQS by FE and wealth; Fig S5: HDDS by FE and wealth |
| Additional file 4 | Questionnaire utilized to conduct the household survey, Ethiopia | Appendix S1: Study questionnaire on food systems, dietary intake, and socioeconomic factors |
